# Supplementary material for: DNA signatures preserved in the official 1978 sample collection of the Shroud of Turin
Source: Sci Rep. 2026 Jul 9;16:21206. doi: 10.1038/s41598-026-60684-7 (PMC13350843; doi:10.1038/s41598-026-60684-7)
Supplement: Supplementary file 1 — Supplementary Material 1 [file 41598_2026_60684_MOESM1_ESM.pdf]

Supporting Information for:

DNA Signatures Preserved in the Official 1978 Sample Collection of the Shroud of Turin

Gianni Barcaccia [1] † *, Nicola Rambaldi Migliore [2] †, Giovanni Gabelli [1] †, Vincenzo Agostini [2], Fabio Palumbo [1], Elisabetta Moroni [2], Valeria Nicolini [2], Liangliang Gao [3], Grazia Mattutino [4], Andrew Porter [5], Pawel Palmowski [5], Noemi Procopio[6], Ugo A. Perego [7], Massimo Iorizzo [3], Timothy F. Sharbel [8], Pierluigi Baima Bollone [9], Antonio Torroni [2], Andrea Squartini [1], Alessandro Achilli [2] *

[1] Laboratory of Genomics, Research Centre for Agriculture "Maurizio Borin", Department DAFNAE, University of Padova; Campus of Agripolis, Legnaro (PD), Italy
[2] Department of Biology and Biotechnology "L. Spallanzani", University of Pavia; Pavia, Italy
[3] Plants for Human Health Institute, North Carolina State University, Kannapolis, NC 28081, USA
[4] Laboratory of Criminalistic Science "Carlo Torre", Department of Sciences of Public Health and Pediatrics of the University of Torino; Turin, Italy
[5] Newcastle University Protein and Proteome Analysis (NUPPA) Facility, Medical School, Newcastle University, Newcastle upon Tyne, UK
[6] Research Center for Field Archaeology and Forensic Taphonomy, University of Lancashire, Preston, UK
[7] Southeastern Community College, West Burlington, Iowa 52655, USA
[8] Department of Plant Sciences, College of Agriculture and Bioresources, University of Saskatchewan, SK S7N 5A8, Canada
[9] Professor emeritus, University of Torino; Turin, Italy

†These authors contributed equally to this work

*Corresponding authors. Gianni Barcaccia (ORCID ID: 0000-0001-7478-5048), Alessandro Achilli (ORCID ID: 0000-0001-6871-3451)
Email: gianni.barcaccia@unipd.it; alessandro.achilli@unipv.it

This PDF file includes:

Supporting Text
References

Supplementary Figures S1 to S5
Supplementary Tables S1 to S5
Supplementary Document S1_Release by Prof Baima Bollone
Legends for Datasets S1 to S9

Other supporting materials for this manuscript include the following:

Datasets S1 to S9

Supporting Text

Sampling of the Turin Shroud by Prof. Pierluigi Baima Bollone

Sampling of the Turin Shroud was performed from several areas of the linen cloth on the night of Sunday, 8 October 1978, by Prof. Pierluigi Baima Bollone.
On 5 October 1978, an inspection was conducted in the areas of the Royal Palace of Turin designated for sampling and analytical activities. The selected premises included the Library, the Yellow Room, the Hall of the Battle, and part of the corridor connecting the Chapel of the Holy Shroud to the Hall of the Battle. It was established that the Turin Shroud would be placed in the library for the sampling procedures, while the adjacent rooms would be used for storage of the scientific instrumentation. In the days preceding the examinations, extensive cleaning procedures were carried out involving floors, surfaces, curtains, tapestries, doors, and walls [1]. Between 10:25 p.m. and 11:15 p.m. on 8 October 1978, the Turin Shroud was transferred to the library and then positioned on a specially designed aluminum table manufactured by Nuclear Technology Inc. Only a limited number of researchers previously authorized by the Supervisory Commission, chaired by Prof. Luigi Gonella of the Polytechnic University of Turin, were admitted to the sampling procedures following submission of a detailed experimental protocol. At 11:20 p.m., Dr. Max Frei initiated adhesive tape sampling procedures, which concluded at 11:55 p.m. Subsequently, between 12:15 a.m. and 12:45 a.m. on 9 October 1978, Prof. Baima Bollone performed sampling of several fibers using sterile microsurgical instruments, collecting fibers from both warp and weft components from predetermined areas of the cloth. Four additional loose fibers protruding from the textile surface were also collected. Subsequently, from 1:40 a.m. to 7:30 a.m., Mr. Giovanni Riggi di Numana carried out aspiration sampling procedures using sterilized instruments [2,3].
It is worth noting that neither a dedicated sterile sampling chamber nor a controlled-atmosphere enclosure was employed during the procedures, as research infrastructures specifically designed to handle ancient specimens under contamination-controlled conditions were not available in 1978. Nevertheless, substantial preventive measures were adopted to minimize environmental contamination, including extensive cleaning of the rooms, sterilization of the sampling instruments, and restricted access limited exclusively to authorized personnel in the adjacent areas. The available historical documentation does not indicate the use of specific personal protective equipment beyond standard laboratory precautions, nor does it record the storage of the collected fibers in nitrogen-filled containers. All samples were preserved according to the protocols commonly adopted 50 years ago in forensic medicine laboratories.

Limitations of the study

We encountered methodological challenges in terms of data production due to the very limited amount of source material and significant DNA degradation. Our initial goal was to verify the presence of human blood traces on the shroud fibers. Next, we examined the possible presence (and characteristics) of DNA reads of human origin. Finally, we determined the number of operational taxonomic units (OTUs) derived from environmental and individual contaminants associated with all the organisms that have come into contact with this shroud over the centuries. Finally, we tried to correlate the obtained results with available historical information, the geographic regions of probable origin, and the modern distribution of the species.

This study also faced significant challenges in data analysis, primarily due to the short length of the reads and the limitations of the reference DNA databases. These two factors likely contributed to the lower assembly metrics observed in the metagenome reconstruction and the difficulties encountered in identifying taxonomic entities univocally, particularly regarding animals and plants. These technical difficulties were compounded by the peculiarity of the proposed objectives: assigning minimal amounts of genetic material to any of the known species. These challenges required the adoption of stringent filters for species identification. Therefore, it is possible that some false negatives are present. Although relying on short contigs is advantageous for detecting minimal traces of genetic material, resulted in the alignment of many contigs in genomic regions largely conserved among taxa, eventually reducing the number of informative sequences.

References

1. Riggi di Numana, G. *Rapporto Sindone 1978–1982*. (Il Piccolo Editore, Torino, 1982).
2. Baima Bollone, P. Leggendo il resoconto pubblico delle perizie dell'ottobre 1978 sulla Sindone. *Sindon* ***21***(28), 9–14 (1978).
3. Baima Bollone, P. & Giusti, F. *La notte della prova. Sindone, la conferenza dimenticata e il verbale ritrovato*. (Kemet Edizioni, Trieste, 2024).

Figures

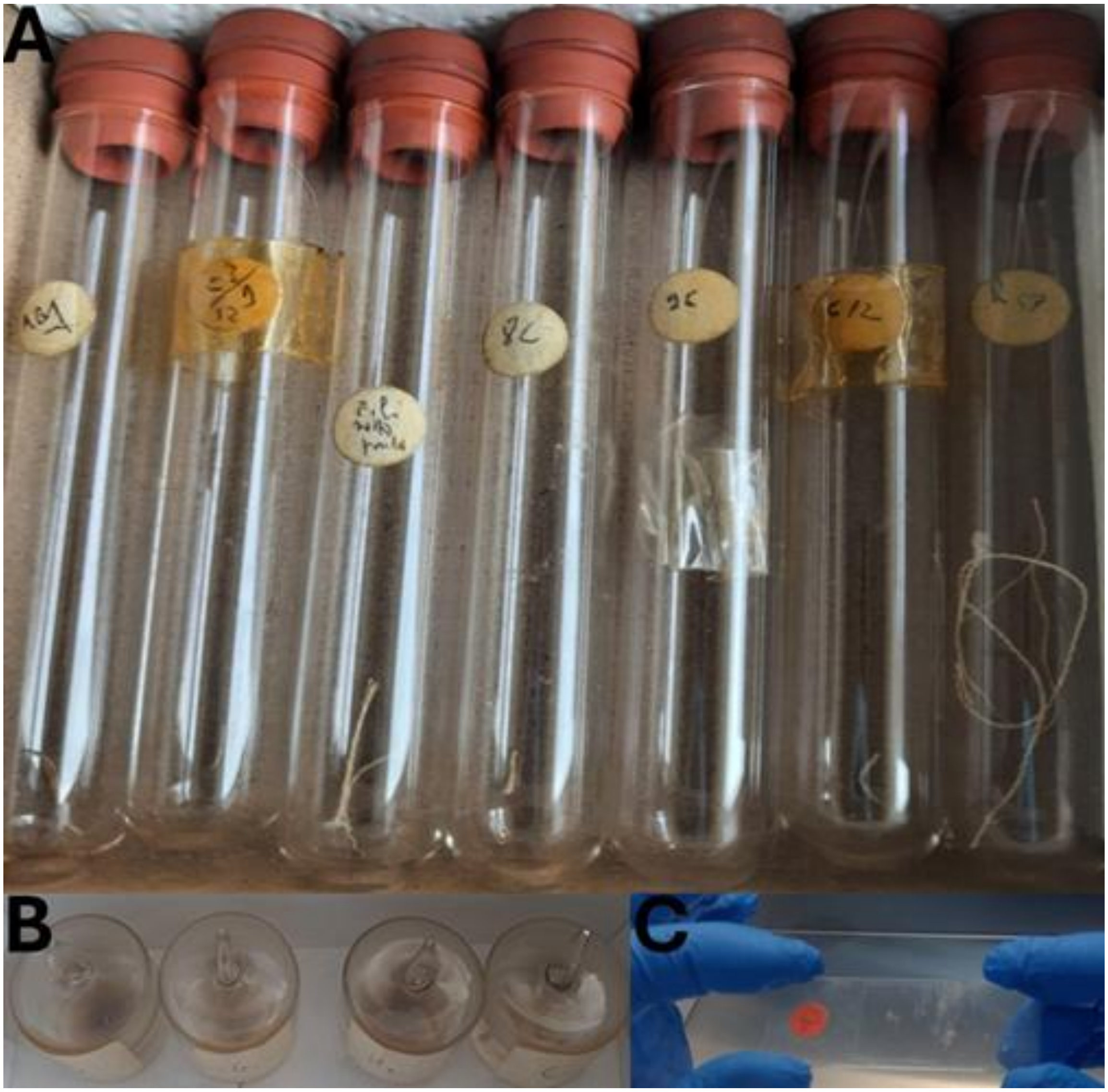

Figure S1. Pictures of the TS samples provided by Prof. Baima Bollone (collector) and analyzed in this study.
A) Linen cloth fragments conserved in glass tubes, see also Supplementary Table 1. The sample "*fili sotto piede*" (literally "threads under foot") has been renamed as "A11", while the tube labeled as "R58" refers to linen threads collected from the Reliquary (Prof. Baima Bollone report). B) Vacuum dust preserved in glass jars. C) Small biological sample on a microscope slide with a cover slip. See Table S1 for additional information.

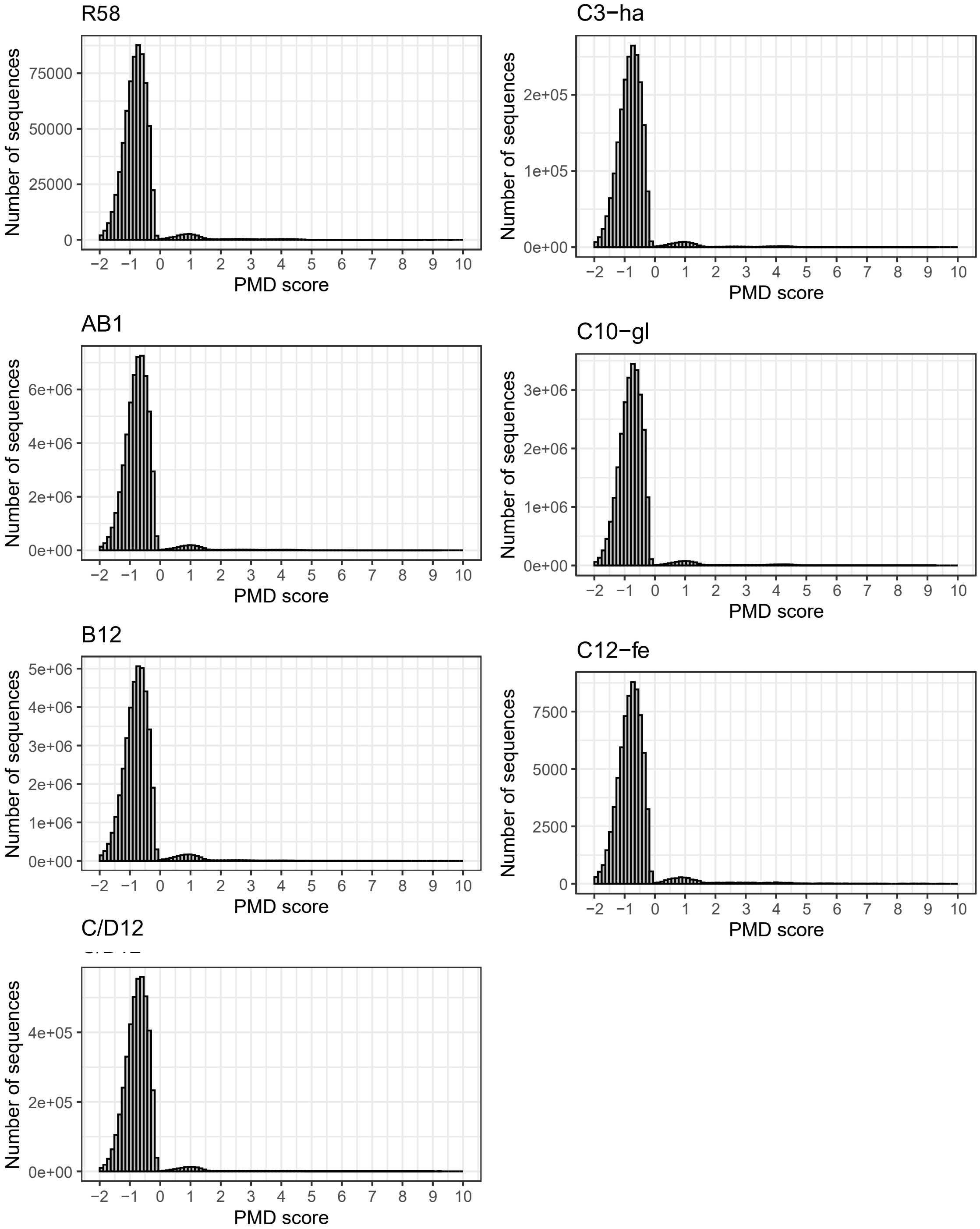

Figure S2. PMDs distribution in the seven TS samples aligned to the human reference genome.

The maximum-likelihood probabilistic model implemented in PMDtools [Skoglund et al. (https://doi.org/10.1073/pnas.1318934111) was applied to assign post-mortem damage (PMD) scores to each read.

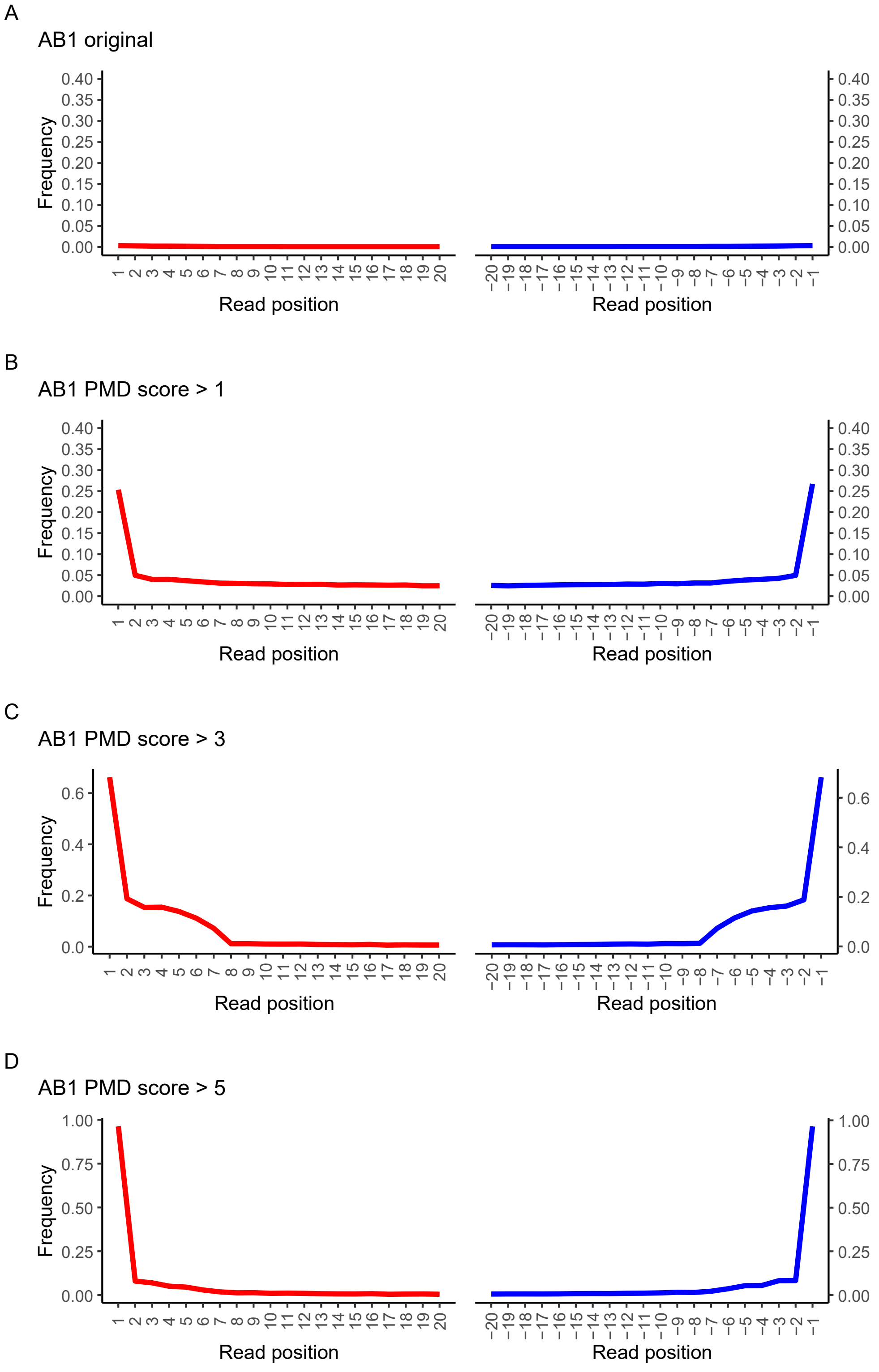

Figure S3. Damage pattern analysis of sample AB1 mapped to the human reference genome.

The damage pattern analysis was computed on A) the total mapped reads, B) the reads showing a PMDS greater than 1 (1.14% of the total), C) the reads showing a PMDS greater than 3 (0.25% of the total), D) the reads showing a PMDS greater than 5 (0.076% of the total).

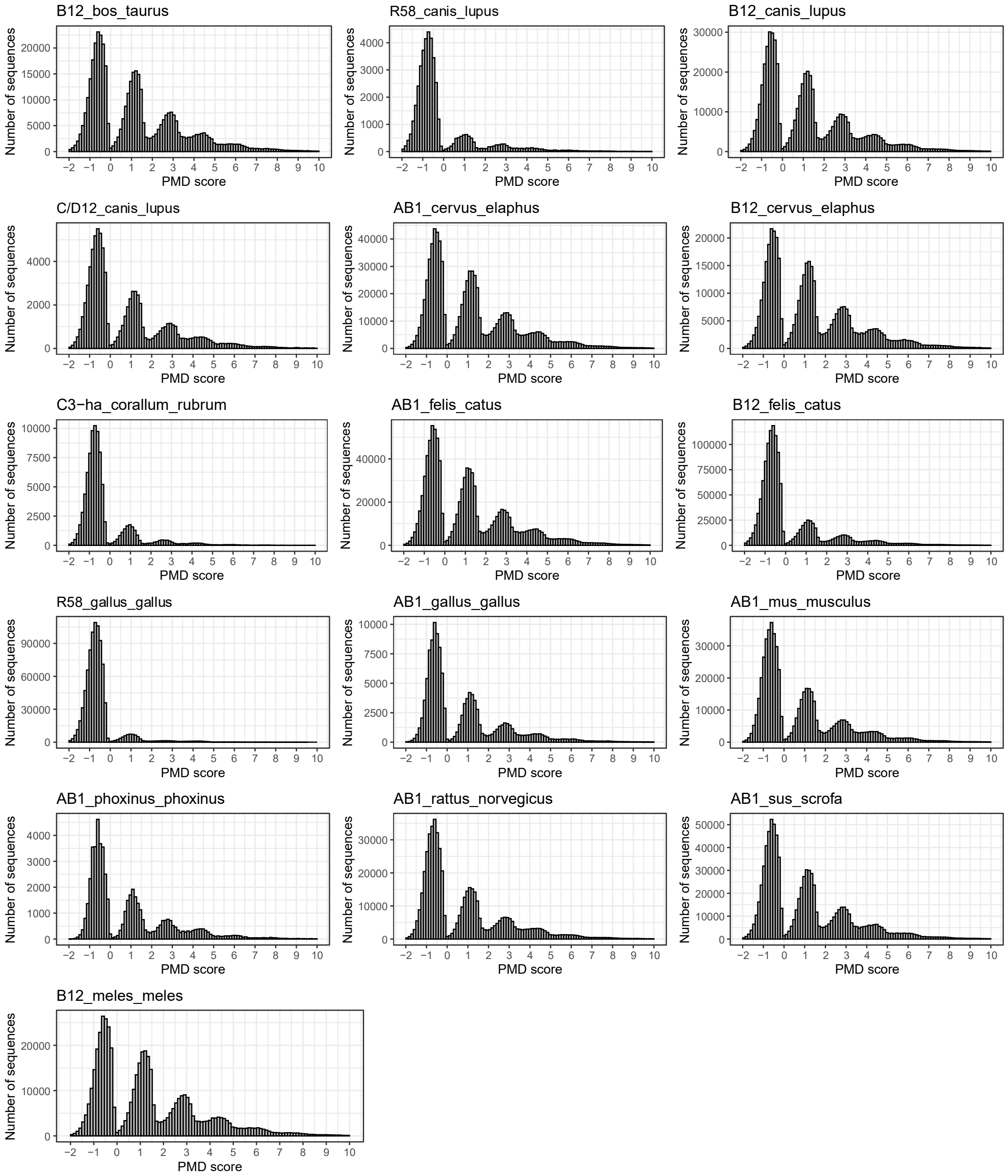

Figure S4. PMDs distribution in the TS samples aligned to different animal species. The distribution of the post-mortem damage (PMD) scores was computed only for those TS samples showing more than 100 contigs using the contig-BLAST approach (Dataset_S8).

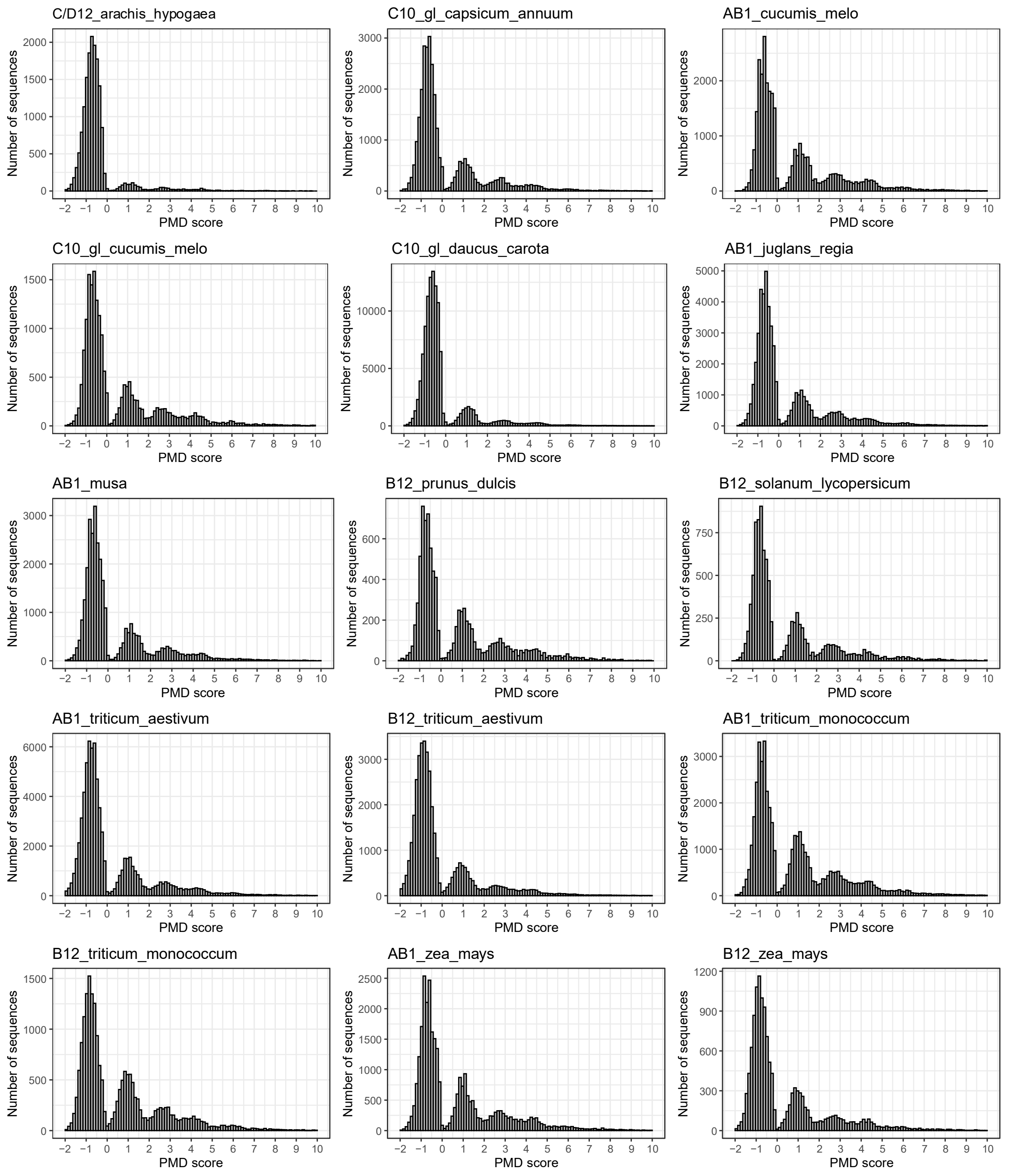

Figure S5. PMDS distribution in the TS samples aligned to different plant species. The distribution of the post-mortem damage (PMD) scores was computed only for those TS samples showing more than 100 contigs using the contig-BLAST approach (Dataset_S8).

Tables

Table S1. List of samples analyzed in this study (see Figure S1 for further details).

Sample ID[a]	Shroud section	Specimen	Genomic data	Human endogenous content (%)	Human average depth (X)	Meta genomics
AB1	lateral edge	linen fragment	yes	47.18	1.38	yes
C/D12	feet	linen fragment	yes	5.64	0.10	yes
A11	feet	linen fragment	no	n.a.	n.a.	n.a.
C8	arm	linen fragment	no	n.a.	n.a.	n.a.
C9	arm	linen fragment	no	n.a.	n.a.	n.a.
C12	feet	linen fragment	no	n.a.	n.a.	n.a.
R58	Reliquary	linen fragments	yes	0.57	0.02	yes
C3_ha	hands	vacuum dust	yes	1.93	0.05	yes
C6_fa	face	empty	no	n.a.	n.a.	n.a.
C10_gl	glutei	vacuum dust	yes	18.68	0.54	yes
C12_fe	feet	vacuum dust	yes	0.05	0.00	yes
B12	feet	microscope slide	yes	39.60	1.14	yes

[a] The first code refers to the coordinates of the graphic map of the TS reported in the Figure S2 panel C of Barcaccia et al. [3]. The second code, if present, underlines the area of the body image from which the dust particles were vacuumed: hands (ha), face (fa), glutei (gl), feet (fe).

Table S2. Kinship analyses.
Genomic relatedness between the three best-performing TS samples (AB1, B12 and C10-gl) and the controls (Lab operator and collector).

ind1	ind2	BREADR	READ2	KIN (>0.05X)
AB1	B12	First_Degree	First_Degree	First_Degree
AB1	collector	First_Degree	Same_Twins	First_Degree
AB1	C10-gl	First_Degree	First_Degree	First_Degree
AB1	operator	Unrelated	Unrelated	Unrelated
B12	collector	First_Degree	First_Degree	First_Degree
B12	C10-gl	First_Degree	First_Degree	First_Degree
B12	operator	Unrelated	Unrelated	n.a.
C10-gl	collector	Same_Twins	Same_Twins	First_Degree
C10-gl	operator	Unrelated	Unrelated	Unrelated

Table S3. Summary of the Kraken2 results.
Total number of analyzed reads and percentages of reads assigned to different domains for each sample.

Sample	N. reads	Unassigned	Bacteria	Viruses	Archaea	Eukaryota	*Homo*
AB1	132 435 925	17.2	11.0	0.1	0.0	71.7	68.5
C/D12	85 726 122	54.5	31.8	0.0	0.1	13.6	12.7
R58	135 258 039	72.0	26.2	0.0	0.1	1.7	1.3
C3_ha	124 148 569	69.2	27.0	0.0	0.1	3.7	3.2
C10_gl	161 694 229	50.2	18.5	0.0	0.0	31.3	29.9
C12_fe	148 328 250	71.6	27.9	0.0	0.1	0.4	0.1
B12	118 623 300	25.9	10.0	0.0	0.0	64.0	62.1

Table S4. Summary metrics of the metagenomic assembly for each sample.
The columns describe the total number of contigs obtained, the grand total of assembled base pairs, the N50 value of the assembly, the lengths of the longest and shortest contigs, and the average contig size.

Sample	Total n. of contigs	Total bp	Min contig length (bp)	Max contig length (bp)	Avg contig length (bp)	N50 (bp)
AB1	4 711 086	1 143 646 932	118	68 555	242	240
C/D12	284 933	80 382 933	146	155 175	282	240
R58	348 254	80 015 682	151	254 563	229	194
C3_ha	353 365	89 037 482	151	254 543	251	213
C10_gl	955 934	196 561 234	150	254 691	205	185
C12_fe	298 238	73 208 206	150	254 505	245	214
B12	4 693 106	1 169 249 982	118	271 010	249	249

Table S5. Summary of the BLASTn analysis results.
Total number of analyzed reads and the percentage of reads assigned to different domains for each sample.

Sample	N. contigs	Unassigned	Bacteria	Viruses	Archea	Eukaryota	*Homo*
AB1	4 711 086	7.1	3.7	0.1	0.0	89.2	81.8
C/D12	284 933	25.2	30.3	0.1	0.0	44.4	28.3
R58	348 254	83.9	6.6	0.1	0.0	9.4	6.5
C3_ha	353 365	66.1	14.2	0.1	0.0	19.6	12.9
C10_gl	955 934	31.3	6.6	0.1	0.0	62.1	49.5
C12_fe	298 238	95.1	4.1	0.1	0.0	0.7	0.6
B12	4 693 106	6.2	3.3	0.0	0.0	90.5	39.0

Documents

Supplementary Document S1: Original letter (in Italian) written by Prof. Pierluigi Baima Bollone to the corresponding authors and some other coauthors of the article "DNA Traces on the Shroud of Turin: Metagenomics of the 1978 Official Sample Collection". This letter, dated 1 February 2024, describes the specimens of the Turin Shroud, deriving from the 1978 original samplings, which were made available to Prof. Gianni Barcaccia and Prof. Fabio Palumbo of the University of Padova, and to Prof. Antonio Torroni, Prof. Alessandro Achilli and Prof. Anna Olivieri of the University of Pavia, for the purposes of research, with the commitment to perform DNA sequencing.

LIBERATORIA

Il sottoscritto Prof. Pierluigi Baima Bollone, residente a Torino in Via Pastrengo 30, dichiara che in data 20 settembre 2023 ha consegnato ai professori Antonio Torroni, Alessandro Achilli e Anna Olivieri dell'Università di Pavia e ai professori Gianni Barcaccia e Fabio Palumbo dell'Università di Padova un totale di 5 provette e 4 barattoli in vetro contenenti campioni personalmente prelevati in data 9 ottobre 1978 nel corso di analisi autorizzate della Sindone. I campioni appena citati sono stati da me messi a disposizione di questi docenti ricercatori con l'impegno di eseguire reazioni di sequenziamento del DNA di origine umana, animale e vegetale eventualmente isolabile e tecnicamente analizzabile allo scopo di ricostruire aplotipi mitocondriali e cloroplastici. Il sottoscritto dichiara altresì di essere pienamente consapevole che i protocolli disponibili ed utilizzabili in laboratorio per le estrazioni del DNA genomico sono da considerarsi distruttivi e che i campioni consegnati saranno impiegati in toto e pertanto non potranno essere né preservati né restituiti.

Torino, 1 febbraio 2024 Prof. Pierluigi Baima Bollone

The English translation of the original Italian text of the Prof. Baima Bollone's letter is reported below:

RELEASE

The undersigned, Professor Pierluigi Baima Bollone, residing in Turin at Via Pastrengo 30, hereby declare that on September 20, 2023, I delivered to Professors Antonio Torroni, Alessandro Achilli, and Anna Olivieri of the University of Pavia, as well as to Professors Gianni Barcaccia and Fabio Palumbo of the University of Padua, a total of 5 test tubes and 4 glass jars containing samples personally collected on October 9, 1978, during authorized analyses of the Shroud. The abovementioned samples have been made available to these researchers with the commitment to perform DNA sequencing reactions of human, animal, and plant origin that may be isolated and technically analyzable with the aim of reconstructing mitochondrial and plastid haplotypes. I further declare that I am fully aware that the protocols available and usable in the laboratory for genomic DNA extractions are to be considered destructive, and that the samples delivered will be used in their entirety and therefore cannot be preserved or returned.

Turin, 1 February 2024 Prof. Pierluigi Baima Bollone

Legends for Datasets S1 to S9

Dataset S1 (separate file). Summary of genomic data (and 14C radiocarbon dating).

Dataset S2 (separate file). Summary of mitogenome data.

Dataset S3 (separate file). Summary of proteomics analyses

Dataset S4 (separate file). Summary of metagenomic analyses (Archea, Bacteria, Fungi)

Dataset S5 (separate file). Outputs of Kraken.

Dataset S6 (separate file). Outputs of MetaPhlAn

Dataset S7 (separate file). Complete list of taxa identified using the contig-BLAST approach.

Dataset S8 (separate file). Summary of metagenomic analyses using the contig-BLAST approach (plant and animal taxa).

Dataset S9 (separate file). Contigs that matched GenBank and RefSeq sequences with potential contamination, with the relative BLASTn results. Each sheet accounts for one sample according to GenBankl or Refseq, per sheet name.
