# Supplementary material for: DNA signatures preserved in the official 1978 sample collection of the Shroud of Turin
Source: Sci Rep. 2026 Jul 9;16:21206. doi: 10.1038/s41598-026-60684-7 (PMC13350843; doi:10.1038/s41598-026-60684-7)
Supplement: Supplementary file 6 — Supplementary Material 6 [file 41598_2026_60684_MOESM6_ESM.html]

Javascript must be enabled to view this page.

magnitude
magnitudeUnassigned

AB1\_Kraken
C3\_Kraken
A11\_Kraken
C3\_ha\_Kraken
C10\_gl\_Kraken
C12\_fe\_Kraken
B12\_Kraken

13243592585726122135258039124148569161694229148328250118623300

114120814854333767658806903205438771923
9495456011675677230162346267765053578364154675939559

7511621
0000000

7511621
0000000

7511621
0000000

7511621
0000000

7511621
0000000

7511621

0000000
1221049203

1221049203
0000000

1221049203
0000000

1221049203

30133024321827
0000000

0000000
30133024321827

2000003
30133024321827

132481049

36105933

6582613

6897109

215303352214323216388
158473518

0000000
919812810014586135

919812810014586135
0000000

919812810014586135
0000000

0000000
919812810014586135

919812810014586135

0000000
123147220107175125235

0000000
123147220107175125235

123147220107175125235
0000000

123147220107175125235
0000000

123147220107175125235

526221330297489357477
0000000

526221330297489357477
0000000

526221330297489357477
0000000

0000000
526221330297489357477

526221330297489357477

0000000
8696298638653601610443924442

8696298638653601610443924442
0000000

0000000
8696298638653601610443924442

8696298638653601610443924442
101831117

0000000
11302001119262078706

11302001119262078706

7556278537463506548343133719
110811591888171321922351864

40886396723019287

757232200211540255505

19743365113123155

667869212540542367

426748110733775291

3993110514101232164815481250

35296300105991007710090134893791
0060000

768272149194295413359901104
0000000

768272149194295413359901104
5370021

0002000
47612562243195620472800538

0000000
298275489389590609250

298275489389590609250

33127330
1789811754156514572191288

561239414814620087

1198551648141013081988201

28714622669233920863188565
0000000

0000000
28714622669233920863188565

28714622669233920863188565

0000000
2761357956745782595774992687

0000000
2761357956745782595774992687

2761357956745782595774992687
0000000

0000000
2761357956745782595774992687

2761357956745782595774992687

49761781044921394
4921725655618330312661484913013397403

13474744937557095141634
12222243595115

72151840270140
9543293475235253841147

354144101157141109452
0000000

0000000
354144101157141109452

354144101157141109452

0000000
528170228326357275555

0000000
528170228326357275555

528170228326357275555

0000000
271123122197175125372

0000000
271123122197175125372

0000000
271123122197175125372

271123122197175125372

11432264485575144221105
0000000

0000000
11432264485575144221105

39121331241
11432264485575144221105

0000000
625124255309285219727

625124255309285219727

479101191235226191337
0000000

479101191235226191337

1849110718751917246320491786
74011206

8087031014110212281339934
0000000

159658016
8087031014110212281339934

0000000
384438641703714878502

384438641703714878502

409256367394506461416
0000000

409256367394506461416

10344008618041233710846
0000000

15206101
10344008618041233710846

57202944331546
744143267316611257657

22374149127226150202

37841279228923342

8686253636967

0000000
275255594482621453188

275255594482621453188

486357541381457527013100578997391796
0000000

0000000
486357541381457527013100578997391796

486357541381457527013100578997391796
0000000

19881913915765551506
486357541381457527013100578997391796

48186853086132182530688377348386504

181877812081443107515062826

683831101078088960

0000000
97955086192010571010688

97955086192010571010688
0000000

97955086192010571010688
0000000

97955086192010571010688
29121213121120

534336495545562610328

416202354362483389340

0000000
13245031296312

0000000
13245031296312

0000000
13245031296312

0000000
13245031296312

13245031296312

0000000
406118195198242110240

406118195198242110240
0000000

406118195198242110240
0000000

0000000
406118195198242110240

0000000
406118195198242110240

406118195198242110240

1000001
29155846102835

85351841520
0000000

0000000
85351841520

0000000
85351841520

85351841520

1000000
2010232861314

16165262
0000000

0000000
16165262

16165262

0000000
1847234712

0000000
1847234712

1847234712

2211121171897
120478165188239970148939699896884

112918143085967715446172488291726
0000000

0000000
112918143085967715446172488291726

0000000
44235225494545

44235225494545
0000000

44235225494545

112874142855915712946123483791681
528107356

51014752289213821413189625
0000000

51014752289213821413189625

11188612313282843474316070090637
0000000

11188612313282843474316070090637

473469788637819943413
0000000

473469788637819943413

5902507
7538209922512530274921075151

0000000
3243118014791390209814462248

3243118014791390209814462248
0000000

3243118014791390209814462248
386157126108160107199

92233131413069

4652048010820587272

289727510417339165

178228668995192

1673350449140125

5792830372031

185488773130102144

92192417533152

20557113110142123141

16549925812163154

597332523496627627590

1468459609640107

82274121472826

137446462765881

429091077211386466612896
12527459102

0000000
41307866329674305362763

20110001
41307866329674305362763

40867095599193924842727

2956342711149

13212821273826

14811913897157115131
0000000

0001220
14811913897157115131

3561624233426

31262318371231

55256328694149

27623626262625

1615249717698601152194
0000000

0000000
1615249717698601152194

0000000
1615249717698601152194

0000000
1615249717698601152194

0000000
1615249717698601152194

1615249717698601152194

0000000
9078335310884178177587039925414838901916792673652976

9078335310884178177587039925414838901916792673652976
0000000

9078335310884178177587039925414838901916792673652976
0000000

9078335310884178177587039925414838901916792673652976
0000000

9078335310884178177587039925414838901916792673652976
0000000

9078335310884178177587039925414838901916792673652976

13820196921282472797612948325
14328664036894147584055191231526391746964022

19035635059235992524810156612333122592
13797283893613923823866821195548371014928802

1091149559540330476
7997412502111761108232495965340767

0000000
3990253813872461817620329326103

161112190152219726857143410734
3990253813872461817620329326103

1231717171195143055869648177

1147414741155121651778957192

7368169322492292443021734937
114244539693676

0000000
335292513671315227513312311

335292513671315227513312311

0000000
390274483793820868062550

390274483793820868062550

316135279500040771004241579251
1435225185148429165455

23294737586221285767688
25609403735222823704029086010

4341889136394821357972548

18939267514011253362013442774

0000000
4569101712931106257310842786

4569101712931106257310842786

3756143019381642288719122364
0000000

3756143019381642288719122364
0000000

3756143019381642288719122364
0000000

3756143019381642288719122364

9908227521802561423216646022
6721834

0000000
602512721068116018257293059

0000000
602512721068116018257293059

602512721068116018257293059

0000000
38779961110140023999322959

0000000
38779961110140023999322959

38779961110140023999322959

0000000
12893287652973865861438448636

12893287652973865861438448636
1202525744955656

0000000
7671179526882417537323935128

7671179526882417537323935128

5102105623521404314613953452
0000000

5102105623521404314613953452

2476467349143906615948924716011
160245619881280

15158348743804803879142619532
0000000

399118290224293246243
15158348743804803879142619532

28427551019101619689981889

186254269772311946261322

10055207223742840533623916078

7138226231872982493833314881
0000000

0000000
7138226231872982493833314881

7138226231872982493833314881

230896115201262213116431518
0000000

0000000
230896115201262213116431518

230896115201262213116431518

8145276344723696630142795836
0000000

8145276344723696630142795836
0000000

0000000
8145276344723696630142795836

8145276344723696630142795836

1983544725515526720395507511560
0000000

27051556585948134
1983544725515526720395507511560

22748474651848145
98501780247622981382321125064

351283513461278221911612161

61118971083974110869032758

0000000
5861176723842196394323134259

5861176723842196394323134259

385487460070817706022103
0000000

385487460070817706022103

173163828411539819927423411837
14255999

29715621410
14319286727022801794327279847

4925134814551528311216653404
0000000

4925134814551528311216653404

0000000
9365151212321267481010586433

9365151212321267481010586433

0000000
298395914081175197514981981

298395914081175197514981981
0000000

298395914081175197514981981

3314068607710701717638673421754
236172733846124

8401155216331597456414465519
0000000

0000000
8401155216331597456414465519

8401155216331597456414465519

0000000
9826234824492278506822195874

9826234824492278506822195874
0000000

9826234824492278506822195874

0000000
4511128120611362316014813170

0000000
4511128120611362316014813170

4511128120611362316014813170

0000000
10166166215401747476215827067

0000000
10166166215401747476215827067

10166166215401747476215827067

12511377252384849915955588543
0000000

12511377252384849915955588543
190465941853882

0000000
7946239931382923587933445632

7946239931382923587933445632

0000000
4375132720411885319521762829

4375132720411885319521762829

358502104324148736155619225635151686276238
21146516414037137

61056144941763620569342411545050345
354672103123147237154038223156150046273621

51791766334910672362838552295
1978798821628232187182811950912078

6290332455939319633469574090

83184792734012196831986975693

976252388947851810370103607792
0000000

976252388947851810370103607792

2442111015471462202117912001
0000000

2442111015471462202117912001

162692314101476181417211436
2889181528142780344934562453

558268539427629587448

70562486587710061148569

12611194155154158192
73431148661424514538382651613650658

6076210631093041492633634215

67229126491104211342331851261546251

0000000
9881336451404546809558206743

9881336451404546809558206743

1931104918561509176319151348
14426402951524742918055039870

144243057255010756341203

22176391121847137910181274

8836191116031836496319366045

0000000
154144310735375318443747620577

154144310735375318443747620577

0000000
14883395049054899945158319932

14883395049054899945158319932

68108202713134525513410692798252266
123877365765727347128749025258496627

40944888312562962819133940631703

4764169727912634384228914273

10061572510575935310858123058385

0000000
6824348959435138645861304545

6824348959435138645861304545

3619115514481517233916032480
0000000

3619115514481517233916032480
0000000

3619115514481517233916032480

0000000
3279149023706411915084344822077

3279149023706411915084344822077
0000000

3279149023706411915084344822077
0000000

3279149023706411915084344822077

33430788710411882019761987522217
0000000

33430788710411882019761987522217
48814423617

8899209751493661669447556225
3042172659693808918177909119979

240276087793215899401602

14217201713651449646212558755

4903239123022047343221413397

296161470472715617782221
0000000

296161470472715617782221

2844374879648906216950927220611
0000000

2844374879648906216950927220611
307140284159250143265

2520103816321572213416091869
0000000

2520103816321572213416091869

2004644035088486710479468514746
3569107320041677269617252411

92046010017681013982601

249598712201265187213421514

1306218838631157489863610220

0000000
4417147120361804313120862944

4417147120361804313120862944

1153435608660956749787
0000000

1153435608660956749787

0000000
1583629819735263350268627033216107413

1583629819735263350268627033216107413
6020116573798328064574221

13001208719271848637414998504
0000000

13001208719271848637414998504

11010298028682934702230158992
8331241324192370545425836621

969199207255673210984

17103682423098952221387

25720704266087604414645608515936
168385778437623607919837339965

246495737013043145

1949892278979011308711062

3014188689891118318912138

3673133956566623565472626

0000000
4580151522001937319922123033

4580151522001937319922123033

0000000
7759150915261302405013004898

7759150915261302405013004898

163063350386337819151396410919
0000000

163063350386337819151396410919

10194316641744122721945177145
652174195174403207481

35919559621083232810052540

308186711961260200212942127

2870117018211605248620111997

0000000
4398159420201831305319862869

4398159420201831305319862869

0000000
4946104012571111263710583199

4946104012571111263710583199

0000000
4828130916682423298715643326

4828130916682423298715643326

2684934571912232711279141518083
0000000

2684934571912232711279141518083

0000000
2275145995024438311848414416288

2275145995024438311848414416288

7251190326812538504925474820
0000000

7251190326812538504925474820
0000000

7251190326812538504925474820
285954169769317905661844

165837032837610583211169

273499216561469220116601807

69620138321362013087332861361240367
0000000

69620138321362013087332861361240367
5791325790196163355

1180423201134123354549597099
0000000

1180423201134123354549597099

2926656105180495414770540319425
0000000

2926656105180495414770540319425

15899237822832386503724775619
0000000

15899237822832386503724775619

12072339249664424782946107869
0000000

12072339249664424782946107869

5371123715711375346012083690
0000000

5371123715711375346012083690
0000000

5371123715711375346012083690
0000000

5371123715711375346012083690

11186916032
3406780779714865413887960811917

116634406750563811023976858448
0000000

0000000
116634406750563811023976858448

116634406750563811023976858448

0000000
22293366322032264363219233437

22293366322032264363219233437
0000000

22293366322032264363219233437

584414241684150638109715723404
0000000

0000000
584414241684150638109715723404

584414241684150638109715723404
0000000

584414241684150638109715723404

1878042975341507211316552512254
164521209

32232922381815
5897242438753444540942124307

20895721004886154710231580
0000000

20895721004886154710231580

0000000
3776182928422536382431712712

3776182928422536382431712712

0000000
12867186914611626589513137938

0000000
12867186914611626589513137938

12867186914611626589513137938

0000000
1955938204097373210609404913358

0000000
1955938204097373210609404913358

0000000
1955938204097373210609404913358

1955938204097373210609404913358

85321960602459
24614807693801116416397927515548

1516249056820775811231703010177
1203645511014680

0000000
6258212125382374455724324215

6258212125382374455724324215

0000000
246189112242859186716841621

246189112242859186716841621

278391215471184231414631845
0000000

278391215471184231414631845

354094514661290239214052416
0000000

354094514661290239214052416

0000000
9367313925413346510622215312

9367313925413346510622215312
1623373396995828320911

3975120514711497237112762363

3769156167485419076252038

0000000
495575082474024086293006

495575082474024086293006
0000000

495575082474024086293006
0000000

495575082474024086293006

0000000
13374313347313821830038769130

0000000
13374313347313821830038769130

13374313347313821830038769130
0000000

13374313347313821830038769130

41083525024328246
60476139721936516753354521820545719

0000000
2939109220661854253621271929

0000000
2939109220661854253621271929

2939109220661854253621271929

2434364429890896616876979823923
22844607216047341

155344284452811576851118
0000000

155344284452811576851118

1727518103875616429791211
0000000

1727518103875616429791211

13272784523507624123223
67261375141513463444120311617

1155230337280550290816

263847026436112942296156

16063973623558382721422

610230488347560482412
6886200432522710497437025245

3875100811911070236915672223

240176615731293204516532610

7223205932813554549931824391
3810133524962354358525072311

1391344394647745293825

202238039155311693821255

145194512521270
3278463557357588315797625219621

10216185514821234351310614763
0000000

10216185514821234351310614763

2242344815830463712232517914788
0000000

2242344815830463712232517914788

79048215402901225110763872690655229
67036331172162554224468

67456192642787624002714312602747975
126927635231012771831003

1904939674509426410349398613960
1582516105879813218761218

12451214019811994601016999397

5016131114701472301814113345

0000000
1785955999590806234395927111509

1785955999590806234395927111509

70623163459939721330245955932
0000000

70623163459939721330245955932

21915626146089956092012
2221762598826739412108799215571

3976146825542083303423642722

3994131420121797306520002935

7132149120701671283118662487

4924142415761235218311535415

0000000
48891643101989224028572786

0000000
48891643101989224028572786

48891643101989224028572786

0000000
12643193222152210503819725882

0000000
12643193222152210503819725882

3076401554564884483935
12643193222152210503819725882

517889998097123299202614

438963268167518255692333

0000000
4961133915811544306115683300

0000000
4961133915811544306115683300

0000000
4961133915811544306115683300

0000000
4961133915811544306115683300

4961133915811544306115683300

0000000
34357110201866714821249411787023595

0000000
34357110201866714821249411787023595

34357110201866714821249411787023595
0000000

34357110201866714821249411787023595
0000000

34357110201866714821249411787023595

970859155682341981066071429363657846741
81750751322721703531392

6090218140681012141365703
0000000

6090218140681012141365703
0000000

6090218140681012141365703
0000000

6090218140681012141365703
0000000

6090218140681012141365703

285132214219
4026124732284455318722402345

24574321073203810932771272
0000000

0000000
24574321073203810932771272

0000000
24574321073203810932771272

24574321073203810932771272

154181021422395208019611054
0000000

154181021422395208019611054
2713143711311

0000000
402445102410009321019290

402445102410009321019290

0000000
430218789907800737302

430218789907800737302

682134315451337202451
0000000

682134315451337202451

0000000
878946111567556955816192350414022

878946111567556955816192350414022
4070695442692174968

19044105105146109100
0000000

0000000
19044105105146109100

19044105105146109100

1121181242457967
10477293431822558276020429541

174155605
71523313714435862354

4961798211229136312

202504027612637

83403163502654
0000000

83403163502654

519409706582640955205
5020100

318384637507563913122

81112543281725

115144232482558

2035411012113010373
1000300

942872102805252

108263819475121

39610111212914778173
1000000

206605882683679

189415447794294

2210000
113818981143451107649

898117285931626531

23870528413581118

0000000
1642033304863754016722

1642033304863754016722

0000000
144514457453744

144514457453744

5778142314975953641591096
21530322210731

94672843503361

109223328512946

107353136302448

127362133391951

51261233135243318447859

157165862545647
1000000

10583329262724

5182533282923

593452152894957
0000000

593452152894957

0000000
677255603560494476119

0000000
677255603560494476119

677255603560494476119

225353123372625
0000000

0000000
225353123372625

225353123372625

43117711519920881455
0000000

0000000
43117711519920881455

43117711519920881455

177495172834242

8611841071161199144818024063140
170622046530

1024112927958755769
0000000

1024112927958755769

25814321972686121441738
857103101410522

2911106248684674

9531266714340550571

480935863129431071

0000000
7601109210630548354

7601109210630548354

8458961050628271010805154979
0000000

8458961050628271010805154979

980194161241323172390
5073016

0000000
210603476425046

210603476425046

0000000
44656387216444219

44656387216444219

1000000
31978829011777119

171433045534559

147355245643260

5356878106122101142
0000000

0000000
5356878106122101142

5356878106122101142

18323106511824725688741601920211293
553182170668718113241

0000000
1378110920232253243423691557

0000000
1378110920232253243423691557

1107185152202104125
1378110920232253243423691557

331342849819868942357

4685278969168991128720

469169193366465195355

994846538490109856156278705261
39710515326238027185

6705457641020940903319
0000000

6705457641020940903319
0000000

6705457641020940903319

100110821
992591114113671341975621

0000000
550206314592542242336

550206314592542242336

0000000
432385816775791731284

432385816775791731284

55491650315444235526623982528
0000000

55491650315444235526623982528
1902631120817084535867738

618667234517841249

66316328840347011201228

348163234325322226239

367136222322414210190

2465126013310748591

831299852275753

343100164259392143140

24695204330331239187

381122237214371187244

352111166299411142169

0000000
527203319529520263267

0000000
527203319529520263267

527203319529520263267

1813155929593384311533041341
2047201657

0000000
120610662127218321432387869

120610662127218321432387869

5874898251181956912465
0000000

5874898251181956912465

0000000
2524139724035541334524271827

2524139724035541334524271827
0000000

307223240378400230249
2524139724035541334524271827

84637789535981027888585

7124516818841059780462

659346587681859529531

108162863821040
3920331051616241610164232407

8958081153160915181386653
0000000

8958081153160915181386653
5065096111057853785388

4612946401029

180229487435504548144

110523045773161

5361626441231

1740205831523545350541481219
4592520271320

0000000
77711461653185217022299495

77711461653185217022299495

9189031474167317761836704
0000000

9189031474167317761836704

0000000
11774288281024996879495

0000000
11774288281024996879495

11774288281024996879495

40776113192301112155
0000000

40776113192301112155
0000000

0000000
40776113192301112155

1631452433
40776113192301112155

15940701461446095

852238441534957

0000000
6225029453612267609561791085312831

6225029453612267609561791085312831
634448832684170735293

584762812051316167552471954012117
465263651951128533882

111046821918691249355574
0000000

111046821918691249355574

17191078413775046691001729
569012738948475785549937884711461

431751618313784090180035761269

73025686999578260193

714477831629342071988148947784

207292025045102856703791

105759021038322277516359

89933322519111180335238

10524665655649356298

314088590832502001961621
0000000

20472837113
314088590832502001961621

435238141926631149244

268564376022961333801374

227415394668097797429788859724788123859010613402530745660

133830373971660211051116951561042915
10100051

0
1

0
1

1

1

2

5

2

6218212311
000000

000000
6158151610

3

215410112

2316

2

5

211

6671
0000

6641

3

1

4

1

21

3111

000000
141122

000000
141122

000000
141122

141122
100000

2

4
0

4

0
1

1

12
00

2

1

1262216292014
0000000

1162214271811
0000000

0000000
1062011251411

0000000
1062011251411

2214
0000

1

24

2

000000
376542

34

221

13

12

2

1

4

1

00
31

21

1

3
0

3

521351595
0100301

1

1

1

3

7325

1

3

11

4

1

3

2

5

1

3

1

00000
12214

00000
12214

2214
0000

2214

0
1

1

00
11

11
00

11

00000
12223

00000
12223

12223
00000

1
0

1

2123

1

0000000
8720427021622829196

8720427021622829196
0000000

8720427021622829196
0000000

0000000
7419624519721227092

000000
2142724

16

81613

1111

1

00000
1211534

1

111

721

55

2

00000
12118

11

1

1

16

11

31
00

3

1

135435
020000

2124

11

11

1

23

2040103
61109884

113311

21522

31

1114

1

2

2063671
1212117212413319325

2211311

1

61

2111

1

1927216

423

310114710510014313

5

1

13131

2238131

13

2245

111

71

000000
331649

2

31545

312

241
000

41

2

1

252652
000000

5

51

1

2

26

0000000
21267837

12131

141

1115536

0000
2351

51

23

1
0

1

11

23253229233215
20021102

1711

321

22233

111

1

55747

22

167415

123311

54102725

1461421

5381411138
0000000

2

334192

8101046

128121710202
0000000

00
32

32

145291
000000

41251

1

143

0
3

3

88896111
0000000

254161

47421

213124

1

000000
1132612

1132512
000000

1132512

1
0

1

00000
82125

82125
00000

00000
82125

000
825

000
825

2

8

5

1
0

0
1

1

2
0

0
2

2

1

1

4824942223
0000000

11121
00000

00000
11121

00000
11121

00
11

1

1

0000
1111

1

1

11

211
000

211
000

000
211

0
1

1

00
21

2

1

112
000

112
000

1
0

1
0

1

00
12

00
12

2

1

0000000
2724621920

000000
432122

000
422

422
000

12

32

1
0

0
1

1

00
32

00
32

32

0000000
2316311112

21
00

00
21

1

2

310211
00000

310211
00000

2

1

7

3

31

141012
0000

0
1

1

0000
14211

42

1

1

10

0
8

8

1
0

1
0

1

0000
5166

3
0

3
0

3

0
1

1
0

1

0000
2156

14
00

1

4

0
1

1

00
42

1

2

1

2

0
2

2

22

242
000

000
242

000
242

000
242

2
0

2

00
42

2

4

0000000
917733426513525883692851302822891

81118421244319519207
917733426513525883692851302822891

2217

1581514
0000

1581514

3373251
0000000

3373251

2834154

5838
0000

5838

211
212

1

000
332

332

3221
0000

3221

17273814519
0166042

6522

11033

13

1

120159415

68113181
68214212

101

13

1394481
0000100

000
211

211

0
1

1

131223
030023

11

2

615
000

615

0
2

2

0
1

1

00
31

31

21

00
11

11

13
10

3

111
000

111

1

11
00

11

211

11214

000
312

312

0
1

1

1040040
2461142

1

122

41

0
1

1

326714203
324428407613

65954

11136102

15111271

263

428

23423
00000

23423

1
0

1

22271714266
0626031

4567

232

1022

2

1128

1

2344

21

11

12

2

1

000
715

715

2641

000
235

235

13
00

13

0
2

2

2

000000
125227

125227

2

3

12934741
0000000

12934741

1231

2

002030
810819182

5

22

712

1221112

63112

925

611121011
00000

611121011

5140021
812144102

1

2174481

12135

001
111

11

0000
2341

2341

1

3
0

3

1151
000

1151

111

00
11

11
00

00
11

11

00
14

14

0
2

2

0000000
11322321

1
10

5

4

2

13
00

13

31
01

3

22
00

22

1051415202
1561420234

2

2

23

3112

177739
000000

113

174

62

13

32

311
00

311

134
000

134

133

00000
12142

12142

000000
117433

131

1742

651022

72622
79932

628

12

1

11

0000
3542

3542

000000
2854323424

2854323424

212061811
0017585

31103

213

13

00
21

21

4411414
4001410

4

4

1

13269
12249

12

9
0

9

222
000

222

0000
21511

21511

1545
0000

415

113

178167176
0000000

0
1

0
1

1

000
431

0
3

3

00
41

41

35166176
000000

2461
0000

2461

34250
34253

3

011121
128462

115341

2

00
12

12

1113
1000

1

1

12

3433
0000

3433

5
0

5

51769311
5221110311

521

3

00
23

22

1

000
8414

8414

0260
5471

5211

3

1415
0000

1415

0000
3132

1132

2

0
1

1

0
2

2

2
0

2

000
884

884

1

1713110
0000

1713110

000
5101

5101

1015
5515

3

12

3

11151191

6321

05000
355161

310

31

26

21276
01025

1

1

1

12

2

1

1

202132
267434

52

612

0000
2212

2212

0000
1474

1474

9123261
00000

9123261

9221416289
020040

213115195

7731154

4114
0000

4114

0000
3122

3122

1514
0000

1514

31
00

31

411
000

411

34251
00000

34251

1

1

0
2

2

11
00

11

0
1

1

11
10

1

2

1

0
1

1

133
000

133

102091164
64774146716

1

2310843

21176175

33914112

81466

3646182
0021150

1

21

224

132

3

2

121
000

12

1

152332

0
6

1

5

313144
000000

213144

1

36121
03101

3

1

31

735810
00000

1
0

1

0000
2137

2137

211
201

1

4117
1001

1

2

3113

0
2

2

0101
1642

3

2

1

1

4

3329311226532
9672402

2

132

912462153

63

31

2

11611

000010
1311414

1

414

814

665140305118218214410
2000002

0
1

1

489123305118216209374
1095040065

487234

33119

250107305112216209244

49312

98112124
160172432

4

84

1

9

2

206

1

1

132

1

10

4

2

140
142

2

459127
000000

2516

25421

1162
0101

1

61

2

12119
000

7

5119

1

14

211
000

211

007000
28106154

11

1116

73382

22

116121

00000
313883

0
4

4

39883
37341

2542

2
0

2

3

221556213
0030440

175131

125242

0000000
1228833

1228833

4
0

4

22111
0000

215

26

2232
0000

232

2

000000
522123

522123

837
131

5

1

61

21219

423
103

2

12

0000000
625121416303

0
1

1

00
111

111

0
1

1

000000
112167

12

112155

16

0
6

6

221
000

221

2

2107261
000000

210726

1

1
0

1

000
543

543

3
0

3

1
0

1

10000
115103

26

1343

333

54
00

54

000
181

11

8

00
12

12

124
000

124

854973090129971263105247812124
529851676519517586692517206

4902511654565

56623761411262

481812

45714216512141

554170191210107

38920912137101

5661512371176

1519015318

6924163213101128

463119129498

63021026510110

49128321128298

113638625101414221

603325588380

5171202673366

675366101293114

324148332372

3907311668

13433903713173173

344183310644

6292772426275

4381681273683

5533112478896

42827122561856

21889856518143163

2001371041536

3902487318534

11206894725416159

5342511951179

511264553494

511233565116

53610891655136

5501593352574

32221571647

4451091799367

418145979660

64827414517387

4922649651075

62527932108986

363175264450

134844126

5252901669268

32918511210646

1340448622011181

9013724485685

72227730593110

4061041872124

36221621543

6673401814212146

40813611106739

7443091118114136

71641222198100

3491243563434

24389817134

4531497511185

62028724111122

3952572039669

46820818238153

000
183

183

13735201
1211040

11

6

3

11

42210

00000
1236161

1236161

1

000
212

212

25811121

0000000
2124740742238860749

2124740742238860749

0
3

3

1611

35878

1774460902
000540

8249
3038

211

32

9410211
00100

78121

22

417

05000
131142516

1135174

139812

00000
681081

681081

531
000

531

7225
0000

7225

1111426
0000

1111426

00010
21152

1142

2

6

153024
157244

42

2

1299124
101564

2423

2

2

23

2

10
14

1

3

11122

71

31

37

1111
0100

111

2

2

110
211

1

1

21
00

21

000
212

212

1

157134794210410
907268226215

1

2111

4

5

36

1

1119

142

1

1

1

32

1

12

2

123

21

114

42

21

12

12

13

11

1113

24

1

1

2

2

3

2

15

1

112

2

4

135

6

12

321

3

63

211

14

19

24

111

2

25

1

35

6

11

3

11

744

123

41

14

1

4

1

113

2

1

2

110

4

32

1

2

33

304
325

1

2

11114
00000

11114

1016128221322529427
0030640

1015927921321929027

2

2

8
0

8

00
31

1

1

1

1

4541392011
00244115

414

1

1

2

1

43

221

41

2

13

1

1

1

29

246
040

3

21

2

11

129111
00000

129111

8194
1141

1

752

332
330

2

14

3254884
333721

61

35

7

2113

4

61100
0173

8

69

10

22872

533351

00
21

21

3

30
34

4

3116
0000

3116

1

000000
31046124

31046124

00
92

92

11114
00000

11114

3
0

3

47521
0000

47521

412

12

000
311

311

23510
0004

33

226

59314713715217431
0043151

25

11251

49313512814815424

1

1710

3

31323
00000

31323

30120
31131

1

1

1

41
00

00
41

1

4

00
210

210

67359

12426
00000

12426

31

423241
000000

0
1

1

32201
43231

1

2

1

1

2
0

2

316
000

316

331156102314
0000000

331156102314

1003
1923

9

2

10300
12533

2

12

1

22

125715121

12
00

12

22

1

2

00
11

11

631
001

63

181211
081000

12

11

11248
00000

11

1247

35717
351147

13

3

3
0

3

2425243
0000000

2425243

415133

00
21

21

0
1

1

16321
00000

16321

71
00

71

00
13

13

2

2
0

2

314512
00000

314512

0
1

1

1

58
57

1

1510
000

1510

00000
197113

197113

0000002
118812224

111

11

751013

11

11181

1314122
00000

1314122

432

64424147

21332
00000

1

1

21221

1

0
14

14

7269

1

000
553

552

1

1

274181202

1
0

1

000000
123463

123463

1131279115
11002032

2

183762

1

2

12

122

000
1021

1021

0
1

1

952818286417
0021341

1021110
4443792

3222

44

22

2

31

55
00

1

54

1621251
012180

11

14

221

3

510510152
5755141

5

3

11

22

15769
05447

3

22

1

721
000

2

1

52

001305
289756

2

268451

41117752
430202

82

1555

845533
8585123

1

4

4

1

1

11

1261718263

22010
60342

13

88

2

1215

1536

000050
1352135

11

3427

114

11
00

1

1

42113
0000

42113

00000
13412

13412

030
133

11

2

18

00
13

13

0
1

1

1444211

19334849

1

1

11
00

11

54
00

5

4

1

421

1101115164

132115
000000

132115
000000

132115

00000
27186

1

26186

22000
22458

356

12

1

9313

6127
0000

6127

122359

3
0

3

3161

81184766530637842129
315427395312

11122
0000

11122

00
21

21

110835
07600

132

215

4

000
117

117

0000
5964

5964

4
0

4

412510

11122

3521

3319
0000

3319

1837117
90467

44

5331

32159
0000

32159

2

0000
1211024

1211024

0000000
334551245

334551245

332

00000
144177

144177

3

246121871
51212252783

14

652

1

72311

122

22

412
000

412

32121
00000

32121

0002
29161128

29161126

55271
00000

55271

0176116
4238236

4423

8

21

2127291
2124171

32

1

111
000

111

21664674
171104

1119

85267

1033112
00000

1033112

4
0

4

354251
10121713241

2

76

7426

4

12

2

221

3

15

382713
00000

382713

3

133253643
05300

27912

126152731

117642

17341

10131
0000

10131

0000000
1414825925726737736

1414825925726737736

161232
000000

161232

22422
00000

22422

2611320
0000

185314

866

0000
11071

11071

12

89211

24

2091012
0000

2091012

00
11

11

000000
333584

333584

101723
00000

101723

1361113
003021

33162

13

0000
811628

88628

3

020413
8215110

1

817

00
41

41

121867229
1322782224

311

12

4

2

2

13

2

131106121
3001000

382

7123121

31

2212
0000

2212

1

000000
6771052

6771052

1132
0000

1132

00
12

12

3
0

3

144245

1
0

1

1212
0000

1212

0000
4444

434

14

12
00

12

33536262

14
00

14

000
112

1

12

761111

26622
00000

26622

000
421

421

0000
1411

11

41

00000
11112

11112

2
0

2

216714186
10342527314126

3211

1

22352413

21

2

361

965171

13

233

4

1

11

1

1

14

1

111

12

582
00

582

000000
42311319

4237212
000000

41

234111

31

00
13

13

00
44

44

4571626492
023010

40
51

11

1000
83122

15

726

1

2

010
622

2

42

1

2091227
0000

128711

81516

00
12

12

074
286

212

0000
41411

471

71

27127

2

5431

07268182013
4106135373923

1

1

1

3

5

2

131

1

1

1

15

2

1

2

11

2

1

6

1

1

1

2

34

3

3

2

3

1

232

4

9583

1

513

3

1

416159204
000000

416159204

15231
00000

221

151

31752
00200

2

1532

21

027116252
4213317443

14212

37

21

1

221
201

2

00
21

21

00301
496318

16328

3319

31000
4169518

1

1159418

22
00

22

00
13

13

0000
1310210

1310210

12

1

101497107
310022

975

1

211

12253

31

14

1

2103
241

2

42

16235153
30238364

1

2

52

2

11

1

2

1

11

1

1
0

1

1257

4

31

1112

000
4432

4432

11

615

12
00

00
12

12

23

3
0

3

1

0
2

2

1001
2411

1

4

1

00
31

31

000030
413142

2111

2122

2

10
11

1

000
438

1

37

4

111

21114

14
04

1

0000000
15111820198

15111820198

466411

31615174
00000

3413
0000

3413

0000
11453

11453

11091
0000

11091

1150321550
11548

1

101

49261141

2

1382

823221

64484546348989418
64484846749490318

3

212

3213

4

00000
24212

24212

000000
33143518

33143518

3128
32213

5

1

411
000

411

1313

3
0

3

845370811
00000

845370811

211

6441
0000

6441

7345

13
00

1

3
0

3

1823

1114
0000

1114

3

11291
11130

6

11

50
52

2

1

00124
12124

2

1

00
16

16

111723174
9131190

2

2143

12

4

1

1

11

5111

111
000

111

127
000

123

4

5

181
000

181

1123
210

3

2

4

13

188514

1132131
0032111

1

12

2
0

2

0
2

2

401111
6454711

333

11

1137

1

113

000
231

231

11

24512217312
00164050

13411

62

21

3

111

151042

493822

13954236861

1

10202140
581244271

3621121

51111

1331

00419432
1810721421020030726

141256

1810620619019429918

0
1

1

0
2

2

52
00

52

167011583801497

0000
4774

4774

0
4

4

341183265
124131442

93311

2821512

352
000

3

52

000
642

642

2129443601095
10562425652

2

3

112615

4

11

11

119342

11

2

1

4

1

41

23

4

2

11

125

51103

31

3

3

51

2

4124

1

21
20

1

322
000

322

3

2534

0
15

15

0
1

1

383213142610
3714103193

1

22

13

1

22

612

92553

14
00

14

0
1

1

111211018234
0012031

1

102

1

418101
408101

1

241
020

211

1

00
12

12

01
31

3

2111
0000

2111

210336
19006

3

13

1

1642
0431

1

2

11

46109133
000000

46109133

00010
154736

5225

12431

177127587411436605827
0023430

0000000
3411351118

34113310
000000

34113310

0
1

1

100
1118

3

115

1152914315211
0100660

00000
31331

31331

211252
101010

13

1212

1751524
00000

1751524

0000000
12121342

12121342

1
0

1

2138
2021

117

0000
1111

1111

0
1

1

4
0

4

00000
22111

22111

1124
0010

12

112

475947935433446356
0000100

000
4410

16

32

42

311152
000000

311152

3534553313004268
0000000

3534553313004268

010
111

11

3000371
1651212172340

1514310

125711101329

21
00

21

0000
2211

2211

0000
1142

1142

0
2

2

11
00

11

302021
1672343

112

621

1211

1165966376276742
2016101

0006
1618

4

2

112

1030
1132

1

2

215
000

215

5611
040

228

33

35113
22112

111

2

0
8

8

2910541919
020285

322233

2663811

31565101567
10001103

511

15542314

11

7

11

11241

841163243639
40623152300

53263

1111373

28441203

000
1412

1412

2137324
100012

1117312

2

0
1

1

00000
832111

7319

1121

0230
8446

81

1

16

0
1

1

321

10
0

10

3

00
12

12

00
21

21

23125
23025

1

1

26191225288
206611101

1

5

2

1

111

12123

331

34

36389

4112119139
0097365

3

1

11

211

21

215

21

1

22

241528

5202
9612

141

3

11831
00000

11831

1222
0000

1222

1752
0000

0
1

1

752
722

3

0000
5458

5458

3710
3713

3

3

01020
584201

5

33

43101

15

21
29

3

5

23
00

23

10
11

1

15141

997133
364003

11

131

2

23

1

1

11

52441
00000

54

241

47
00

47

12341225197142562387
9000208

0
64

64

117341
114040

3

3

1

114
000

114

000
511

511

422
000

422

0
1

1

15515135
201000

5134

255

61

00000
794250

794250

24581452
04531150

132

1

12

2

0443
2443

1

1

0
1

1

411210

48214221
00000

48214221

000
418

418

00000
25142

2414

12

22
00

22

1111
0001

111

836181241
460161041

1622

216

0000
23712

23712

001
121

12

1801000
91721113

152112

1

71

21

11
81

7

1611
0000

161

1

00
41

41

587623301722132
29728311611144

15817451428

13217156560

41124384581
22415244475

7

43

11

7

2

16

1211

561

82545

0
2

2

10
62

1

12

3

1

31260
41261

1

1

0000000
1463444

1463444

38
00

8

3

2

120531812
530111081920

6546

2

411

3

1

3

66

5

00000
345163126

345163126

512
000

512

1012

000000
248766

248766

11

1
0

1

4631
2010

2

621

21323
00000

21323

2

3624211

11081410
6242119232910

523126

24

2110924

12841

1

10452525541

2121210155
250351

62331

110473

1424

13
00

3

1

7226
0000

425

321

0000
53102

53102

11
00

11

21

000
132

132

122962

52731429
0000400

0
2

2

1
0

1

21121
00000

221

11

23
00

23

6
0

2

4

0
4

4

2
0

2

41

1131
0000

1131

0000
1221

1221

23

211595

0000
210510

210510

241431
000000

241431

2

2471132
244150

241

14

1

513245

0000000
586716117

242

1431104

21491

11

811

2

251121351
2492700

1

1

265

12
00

2

1

320
354

4

3

000
122

122

4584532
0000000

4584532

11
10

1

34313

115
00

6

19

2
0

2
0

2

0
6

6

00
91

91

34

0430
1442

112

5512232
00000

5512232

6656434621024
19252091071453156

81910321511069

4414252321063

21111
00010

21

11

000
121

121

63
00

63

2
0

2

422
000

422

121
133

1

2

1732531

11

0000
83318

83318

22142421484027
4042111

42654
10212

322

242

0
4

4

2003305
423615524

219310

223329

125682
000210

1

1

4372

21

12
00

12

38
02

2

16

275142
112112

4

3

1

3

2

15

413
000

413

12
01

1

1

1
0

1

2641
2621

2

5

10001
26311

121

61

11
00

11

588246
00000

588246
00000

588246

3215127355
321297105

10

9

3

5

3

1

11
00

11

002001
15171211

121

51510

2995154
000000

2995154

3181

0
3

3

1164530
25749246

1

1

411

1310

6

11
00

11

51
00

51

2

0114411140
1420113899917359

00000
13231

13231

00000
145101

145101

475532

82416202818
164558

2

1123311

2

21

462

267767

1

310645
321696

1

151

42

00000
31464

31464

113

166815

1824125418

27151611145
311191917147

1421

42

222

101150
1326122

222

112

332

2412

2120000
15617113315245

7010164
10545239

159

31

138

10

1517

12

15361
01231

1413

0000
2115

2115

21
00

21

150
353

3

2

70910
811031

11

112

00
32

3

2

01
31

3

000
211

211

00000
11131

1131

1

1117
0000

1117

0
3

3

2110612
01739

11

133

2

0000
1164

1164

001
511

51

97513
0000

97513

0
4

4

51
00

51

000
531

531

41

000
112

112

00
11

11

62145
51101

2

2

122

1

2

11331
00000

3

1131

1118471
0013451

32

1

2

1

111148
11528

6

2

6231
3101

3

1

3

26
00

26

41152

1201217311
18143234743

6141617412

42

6

01
11

1

18
10

1

7

00002
19219

19211

6

29

0
4

4

212
000

21

2

00
13

00
13

13

123

01000
18213

4

3

1321

00
13

13

00001
1011641

9544

162

0371046171
77781129821114864

00
32

32

1217

2
0

2

3

512
622

1

1

12112
00000

12112

13433442
10101000

23

41

13241

1549412
00000

15112

394

3111
0000

3111

131
141

1

0012300
25392621

11

5412

122321

17

030011
133412

1

1

3

31

211

1116110
3316142

22

23

000001
211431

21

13

4

3321
3421

1

0
5

5

3

0000
5421

5421

11612240
01211219

4

13

13

14

1

9

1014875157
37840110

3

34

1

1

13

1

7

2

1

1

2

1

2

3
0

3

00000
21153

2

11

1

2

1

13

2736251
1000001

2525

3

1231

128

0000000
3271010171

3271010171

11546141
000000

11546141

361
261

1

242
282

4

0400400
4441642

4

11

3242

00
45

45

0000
31213

31213

3333
2022

1

11

3

2116131196
6118181197

3

12

23

1

3

2

33224
00000

33224

322

2321
2300

21

1

31
30

1

7311
0000

7311

31

40
122

4

42

1

423140
646182

22332

1

1
0

1

111

41243121
000000

000000
174391

000000
174381

174381

1
0

1

33
00

33
00

33

0
5

0
5

5

25837
00000

25837

0
1

1

12
00

12

1412554
0211352

12

2

12

0
1

1

11

10213
41513

31

3

120600
324742

1

143

112

60
84

2

4

225
000

2

25

00000
1213611

1213611

4233515
00000

26217

217148

1167121
1067111

1

1

134768515945361063109
234129160

14
00

14

2
1

1

213

2121
2001

1

2

000000
2434121

21

31

13411

4050
4182

2

1

2

1

112
00

112

00000
352113191

352113191

15

000000
118540393341661

118540393341661

0000
15119

15119

1

11
00

1

1

2
0

2

226
000

226

0
5

5

192618
0000

192618

0000
5124

5124

2724
41271373

186938

2114231

72413264
50413254

21

1

1

14101310
0000

14101310

366
000

366

42

00
52

52

0
4

4

000
512

512

00000
612134

2

143

2191

21

10030
184418

18

1

849

164
034

3

1

11
00

11

21
00

21

00000
11211111

11211111

2
0

2

00
12

12

0000
4311

4311

000
134

134

33
00

33

11
00

11

577532240
180500020

13319

916

61

422

1111

633

1
0

1

00000
31442

31442

00
11

11

0
3

3

230813421
0143331

27

3310

93714

48

2

0
1

1

17
00

17

000
432

432

11
01

1

14434
0000

14434

21
00

1

2

4004
10125

3

1

2

1

1

2

00
41

41

0000
4131

41

13

0
4

4

5
0

5

44105
44103

2

3
0

3

41568171
000000

1155816

3111

1
0

1

114322
000000

114322

060000
361584

1

1

121

7

2123

472
000

472

5
7

2

0000000
37645817

37615317

35

0
2

2

001006
1261612

2214

1

4

2

1

31

43
00

43

233
033

2

112

1
0

1

0000
17104

17104

010
341

1

321

13
00

13

000
214

214

3

11
10

1

1
0

1

1
0

1

2
0

2

212223
27123

5

1

1

0
1

1

152714
0000

152714

0
1

1

3212
2211

1

1

0350
2695

2345

258
000

258

000000
912142

912142

0000000
5452627409110

2

5432627409110

221
000

221

031220427
3313201127

317

182571126

00
21

21

441331
441330

1

1

12624
8203

21

2411

1

000
151

151

0000307
81128110

41242

41111

21000100
31821101

6

22

1

1

1

2
0

2

121
000

121

2246

136904
1112221306

81612302

1091120
1330

24616

7224

85124

000000
1123431

1123431

000011
177554

211

1

2

1

16131

16

33
00

33

010
433

422

1

0000
7234

22

232

5

2
0

2

4935

011222
114524

31

22

1

0
1

1

0233071
18391121

15313

2

11

2

341213
141200

12

21

121613

333

4

2
0

2

2

1
0

1
0

0
1

1

1532
1530

2

322

1003010
26541034

3

2512

11104

1

1725925
0000

1725925

000000
127112

127112

10000
161452

61

9125

2

1

12

000100
213111

1

211

21

15114130
15915261

813

11

595203

114

3123
0000

3123

00000
25541

25541
00000

25541

3

1

5243

1316119

000000
31121061

31121061

1053
5189

9

1

2

2

2

2

3

2

21

2

1

2

1

1

1

3

1

1

2

52

00
11

11

11

0
6

4

2

15
00

15

00100
17514

1714

31

1

0000
35111

35111

111151
000000

111151

00000
21331

131

23

1711

182121
00070

1

41

182

00
23

23

000
112

112

31
00

31

1537517
000000

1537517

1000031
3231342

22

1

11

1311

1612910186
16114546

3

7

15

2

12

121

1001020
1612721921815419824

1

21

1

1212721921615319624

3

341
000

341

00000
63615951

63615951
00000

63615951

00
62

62

0
1

1

6514
000

6514

1

0000
42513

42513

13
10

3

102343220174
1000200

11
00

11

11
01

1

0
5

5

3111
3121

1

41

11
00

11

10315
0000

10315

000
411

411

00
223

223

1
0

1

1111673
141620

4

5

2

1

2

1

4131

111

0
1

1

1

3123202166
2108303

3

1812136

1

1

3

1

1

1

1

1

1

1
0

1

333

13

00
12

12

101457
7413

3821

223

01222415112
233212697818755

01300
21351

3

221

1
0

1

00
23

23

00000
12322

12322

1
0

1

31227
00000

3223

14

8281
0000

8281

95
00

95

0000
6151

6151

212
000

212

00
15

15

000004
121435

212

1411

11

00000
13441

13441

000
313

313

224
000

224

000
421

421

0000
1312

1312

1
0

1

32
00

32

0110
2111

21

61366

0
2

2

00000
14112

14112

2035
4335

3

2

12111
00000

1211

1

132
001

11

21

7311
0000

211

71

11
00

11

0
1

1

000
241

241

5
0

5

0
3

3

112
000

112

0
2

2

72
00

72

000
516

516

000000
462133

462133

11

0
1

1

45
00

45

00
12

12

13
00

13

000
141

141

0000
1232

1232

0000
2283

2283

213472
00000

213472

1
0

1

111
000

111

000
455

455

000000
151611

151611

0
4

4

000
1104

1104

15
00

15
00

12

3

31
00

31

0000
1123

1123

265604823537305
481126922761

2080246
39171471

1

1

1

61

3

3

1

4

1

221

1

11

1

25

21

2

42

42

1

1

13
01

12

764

101211312992
17032221320166

71

1

1

34

2

11

31

2

41411

23

55

3

3

1

3

28

11

21

5321

4

2

1

6

1

1

1

3

2

2

3

113

36

52

1513

6

1

1

22

131

215

13

000
231

231

392642332404607
381711681652964

31110

17762731393

132115

1

0
7

7

413316

102216401

2010
3251

1

241

0
4

4

1000
810516

61

1612

452

1

4
0

4

1

00010
22011121

213

2181918

01
12

1

1

0000
2142

2142

210121112
00000

210121112

961317
951217

1

1

61131

110111

5432
0000

5432

0000
1636

1636

414

1312131262
667332

16582

211

1

4

217738

233131434

0
4

4

71
00

71

00000
11121

11121

335
000

335

01
21

2

1
0

1

0000
2081116

2081116

1461
0000

1461

1

121
020

11

41111113
3110043

114

13

0
2

2

31

1

11142
01000

4

112

003330
1520347

10

3

1

5

7

1

13

532343144120
1377256

33113

111243

3373116

19184102

1961414

1122212

713421515
1300000

6104413

1

212

1

117431
10100

72

3711

21

1113
0000

1113

000
531

531

00000
41224

41224

11

120
123

3

42164
30002

12162

0030103
28124145

12

1

12

18632

11

012
212

2

34

1513

2

222
122

1

1
0

1

000
11314

11314

22373
00000

22373

22
00

22

0200000
18169045566651

0
4

4

0000000
1781114161

1781114161

12
00

12

27100
216122

82

12

1

1000000
711857109

2332

4118124

2383

12

1

0
4

4

0
5

5

4725
0000

4725

3124
0000

3124

0000
82111

22

6219

412411113
11147110

1

1143

1

00
31

31

3115
000

3115

1111
0000

1111

292416
062305

21

2111

6351
0000

6351

81
00

81

01
71

7

0
3

3

0
1

1

1
0

1

1
0

1

1219
0000

1219
0000

1219

73

243355451
00000

243355451

32
00

32

22

4326917287
654818285612

123

1

1

4

21

12

136

234

1

1652135

12537
0217

422

3

1

1

12

1

23
00

23

72840821529740132
86082842754290750

21

1211752751594

11211110432

7864440847

121961031192205

129
000

129

1113
0000

111

3

792
000

61

731

0
1

1

4541183
000421

424

11

2

131

332

1

15113121710

466

14486272257259166178
0000000

0000000
13378266254253162173

98641314
000000

6
0

0
2

2

0
4

4

00
22

22
00

22

1
0

0
1

1

0
2

0
2

2

0
1

0
1

1

119
000

0
1

1

02
14

1

11

0
1

1

4
0

4

765352
000000

1

42
00

4

2

000000
324232

2

1

1

21

1

13

12

1

000
212

1

1

12

1
0

0
1

1

0000000
11473245242240140152

11473245242240140152
0000000

0000000
8364243241224140140

8364243241224140140

29911612

22
00

2

2

105136997
0000000

000000
322243

0
1

1

1
0

1

1
0

1

32224
00000

322

4

2

51
00

00
51

5

1

1
0

0
1

1

1

3

521131
000000

2
0

2

0
1

1

1
0

1

0
1

1

1
0

1

1

0
1

1

0
2

2

0
3

3

3

000000
152311

000000
152311

1

33

1

2

2

1

12
00

2
0

2

0
1

1

11
00

11
00

11

71515
00000

00
15

15
00

1

5
0

5

000
715

715
000

0
2

2

00
21

2

1

000
314

2

1

2

1

2

000000
285213

000000
285213

2813
0000

0
1

1

0
8

8

3

0
2

2

0
2

2

5
0

5
0

5

0
1

0
1

0
1

1

0000000
447541597267

0000000
447541597267

0000000
447541597267

42454196265
002000

2326

00
11

1

1

34

22091
1691

15

17

27

83

3

5

00017
3244263

4

26

1342

117

4180

1

3

2

219

1

5

1

1

2

0
5

5

00
41

41

0
1

1

23512
00000

1
0

1

1

0000
1322

11

1

1

3

1

0
3

3

1

54

5

1

441
000

3

1

1
0

1

1

11

1

3

3

2

1

14101515237
0000000

0000000
1471414237

114812161
0000000

11489161
0000000

48312
0000

48312

0
1

1

1
0

1

44
00

44

0
2

2

0
1

1

3
0

0
3

3

000000
336276

342
000

000
342

342

0000
3276

1
0

1

0
1

1

2
0

2

1
0

1

0
3

3

2
0

2

0
3

3

5
0

5

311
000

311
000

000
311

00
11

1

1

3
0

3

2

1057404565443541615228
0000040

82125414911014515155
0000000

00000
11531

11531
00000

00000
11531

11531

82125314810514215154
0000000

1000000
82125314810514215154

0
2

2

11

000000
131731

131731

1385
0000

1385

0
2

2

22
00

22

5101301
109315311

1121

1723

24

1211

11

0003100
68106643

15212

2531

211

13223

11

000
141

141

2
0

2

000
333

333

322
000

322

1000
7712

252

421

010100
822114

5112

32

11

210
214

2

2

4121

9667171701037
0023620

11

212

659676357945

17147

321

45427
00000

4

45423

4033201
7353473

12

1

1

1

1

36

1

2

112
000

112

0000
2244

2244

1

7611583891819
000000

7611583891819

236150416333396460173
0000000

654422521021225452
0000000

654422521021225452
0000000

000
161

161

5129711

105110638613713
0385853

15

2282304472

433

13211832

311

3

116

3316214

42

29623
00000

2

4622

51

000001
12284439507

23553

10284134453

1342
0000

1342

3061112104

1040070
5327592544516

2

52

4412124

13

427678538258

112

0000000
23283021403622

0000000
13521913162

113

1342099162
4010001

4451111

1

117712

21

333

1

0000003
818912231918

231
000

2

1

2

1

17317
00000

3114

1423

000000
117523

12

5212

12111

146
000

146

1002000
4106411104

5

5171

21441

11013

00000
25412

5411
0000

11

21

411

21
00

21

1487816110214417099
0000100

00000
105215

11
00

11

0000
10515

10515

136631479113116185
00234161

0000000
14121132164

14121132164

0000
3767

3767

24161210111522
81052557

413142

125485613

14914912203
0000000

14914912203

0001101
39101817292724

229656155

1011041555

7277713

541

111030106128
1100011

74114265

35196452

13133122404011
1000002

98201619275

3511621134

0000000
18471201111

18471201111

1251461089
0000000

00000
28133

28133

0000000
105651056

105651056

2

1

2

1

02000
67534

2
0

2

1

5

01
61

6

53
00

3

5

13

1

2653522691

1

5327111

1

1

16323
00000

16323

2

1

4031317702642572662618730
0000000

184171108
0000000

184171108
0000000

184171108
0010000

0
1

1

114
000

114

0
1

1

000
211

1

21

81593

0
1

1

0000000
4031217622602402651618722

3623815292412312481218377
0000000

0000000
3623815292412312481218377

18210030099
3620615292412312471218346

3

1

1

53

1211

1

737127

3588315092412272471218209

11
00

1

1

3131
00

1

2727

34

4074233199174345
0000000

4074233199174345
11000000

000
331

11

23

00
333

1

1

322

0
28

28

2342227

000
29415

151

2

1

110

4

34

6

1

0
2

2

0
3

3

0
4

4

1
0

1

27

1
0

1

1

5000000
7323183104217

2

2

825

1813

2

3556127

3

6

72

4

34188

21

11

112

518854

6

123

22

1

77

24

4

35

39

6241

2

93

319

13312

11

00
101

1

10

1110000
293819296368

55

1018

30110415

17361131

60317642219

131445

1

2
0

2

0
11

11

0
19

19

0
1

1

0
2

2

2

0
2

2

0
1

1

0
1

1

10

1

1

1

3

1

2
0

2
0

2

95340589592594827192
0000000

000
111

000
111

111

94340588592593827192
0000000

0000000
17679152739

33215
00000

133

211

1

2

11647122731
0000000

121121

479264

3

1

12

5424

00
21

21

122
000

122

0000000
75332576583575800152

00
11

1

1

1001301
1225691511

2

17

1

5

1

21

13

1112

2

3

1

21

11

1

123

1

2

2

2

62329571575566785141
0013630

1

1

2015

2131

1817434131031648955

11

1

1

29

1

1

111

1

3

16511

21

5

1

1

5

41

24

1

3213374

11

13

1

111

2

32

165

12

337112

2

44

212

1

514119520517924829

21

11

11

4121

3

22

1

1

13

112

1225195

422

23

6

3131

3

0
2

2

22531
00000

1221
0000

1221

151
000

151

2

2
0

2
0

2

11

1

4112149105
0000000

4112149105
0000000

0
1

0
1

0
1

1

00
51

00
51

00
51

51

317148105
0000000

21756101
0000000

0
1

1

15
00

15

0
1

1

125610
00200

1

1

13610

1924
0000

1003
1914

11

9

0
1

1

0
1

0
1

1

2

134172302240227350155
211229712

30232433272227

68123101316

21114617153226

10154516232111

32871151389818930

33274831456633

2

1

4314
000

000
4314

000
4314

000
4314

1

2

1

6
0

6

4

41

2

214

00
11

00
11

1

1

2

47254242471940
0000000

3282210191713
0000000

3282210191713
0000000

00000
53211

53211
00000

1

1

1

131

11

11

214334
000000

000000
214334

13

1

1

1

1

42

1

11

00000
52612

11
00

1

1

0
1

1

0
1

1

00
14

14

00
32

1

1

2

1

2
0

2

000
137

00
31

31

00
16

6

1

111
000

1
0

1

1
0

1

1
0

1

1
0

0
1

1

57351
00000

00000
57351

634

1

1

4

1

1

0000000
13281332

0
1

1

4

1
0

1

1

0000000
8271331

6

1

3

1

5

1

1

1

2

1

1

1

1

5111
0000

5111
0000

1

2

31

1

121
000

000
121

121
000

0
1

1

0
1

1

00
11

1

1

0000000
1417203126226

1217202923224
0000000

3

000000
2139618

1
0

1

0000
2321

221

3

0
5

5

21

11
00

1

1

000
726

3

45

2

1

0
5

3

2

2

0
3

3
0

3

000000
9243910

0
2

1

1

1413
1000

43

1

1
0

1

1
0

1

0
1

1

21
00

21

2122
0000

1

11

1

11

1

00
11

1

1

1

00
12

12

2

1

00
21

21

0
1

1

00
31

31
00

1

3

1
0

1
0

1

1
0

0
1

1

144
000

1
0

1

00
44

44

14
00

00
14

4

1

1035212
000000

1035212
000000

1035212

2232
0000

1
0

0
1

1

2
0

2
0

2

231
000

00
21

11

1

2
0

2

1
0

1

1

2
0

2

1

2

2

323

1

3

2

2

1

0000000
8103912138

000000
293232

000000
293232

5223
0000

0
1

1

513
000

513

2
0

2

2412
0000

24
00

24

00
12

1

2

6114635
0000000

6114635
0000000

41144
00000

0
1

1

00
24

4

1

1

000
214

1

1

1

3

1

0000
2631

0
1

1

12
02

1

3

00
21

1

2

1
0

1

0
1

1

22471
00000

22471
00000

00000
22471

00000
22471

22471

1

13

28673913146
0000000

286722764
0000000

286722764
0000000

1111
0000

1111
0000

11

1

1

276712753
2000000

0000
136311

1

51

862

1

1
0

1

914

1
0

1

2111231
0010000

11

1

2

3

1

1

32
00

32

17682
00000

000
768

768
000

000
768

746

22

0
2

0
2

2

0
1

0
1

0
1

1

2

1

1

3225738124

13911

13

3

1

14596157272888103543335333467377298445644143738211877207
121389426228645003624776549167792172177120

137619130510919951596245
0000000

5070100
137619130510919951596245

0000000
2611814617312517354

2611814617312517354
0000000

0000000
2611814617312517354

2611814617312517354

10650111529188691423191
0000000

0000000
10650111529188691423191

10650111529188691423191
0000000

10650111529188691423191

13965224129351108610381152504803
116618619613520232

1001100
658194356094763454860902610

0000000
3462193141121151106

3462193141121151106
0000000

0000000
3462193141121151106

3462193141121151106

623188154164621442659392504
48263525164

0000000
1522748416897658371276

1522748416897658371276
3749186122138192785

122

7137169113241159166

1715637936230137965

711311816228

19319479759132

467159945493897363650861224
01210350497115

59170463428463605180
0000000

59170463428463605180

7369392778859
0000000

7369392778859

1417521933174816072464340
0022000

57222680636564783157

845301251111010431681183

2606291957157914401858630
81411567447717

127181452330347399251

73312919730706958195

52122471452343424167

36264428505348
0000000

0000000
36264428505348

0000000
36264428505348

0364576
36264428505348

1329491112

125105171412

41212106119

747513109

11101211329
0000000

11101211329
0000000

11101211329

3812422325217327671
4040020

2610719118513522836

8172867384635

31316124215
652306468635835546485972033

89132358279306342184
1708682

0000000
11294330534335

11294330534335

12117828305410
0000000

12117828305410
0000000

12117828305410

382992818710973
0000000

382992818710973
0052010

23164234526731

15134545354142

0000000
275614513213012864

0000000
275614513213012864

275614513213012864

560291964895544515482341844
0000000

560291964895544515482341844
3818351944235959190

2165188162141197112
0000000

2165188162141197112

0091211251
2279882387191917892783722

52191367278286334147

9129410317677951187345

845039808626971237229

55270674596599973178
0000000

55270674596599973178

0000000
633858137285981129291

633858137285981129291

0000000
51319642578530752161

51319642578530752161

0000000
1057091266111911381809290

1057091266111911381809290

0000000
1356579999389651512263

0000000
1356579999389651512263

1356579999389651512263
0000000

0000000
1356579999389651512263

0000000
1356579999389651512263

1356579999389651512263

4223202934873240153382005559918651
2175935505557

79220875762695862619
3923189114570637702359445190617735

101642481024883087935116434103
0000000

101642481024883087935116434103
11301419990126142

0000000
2283279176194256152

2283279176194256152

0000000
8149848577039

8149848577039

0000000
206417211184115137

206417211184115137

1157391353126210581916310
0000000

1157391353126210581916310

0000000
40114401284269328194

40114401284269328194

0000000
81364855576650927314

81364855576650927314

714269928237491159325
0000000

714269928237491159325

371153831082793273439101023
2000000

1685691336122011711597534

2019691772157315632313489

0000000
8387250455678

8387250455678

2839138886414966
0000000

2839138886414966

1412511033744737883756
1455250137124125140

5235361487466

2541184153147230137

27401229895122105

1687480818185

3123542342432

172311043717244

15711855748855

19428775636792

0000000
26184734560464843124

26184734560464843124

3569252187191298301
51644344869100

20271218572133123

10268768719678

39295620507549607142
0000000

39295620507549607142

92545421176997859271127376275
0000000

92545421176997859271127376275
32124383383351488443

3236310154240199286
317165843443551346146491844

59223513456428508340

7340412839618811387347

1539952238198019122555871

1671208147151184146
0000000

1671208147151184146

19090622902222198124741236
0000000

19090622902222198124741236

15651616641285112717261161
0000000

15651616641285112717261161

198119227282063208630521314
0000000

198119227282063208630521314

0000000
1675152134114164131

1675152134114164131

5268460466561
12206435154931257411975178974836

14155947458947
0000000

14155947458947
0000000

14155947458947

062721244328
456223548774038361358301693

0000000
2488153121108147122

2488153121108147122

74251385308302535215
0000000

74251385308302535215

0000000
940120609712987

940120609712987

0000000
235317212710822661

235317212710822661

0000000
653907726416551060274

653907726416551060274

26114073248276023193690906
3132021223128

10044510919008381190359

1589492137183914592469519

1252183157161195110
74541591047384298271119133035

41285738563543750115
0000000

41285738563543750115

04411215317
26113259232248265171

127111011311012573

143810810712310991

0000000
64290834651635908280

64290834651635908280

0000000
3275399333332463283

3275399333332463283

1984203144122239115
0000000

1984203144122239115

783288736776781068250
26251481119

56277722582561892137

20451268110916594

32145288273241289130
0000000

32145288273241289130

1537961885142414012147448
0000000

1177431448119111731818304

3653437233228329144

0000000
2013344432241355377

2013344432241355377

18391341009316384
1227581986157415482250438

30170691520507694137

142310085849856

6052610618698641295161

0062030
594229107726851031244

51390796682604936180

83210888819264

2501041088412120
876781471130712641755374

5750010919929501349199

28128276207230285155

0000000
683346673216273606887671902

6135744385150
683346673216273606887671902

0000000
24312502677239222463156616

24312502677239222463156616

0000000
31109402322295454102

31109402322295454102

8150311489529071284249
0000000

8150311489529071284249

0000000
19012712408192420063100475

19012712408192420063100475

0000000
132320629639576722410

132320629639576722410

0000000
21197439677437

0000000
21197439677437

0000000
21197439677437

0000000
21197439677437

21197439677437

44189470357274561109
0000000

0000000
44189470357274561109

44189470357274561109
0000000

44189470357274561109
0000000

44189470357274561109

0000121
23311572423202018653003713

0000000
1498531672141413172212349

0392752
1498531672141413172212349

0000000
11215133427037

11215133427037

1237671510129311962005257
0000000

1237671510129311962005257

1562102867213253
0000000

1562102867213253

56265636502455672277
0000000

56265636502455672277
0000000

56265636502455672277
0000000

56265636502455672277

0101002
28391151049211786

0000000
8205154495124

8205154495124
0000000

8205154495124

0200000
20186449436660

92392853417
0000000

92392853417

11142521383243
0012300

51245191724

622014161519

171161820128
0000000

0000000
171161820128

0000000
171161820128

171161820128
0000000

171161820128
0000000

171161820128

548194909414599
0000000

548194909414599
0000000

548194909414599
0016002

0000000
35313527395550

01310054
35313527395550

1224149192320

236188202726

0000000
19505857559047

19505857559047
0000000

19505857559047

2914331929721331747

0000000
27213993082261023573493794

42102924272536
0000000

0000000
42102924272536

25513914516
0000000

25513914516
0000000

25513914516

0000000
1751615132020

1751615132020
0000000

1751615132020

23013893053258623303468758
0000000

0000000
23013893053258623303468758

23013893053258623303468758
0000200

12810211814
20375248356332

31421611218

5711263174

8101410176

21013523001253822933405726
4183526236743

6294727413119

237513816613219483

5649810688707951066173

46230464467406601136

20571281068712475

38225529487424733141

1722059238938558956

0000000
71352437388943805104380422032

71352437388943805104380422032
3080002

65351469191228203456177510573
1131161115

1121202
5909755886179124955479732

20902153035298281085387
5902751857177924845459702

20029946511524

438555718727061354

8319263651731

3378961234

1372140117358539671039

68442126127

2412

1741091749

619585514729823171

634023371812153

1

2703267899866

21

6232616181724

101971372

57285252928118

2061623688

2565306626

1171810108

86182649454095

912249345225162

1184112618727

1169142019728

942123122429

18154148405343

4271366610

12725

34351224511

0000000
6327119228

6327119228

179392595567525839277
00020124

06614112
2492619183524

815626

11734127

52898129

5161415111023
0000000

5161415111023

7294516223531
0000000

7294516223531

2918331628921
0000000

2918331628921

0000000
1373619281723

1373619281723

226857785912046
1235661

15512551326931

6152922214514

0000000
6123134938833355188

359115913816822840

2614019025016532348

18143514265017
0000000

18143514265017

1613148121616
314160167208221187278

38132630252333
17762799610377136

3211

421

8256745

122151

111217

2512794623

113356314

1378372

21

1

315332

24432

2853522324

611141

3731324

81179418

797324

2211

11241

2331163

121857410410694126
2715925131245

2821109

6314268

3126345

331

1111

31423

443

11465

49322

3211

5211

422544

36544

1112

22314

141212

1991

3132673

923142426247

13746715

2522

241621214115

98104164177155129193
0000000

25374712
9698145170140117180

10203739101724

84151531117

8141719111911

6855739011263116

0000000
26197151213

26197151213

1000002
34458971597278

0000000
1271222201540

1271222201540

0000000
8213225254320

8213225254320

13174524141416
0000000

13174524141416

2567211099193112209
0000000

2567211099193112209
0000000

27104723737
2567211099193112209

295131011925

5923716241829

53144730483657

58102725582446

30101211291815

329896184614461449190011236
0000000

329896184614461449190011236
0000000

0000000
265583901249742

265583901249742

51274043595054
303841176313561325180311194

2819574943039662269

24132120236

4

1071619242724

2413918141220

13103325361714

58858221

194232991519

2418227192024

1324142128446

5746976070264787610867

2720306252932

8262013131829

22162617272429

000000
12131561712

000000
12131561712

12131561712
000000

12131561712

0000000
1815241037231748480

1815241037231748480
0000000

1815241037231748480
0000000

1815241037231748480
0000000

0000000
1815241037231748480

1815241037231748480

867168025261995205226611653
0003000

7379145104170132109
0000000

7379145104170132109
0000000

7379145104170132109
12151020306

23121713341733
0000000

23121713341733

31211512131936
0000000

31211512131936

0000000
184498691036634

184498691036634

175298773596626894347
0000000

332113361
175298773596626894347

0000000
52357029515659

1010001
52357029515659

23153615172225

28203314343433

120260682554572832287
0051205

0000000
582320096154180144

582320096154180144

62237477457416652138
0000000

62237477457416652138

619130316081292125616351197
0000000

619130316081292125616351197
0000000

4242117131617
619130316081292125616351197

14258351667753
0000000

14258351667753

0000000
99255496441354660261

99255496441354660261

651371091186897103
5029991008783823882866

331079254476345

86536832405358

141294350337391358382

37211163106101135108

399610674788560

37575837583541

64446225405669

0000000
273278898664842889249

273278898664842889249
0040000

0000400
257271861622808861213

8422577855567079289
0011000

1040100
7921976454365478381

5321174152464275749

2581919112632

5613111698
0000000

5613111698

1541511232813
0000000

1541511232813

0013000
15842685611141111

299151921936
1000041

837184319

13551614

7131122

15613311712
15613311713

1

0010200
28112921191914

73912846

1931086104

2591354

8616101060648
0000000

8616101060648

1673342342836
0000000

1673342342836
0000200

73224121315
0000000

73224121315

0000000
943118201521

943118201521

110505827861799970458
0002040

72310532555512690158
0000000

0000000
72310532555512690158

0000000
72310532555512690158

0000000
72310532555512690158

72310532555512690158

0000000
3297212198184203248

0000000
3297212198184203248

3297212198184203248
0000000

3297212198184203248
0000000

3297212198184203248

698831061037352
0000000

0000000
698831061037352

698831061037352
0000000

0000000
698831061037352

698831061037352

0000000
162316811810117169

162316811810117169
00712871

1477354456642
0000000

1477354456642
0000000

1477354456642

2168852489826
0000000

2168852489826
0000000

2168852489826

11132235163228
0000000

11132235163228
0000000

0000000
11132235163228

11132235163228
0000000

11132235163228
0000000

11132235163228

15123828252510
0000000

15123828252510
0000000

15123828252510

0000000
27414762009198818022881579

27414762009198818022881579
0000000

1020001
27414762009198818022881579

0001102
14373294910078951386341

0000000
825787558357171178190

825787558357171178190

61154194171177208149
0000000

61154194171177208149

13074410589819071495237
0000000

13074410589819071495237
0000000

13074410589819071495237

22333222202112
0000000

22333222202112
0000000

22333222202112
0000000

0000000
22333222202112

0000000
22333222202112

22333222202112

3474143151130133101
7752418276992064713591679452016142

33120603628318529544934772
0000000

0000000
33120603628318529544934772

0000000
33120603628318529544934772

33120603628318529544934772

0000000
443921292347393256829628473998393

5211215998121206162
443921292347393256829628473998393

0000000
83426609676601871188

83426609676601871188
0000000

83426609676601871188

0000000
8905056748073886543105432006

8905056748073886543105432006
119715110011529611583329

25115722542259023273450564

1625948229028001241318

13566699310099311507321

17111831585138211862098358

52326438353338664116

4176100778914669
1210420674336724614996212057

0000000
1638531400132911001835336

1638531400132911001835336

0000000
78245409465388643204

78245409465388643204

0000000
3766401373118510611621333

3766401373118510611621333

23286953646149
552239241513668351153761115

1908281540116812441796339

33915362542244722033519727

107408643552590875225
220411492190581768216214261583980

0000000
28013282102194917423007415

28013282102194917423007415

21712041934164416132625482
7684410735269036342101081646

13393436337441

1539961894162015892606277

1308391360131112311971222

75315550574457788155

145123120328

735428849627451133200

93470684725654879261

10495346896182787540121681694
35420143159292126774301507

1517841762166014892291238

23510361361128911391977440

1849751644151713172273304

12553710358919181326205

8296647494855
270716568285422622423923382466280

0000000
27215092784262624613762647

0000000
27215092784262624613762647

27215092784262624613762647

0000000
6694636798673166474103281513

0000000
6694636798673166474103281513

6694636798673166474103281513

175810394177061623514939241084065
1510115616520321753

38522953744333031105007813
13237745136531233311436182132972

9385450990990038326132062159

611513153610
0000000

611513153610

0000000
27516592505232120913742658

27516592505232120913742658

0000000
1398781387140311941900372

1398781387140311941900372

0000000
24118332868258525323808596

0000000
24118332868258525323808596

0000000
24118332868258525323808596

24118332868258525323808596

0000000
3183737202830

3183737202830
0000000

3183737202830

21862910659138081107544
0000000

21862910659138081107544
0000000

21862910659138081107544
0100000

0000000
2722840833830747874

0000000
2722840833830747874

2722840833830747874

191400657575501629470
2674947

40205298280218309204
10225333196525

8506558535359

7451858446725

64110392696535

9475939335960

28598249523062
0000000

20294636181031

8303613342031

22176773647263
00118793

672222212922

892320212125

811123151313

116191418179
99113203169158214134

53142323363342

16488159627742

31156796

2372223

2

5131830123616

9264035214016

0000000
3619241331530948994

2118434426127140159
0000000

2118434426127140159
0000000

0000000
2118434426127140159

2118434426127140159
0000000

2118434426127140159

1586954388835
0000000

0000000
1586954388835

1586954388835
0000000

0000000
1586954388835

1586954388835

0000000
17326520253543

17326520253543
0000000

0000000
17326520253543

17326520253543
0000000

0000000
17326520253543

17326520253543

19549774175291577214757218515619
422516342623

9035093878480097344105142412
0627397931237

776443977137008642391692034
7528871486320

787130544340
642336760785486502770341771

0000000
2524268856549160991

2524268856549160991

3152023
107555223254312321468

17393461687133

10575578559166

603967170135105318

17625843545248

1104202
1799651907159815682147380

3717329131829543883

66452805663644919154

75339811613627790141

1999201796173614412211470
0011191776

3610716118513621488

92555122611019361473165

3214421625121728766

39114182180135230145

42256479453423633121
0000000

42256479453423633121

1913025625718326688
0000000

1913025625718326688

0000000
64291658593555804113

64291658593555804113

0000000
12710201547145113482072243

12710201547145113482072243
0000000

12710201547145113482072243

0000000
1275929989048281222371

1275929989048281222371
0000000

4281920424022
1275929989048281222371

47179278288272373150

76385701596514809199

0210200
535303464785272513779582083

0000000
116789132099910201617271

0000000
116789132099910201617271

116789132099910201617271
0000000

116789132099910201617271

0000000
15225318353213

15225318353213
0000000

0000000
15225318353213

15225318353213

404222151044255408063091799
0000000

404222151044255408063091799
1788334355283363127

01102010375
1188671289114910681698374

706698487517081122233

48197431378350539136

245116931452549257640021207
1340325242235344150

57240761584548982224

703678057307251062194

53271527421476755258

27121286307260378138

25130441265332481243

249733620215324691
0000000

249733620215324691

63339331406368518231
0000000

63339331406368518231
124489777412362

2103612136

2388841189012287

2319314619919125771

3496135

0000000
44913061911206918742835870

44913061911206918742835870
0000000

0000000
44913061911206918742835870

44913061911206918742835870
14222620355040

2608811335143613251923448

175403550613514862382

22562284315801596322014437979
0000000

22562284315801596322014437979
0000000

22562284315801596322014437979
631213221350

11351118245539511903681427
3787316871

1889163396713364156
1077510962363261051217796

117211832332599111

532572021641460

3889364404720840140

3583872538734

36047151454322

11914614211028

34743291252926

35842412311840

3045452387628

26726186261552

10151001346512263

16530862736

0000000
22415727292562

22415727292562

0000000
7977102124196

7977102124196

0000000
1872484278246

1872484278246

493211427668656
0000000

493211427668656

241825052
11087163510911185199810346451

5052569169218498852542
10895152987697518218136202

83381216262082

41111211181291

97213611532

72211016154649

111271420191786

19915726347157

862975151278

1713826284934417

102499374113026232

70092202611615460

1098143337213531233

1532514243319142

4057929497435147

1351126315727443653

12612117271867

126184441353347430134

54415216
868118017714621389

597316416312919855

2241213121318

0000000
82242731268108

82242731268108

61142114302851
0000000

61142114302851
0000000

61142114302851

4725233414185638059350255092611649
18222387416324295155

0000000
4183334414122

0000000
4183334414122

0000000
4183334414122

0000000
4183334414122

4183334414122

0000000
60337542405381573137

0000000
60337542405381573137

60337542405381573137
0000000

60337542405381573137
0000000

60337542405381573137

0000000
5255770864738

0000000
5255770864738

5255770864738
0000000

5255770864738
0000000

5255770864738

0000000
82377566826999

82377566826999
6000100

0000000
521568109

521568109
0000000

521568109

71356060735990
0611303

764471214
0000000

764471214

0000000
341420221728

341420221728

0000000
33281075

33281075

58163927312340
0001011

1613713810

12101113121013

3061566416

3120455936640765160
13357185140581221411637168283180

0000000
83950691025589338435125442028

83950691025589338435125442028
2315233832725640374

634126596586321023219
0000001

56386609609590958171

7265049426547

0871413258
775811208109510311315189

34253677594598630109

4332052448742066072

886371343104011261639262
0020014

82602127998010851562194

6356260417664

119117715213121727
37122284785427938695860798

65356579586428681167

4026855051048768576

143852957310033

1167291653156913972186185

76443912714670968152

23544342485352

26249819611635970106

16143846506850
21710591922153415212304486

3123466341437170971

15535572537273

295511312811617076

7232346943644159690

54380584438490689126

33728312
465191232442915279536331092

0000000
17173423272325

0000000
17173423272325

17173423272325

63383488466459832175
0000000

63383488466459832175
0000604

40348424414402746110

14113629304136

9242823214525

79294558502451712197
0000000

0200013
79294558502451712197

60256486454405641143

19367248467051

30312152157192218502063683
0001010

191534828681713916497
5112436142711

357311710011612484

4214414012111822190

2810720216917518275

178216211910815185

307119281422

12314640456341

22791268710913489

1126811329124011371146186
0000000

1126811329124011371146186

289710613013814593
0000000

0000000
289710613013814593

0000541
289710613013814593

0000000
18494274585849

18494274585849

0000000
10486456758343

10486456758343

231812474208161948617337256315992
0000000

3101523181938
231812474208161948617337256315992

194012190203881896417035252014960
13586245474166

16410562099175216682646355
1044792

603788616807211081148

103678123410689401556205

2623340145738461463
0000000

2623340145738461463

0000000
1711517613812917985

1711517613812917985

110433767723650902306
19248072425553

2856821038310057

3425242338338651689

29101182165139231107

804627537235601000241
04123542

2476931036912479

56382648617486872160

0000000
169214813510716748

169214813510716748

955695411730108979797137792001
1447100907013057

1194938517319

136843933132955841

2617837328230049866

47143295270259312110

9152012182621

10354137316634

22467074719669

8465874629155

6525773916485677635103891174

13818139179

2819133434827744679

43107238253195236136

59288531508490841131

0000000
2412812316611221443

2412812316611221443

15595033293340
0000000

15595033293340

387228635583360308350421454
55391508394385618288

2261861725

4632258958545986283

72231409444446634353

2930550549547280278

69257301279312444286

18324086346830

1

92729117810149301527202

41722453970108

75102204176143191152
2568138

7103123173218

18420137516

4883147132118151110

5320431735531639290
0000000

5320431735531639290

58410116
000000

58410116

0000000
5383946244615

5383946244615
0000000

5383946244615

370236374453260365979
0201003

0000000
946681754472156

946681754472156

545441122524
361188306277216293820

32972104785992724

14343234433635

137811612410214037

0000000
3439931867171817782420742

3439931867171817782420742
2202423221316

92091191719
23655195694910721120492

166283527579
0000000

166283527579

3117537332837049675
0000000

3117537332837049675

0000000
14208348778415

14208348778415

921920242118
0000000

921920242118

0000000
21211931265156

21211931265156

5111887515
136307425476539394300

5643382412618592

1664787710210156

7233416113316

52166257134158170121

0000000
1054228877466841287234

0000000
16102023253624

16102023253624

0000000
2721738232929667393

2721738232929667393

0000000
8152432264210

8152432264210

0000000
23315941584065

9123624331233

14192317252832

0000000
3114940232127949642

3114940232127949642

173321214120
0000000

0000000
173321214120

173321214120
0000000

173321214120
0000000

173321214120

505192738353480317548341139
0000000

741410131712
505192738353480317548341139

9175627394735
0000000

9175627394735
0000000

9175627394735

666191286103910001528140
0000000

666191286103910001528140
0000000

666191286103910001528140

0000000
8370158138115177116

0000000
10255969375560

10255969375560

734599697812256
1000200

53242931262722

19217038509534

0000000
8181940634767

0000000
8181940634767

8181940634767

1005612
1646601303121110441733351

0000000
511617251024

511617251024

4718411109
151643124511599971680293

6113327281415

1254769728617531271228

1614922226720538541

0000000
7164230164232

7164230164232

16853999910159011285418
0000000

16853999910159011285418
001110182116

315216314510013078

2610323018315324467

336081908412265

65308494562527752169

13162025191623

10234828324232
0000000

0000000
10234828324232

0000000
10234828324232

10234828324232
0000000

10234828324232

1084558325367118
1233642369497508392832

0000000
33329877388100107

33329877388100107

58127261226
0000000

58127261226

1034256217390361213581
30569118

1018214176336329137385
0000000

0000000
1018214176336329137385

1018214176336329137385
6592526361164

225552747821170

3327671744839117

396745318916376134

134236482365188
0000000

0000000
134236482365188

134236482365188
0000000

134236482365188

4395113501940220929165432573124248
0000000

461116142522
4395113501940220929165432573124248

0000000
13467994210039411491173

0000000
13467994210039411491173

13467994210039411491173
0000000

13467994210039411491173

0000000
1783146828324893231236242163

1783146828324893231236242163
1272023121014

1261679624283114752
0000000

1261679624283114752

17265750576555
0000000

17265750576555

963877987595751035219
3022267346748075453

3468481353211586

3297771576316680

0000000
49414674612587913150

49414674612587913150

1483467118732079981487973
5371954961625449621416

352494838910221

50284543328318

1711814152156

2058088332121147324

2615787412438339

70131263338610812

154243634258137

28104348113116

25652100346611

34163022345016

67137216159947

0000000
247491971561715017132762059121890

247491971561715017132762059121890
0000000

247491971561715017132762059121890
215447768690615910819

504277346986631010145

25655270695785

3110217422616926697

1297751346122510761838274

68208702513452666202

52384512468442744153

95352644669444689176

14627316952316445116377

75556952546132

784558826566571172259

64288640672614813119

98565111210178941276341

965809229298511297363

261091201047110980

77278335376321517126

89340500446479639152

99661114010239001528203

213211357350288534275

147488633611544849222

6517722325921330579

72206480445354483114

247613914610814565

1194297347856051060222

91324594490456744341

1556411329109013361845277

75281307484437583292

91963751173111915111114479609691359148251538
339753422939794320695412997957226526529375826036407970

8203448067563471827633179127135697
219587102381216457361553928138784819734561012667

0000000
8293633245928

0000000
8293633245928

0000000
8293633245928

8293633245928

86514865780763967255
0000000

0010103
86514865780763967255

3840569159359180199
0000000

3840569159359180199

0000000
14474676498058

14474676498058

17298274625456
0000000

17298274625456

17334537603239
0000000

17334537603239

0000000
15356458559531

15356458559531
0000000

0000000
15356458559531

15356458559531

0000000
1468551465134911811885408

0540215
1468551465134911811885408

754688938097291153168
0000000

754688938097291153168

2526635733127145775
0000000

2526635733127145775

0060350
46116211209179274160

17374156567570

14316268247137

1548102859612353

148913477456814144845568587630540238
0000000

148913477456814144845568587630540238
26083414702831117918831281

22861262222065107776182692895511805
168554789738079613601297

894357603496606948664

289167630914868237840741027

1739742084365317002461582

14880197212548971253623

17112781938205415712598977

1467961342337111671873613

5463263582410476481074862078

109556970181981914411693

1647091112657839081332822

1007681595172713182039711

1838121588189512992090718

376754867155760912000105318426
11225165542335124457299423385423634

292185193232238322393

237364556517652927514

92208265350273430332

5541698094886108

480279408588419772820

6474789910015870

36456984287310951121934

97240330367314580206

100576485150130203

2019131854143013922188440

132114176180151236240

943105734951213514731993

34999620211938165424921508

4014778339027661022796

324632105412038741493471

7859678681129156

1675388258017651292375

119169364362279506259

106130280352242468260

161199463477382583436

115198465395361483147

1064799368517437541865

1824967738137201354452

269330619694501805485

41157202212180388254

1591588679250106391

771065210812184162

1019053105106398161

1551794515283381197341

8363141105122171135

450119118951858172326881301

22610623606335923113762714
143512391699972

742641925181610301802206

856231425125510801654265

285267103809664

2588669452111107

77510113010608621312371
0000000

77510113010608621312371

817319251925362429565392433
236141914252143

2009011543176412822012683

11954610168598341249346

32910891871182215642335710

146595721826573891551

0000000
1579841566157212842172435

0000000
1579841566157212842172435

0000000
1579841566157212842172435

1579841566157212842172435

0000000
325711587708766

325711587708766
0000000

325711587708766
0070200

12364245295339

20216642393427

774272850015059443059462933
245498497815622314783913205319061367682

441100020632044190323562166
177754686885774853857398310420648325

83498704669700908325
0000000

83498704669700908325

19211671663152515122344467
0000000

19211671663152515122344467

967371369116011111476175
0000000

967371369116011111476175

0000000
70208340361318436270

70208340361318436270

68357767750579878121
0000000

68357767750579878121

43165327237270328216
0000000

43165327237270328216

296913113484149143
0000000

296913113484149143

61255374406292617177
0000000

61255374406292617177

17413032348208019672666704
1771043

36186318316263354118

4956612609389881228239

42265438410355574166

46279325415361506178

3596054475448
10166011889009071336244

3924559648246271163

3429041225930344093

25661201059513140

0600211
73199389321295413188

4192199151162204117

3210119017013120870

0000000
3980300238193383135

3980300238193383135

2116381946149812701966694
0021151050

14644810958257241031442

65190830658536930252

1235927837966891078444
0000000

1235927837966891078444

0000000
47280503491402579143

175597838611969

3022540640831646074

0000000
12174938343136

12174938343136

0000000
66207362413330549176

66207362413330549176

0000000
1025179107557511168241

1025179107557511168241

137247136512219441532390
0000000

137247136512219441532390

0000000
6763225727015966112066

6763225727015966112066

0000000
84392748692657909270

84392748692657909270

43295395474392579123
28916893179282426214069849

1086781402121511871679261

613947936156251135240

77322589520417676225

0000000
10635310948118021124364

10635310948118021124364

6183658333640
457253044184255383257091248

31618753275316827604265826

1356371107102910391408382

0000000
1318441484134411721856277

1318441484134411721856277

38198361347318523133
0000000

38198361347318523133

0000000
43203321301301413123

43203321301301413123

0000000
1619701730154616342221462

1619701730154616342221462

167714611510819496
0000000

167714611510819496

0020372
945271411106911421473170

3918546534740564283

5534294472273482485

50755813
551327158445264513574031577

1369001420137513371827369

18712342530213821263010479

22311371887174616672558716

0000000
22106297256209280131

22106297256209280131

71303513476405706121
0000000

71303513476405706121

0000000
58215548445347604119

58215548445347604119

0000000
61304516516442643212

61304516516442643212

1876130114116117121
0000000

1876130114116117121

60401642645586814135
0000000

60401642645586814135

336452609222112898424106207871
12129215383679841704336424477530926

1374838365229112

6623932371536324251921

116453662735665973379

77147288338269480409

995439118498291153344

1005491117100410011435396

26595813411380123020651772

90402663748595877523

61209545613461709213

96239224221236330460

3891511471257398203486

21411092068196318662667969

2286831724149813361925611

107206266315299337468

81241339405318412399

5793171210188181219

378110204706123102210

124335468589444598315

14274799471363475328860

30233163774411017061001

56136290265206337236

6367176191132180190

693946665806151001328

70242696556533913295

109393790605632952453

23210241710162114532132467

64136130162167178471

154308461398358488477

129299482495649603528

458108520491901191126862281

1403129718522005167422003764

1167051410134612321998272

1417161302122411101601683

43411431650168314281924728

2576549158739081201815

4890234178166235167
0000000

4890234178166235167

011121150
24616273519286526524470724

14210582111176616852899351

104568139710979561566373

0000000
564728727837121144138

564728727837121144138

19211642516209718372720441
14194133182944

775338127316901031182

1016121663133311291660215

294131926892448207328811066
6000353826544857395536408046116424

0000000
43231461442412625176

43231461442412625176

0201003
1468431773157014482088332

1014759398077771083218

453668347626711005111

3286861555813
33120353007281025503859938

12910061654158214332073346

101555695638545915304

98446590529517813275

49338513532488805182
0000000

49338513532488805182

0000000
997011195118811291592277

997011195118811291592277

0001000
1347891419122811521747321

9661311369568711393214

38176283271281354107

0000000
15215140385034

15215140385034

101411457496443715239
379225938333504338546621147

1711619817015120480

61407628610599727198

41145222016

1811013313512218534

59278600569536702213

339151222524

6456710929849571301193

2813712013811414189

2316855436138863945

1252224312316

2152531182530
21613492558231921643109520

1318391316125711721793318

83495121710319741291172

27416452931244923873935811
11137348525921

42228419372380588107

27208424299289481121

683956366115381042223

1227861309105710711677305

4157062578834

0000000
88406625567517824214

88406625567517824214

5133165255753883576
0000000

5133165255753883576

74519668573547898219
0000000

74519668573547898219

17214532757239723853681433
0000000

17214532757239723853681433

0000000
3885201168157234129

3885201168157234129

28821894303374834345145923
56293483383340484218

34251

17915022776240222183303514

5339110359568691357189

5522

1099571861153614552348292
0000000

1099571861153614552348292

1247691216100110041609428
8274554841974436884100332254

89516132210139001373215

21031955

61291465451382662164

14174341403433

42466780710693117495

9644962545738

1110424621821726848

69515314

14613467396046

4139861163353869988

681832211119

3122539835232446073

10192963615342

3613729029026945852

744409478207841094134

264510883748777

69380678592489740184

9135661463954

36439552768351

316711112410010780

3617793211

34141163147118179146

83027215112

46317651572575668139

111501926276

0000000
58414783641599930185

58414783641599930185

325311810
30217963868337329864476905

51217382428311540181

1017121758145313652086308

43342789602536792103

1045239348877731040303

0000000
35174324224267313117

35174324224267313117

34468225
16812602181163917872852330

38399682418487777108

8154810397207691252137

4630945649552380180

1237091387126310811683294
714178227916451040138

154670928814763

3724649538034849693

106617119511009491395223
07389126

132811095787241

7394925333621

60391808756627998104

2615222521620227751

28318143451309727934005804
20538459485455

2417536126825443252

17428384679151

533399118487491025204

44269591668546785120

2116917120014123570

6464710818208591179139

9504633316053

19464553467518

12247864526942

3014335028227242959
0000000

3014335028227242959

37114231187184257114
36718093505286528224293787

23415733385833

605037557255831010158

7146111139098691298154

12132421164023

164677132599011321630305

13211512421193017992895339
639394671726232594293121840

1972122121113144127

157170788310035

8849510889129051366276

43132163176141232181

3368133140104172137

4882176178139179140

83758109391710271723158

47218384290305489116

83612107110169081425176

3224635641035750098

7303022343530

9116542275227

246114413611215577
0000000

246114413611215577

0000000
231031218410213290

231031218410213290

21551210519407851135369
6141015

173231506395374456205

36280541544411678159

16687094922989667930114936995
52810221332305274285843290105395652134161

45040184513246132229917240590319403109559
3023112631514714725161571904810574

1428152053175016352522326
0091222

7849810899318721323167

643179558187611197157

0000000
1066288778378531225273

1066288778378531225273

4692216383712233481297324792710399
1339641710944100718865144392575

4224052238038459481

1307011587134612072011504

1054988286736281020214

104284392369329515233

1205238907016961033359

1497631152118410811730307

23913641815163414862405411

116487633638500808214

1834999117717511123204

1575057768076651124291

1937591433117511011685254

2449581821153414852438349

20812452301198618252802426

83274315300233365363

1688371327132412151772306

584356366134239221143

2297941565134811831968344

97289437458401594194

39219063750345528084430814

265572555711027

1898761293116110411885405

1219291722149813682154381

5579408492318721130194622900620103
514364516201652140623251829

95599117111029851284444

307466766802715932564

1194716817597451086814

129326661500441596727

1207171093105010401446380

86347374408356521377

1205452362187317832489875

257170614551347121718651212

21710061494147511972038533

22011521923170917142381567

1135379317368111370230

768711316681456143622411139

1269651383128413522083359

15901567922341925168825912279

103311465388326538886

598464917931735140419626651

9761511139298461258237

0000000
1478911550131313082077385

1478911550131313082077385

03871176
1309191916156215562384421

195581967411572

454618057286831116118

20671198012618075

10497053499357

3628483359862386393

46144269257249359172
0000000

46144269257249359172

24314782413228722143342627
008104116

1108071420127312221909272

13367198510049881422349

28989422992166222329751405
2512268910

40110191235234343149

2714322519117132894

13153826352338

1651571266114712541505972

42464567541521767142

43119164145125190204
0000000

43119164145125190204

297611608159031588113414209007216
20793791961186181132239907915560944836

81236323334283443153

29013392421185120363031526

1217231455116411341588238

1889551585138212602092569

51311052051181715882568649

25912502043188717582681581

248128438253362309560542403

95367532557458755235

44924741969207117182805782

3524258059537621012371

109307465489437606327

99325395435368507301

1386118938427481273401

86107172108123160101

1106091552128612481682279

17611612046182016822951513

1699841339134911941967448

1839421495125712082182545

22214652264212319122656516

1715897648006961243448

97614432349251519093065751

196911131900464414212105737

74289492399313517157

335137823342087187228401504

106370530502437538271

55333881967179417312786900

174730104810629271569424

242819109098410051561373

64148259244197302142

126314668516515786147

82122137151130133135

27917212667263023143900733

1015788217135781004405

41914872420241919583253908

1334799237837621161339

22712991899179516232547565

4438971446160912302006489

29111051880172614682493576

1804846586416621041317

1868671181108610351529500

26210961865152914652151618

2148691478148511891803341

1295361288128512181784250

96663735690628995375

260236325370295441328

28211832663225621823360846

1649701285142012531875462

19512772112178316842693615

167323458455461558308

280170516101432133223971071

67413842370189318432939768

2179041678167813562178452

6311011913917818479

21713392487209320273175544

1287561421120511581728299

66141193204183247145

89210210210171290124

143660115511358881448455

1718401477127213291963394

74239327274291393211

2189741679144314232190500

563447726832183222432242075

161296438477414628302

177682126011958871566325

39178217200197204152

1217031143111910841587336

112483700676554952314

38124209164170205150

1386917418707851246481

1437101069106510361464395

1635889598617551317264

30319804029359233375154981

1429621664156715082109445

13565610088618351232510

45423544597407435305637946

0000000
1429742133171915712543338

1429742133171915712543338

4027256940439867165
663375564405656540482651473

1465318498537471120308

22112082521201319562961432

25617442501238623033513668

796358378548081280263
0000000

796358378548081280263

1527801496121611731893257
0000000

1527801496121611731893257

141220343366300311384
31412712040198717572612985

14754571698574

44255422411340588125

38273485432420675118

77448745707628953284

8337176512659924572543853495016186
26214813634963922133589395656

9223231235511

1186621360100312471494301

266661642617675829544

96410631617554853190

120250375348289429200

64359501449482698200

1155910462368158211

26166253176222198120

127684126110499861483364

214918819712520846

35013032001188916662869403

14987114171657181189

87252318256251407288

64183298410315406189

132238417365425554234

16721374156716671186320963068

1266441244118310391848280

101434793646592997321

9547763547836

78378682535574910266

49175231179195257242

22541211328913355

14553799387010221419285

51209237209177262179

1899991685156115442393558

20746738556867

42138143136132166113

1254588716797201003327

1397209487597791307387

89359982892114371029536

284566121092310111238268

27606747698552

06591495
12061397210378991196332

1045388709027821085252

16699712610310275

0000000
21277445475569

21277445475569
0000000

21277445475569

21111031410
8424460773069196275100312091

47133223215196322127
26418473472283926554201659

9357110999728821360205

12411432150165215772519327

0000000
84296480502408746247

84296480502408746247

492231637673568320950701175
4423292

66223453424328489168

23811561859171016162631644

1849331453143112631941361

441181227552709239232791606
5239252384210939996352635467015447

3781113819
2669381795164414752269654

97540113710289311493265

166391650605531768370

1566209199808461175565
3404877

50323528591491649149

39679311165130106

338411490109162183

31142184184173227120

0000000
26103821409514395

26103821409514395

86645128610529491743233
1196673611831105459593147543660

1058241621142013511970292

1125978558116741068337

3313823526324030799

47201322271260385127

8362011959689231362252

3722948439936258491

45235369355362571200

46151223236182266123

58295400274372533171

90412658647462823228

85423458506427639175

35252433407420589227

1399261546135612851943480

53296725660595880253

247510910165181133

118417912819664910239

46149111221
776240736214112314347291613

55336429382401658145

35257490460431632129

3215020121416628376

11055510088837411136384

1065998418216511091326

8598464728843

59214361324298526144

14355743525650

353196136912320247295

357189126942743243740411146
1951110632

60247327318263385190

1114887216956411155301

35169236314212344168

83428554654556957261

675508517517551194194

2641484
77431748740638940502

26131240171162263229

49294504568472669269

8314511862777417208111032348
76206440364338518212

12584870246551

5343410698329281303126

844901250104510191649219

1148321455124212051835289

1488301194135111071672449

298096989013699

19387182645530

92284401310302634202

867631735155614042106214

44113171216171235146

54292554470457747206

209114310599148105

26774673228859
434236331453395273547591416

81384439440379653264

1056387928286891234337

51180162218154201234

1067061164132710661929248

65378542509425654274

1849601518132611701868420
0201000

108535721576517862237

764237977496531006183

0000000
835228178037161203219

835228178037161203219

412194435573118281544071203
2969825978109131

150684115511458951509385

57401667601559900139

73238403364319477193

5442710948198541244163

49125156130110168192

0000000
1578991751162914332237643

1578991751162914332237643
21301233

2070108127132161112
0000000

2070108127132161112

30140314281260383132
0000000

30140314281260383132

96629122911459661557344
0000000

96629122911459661557344

0000000
95997766313352

95997766313352

0000000
51192345308278382182

51192345308278382182
0000000

51192345308278382182
49189345301273382176

2371

4

6

1295678467557131059573
129526882213273711687810766615941132761

665009888606821080255
0000000

0000000
665009888606821080255

665009888606821080255

7511360937534164650602378720916972
38617624022357632034652776

0000000
10510384019

10510384019

0000000
735361290108710691422171

735361290108710691422171

0000000
18565967638137

18565967638137

38656077069670711041128
1792516876227078637799755918

236679106910119451447422

349131318911535156823062027

2128101510150212211827643

126202267248181271418

199495686569511907405

14134391531

27010961425151412352098844

0000000
16264539457446

16264539457446

133119171621
0000000

133119171621

287390753705737907339
0000000

287390753705737907339

13656910878988041257266
0000000

13656910878988041257266

0000000
26321824752410336365508566

26321824752410336365508566

805329478187441146326
10568610519408491285436

198893888711996

6661134182014

14812222409220318283050321
0000000

14812222409220318283050321

5272540293028
0000000

5272540293028

50199368326316467117
0000000

50199368326316467117

12285532385522
0000000

12285532385522

0000000
35329363407333568147

35329363407333568147

43274522490399624127
6985873143801200211127148541291

2311616622217120162

38432722706603874155

3629830134036740369

13375157587530

54547161261810187952912677848

0000000
1137091526142913131901241

1137091526142913131901241

353208848473966382661361022
0000000

353208848473966382661361022

0000000
19312462320194619172589403

19312462320194619172589403

5103896109238457848111441723
15235315141371091110922145851572

3625938849181315

38890915627564313

1379061982166515482206261

242

60269536483417673116

19289476556755

24384061475736

5123948373515

91112411

1

311288251513

4611471014

0000000
1947471204115710721660377

1947471204115710721660377

8560710799498851275303
0000000

8560710799498851275303

15671281019716433
105385767412728

4745251

17392021324

0000000
4111512151315

4111512151315

410305368575893548079591096
49330711552527848124

6336611848627661112130

29281574516408653125

2311628929321836265

2214519618317928381

12411082562223821122996301

634449668618721209147

37263375388398496123

0000000
571125181818

571125181818

0000000
227658109010408291400523

227658109010408291400523

0000000
33525093800367933615237824

33525093800367933615237824

0000000
7152527233124

7152527233124

346228239883646337653551142
0000000

346228239883646337653551142
124333292559

16710201780154815042271464

17812602165206518433059619

0000000
33404602504496824186

33404602504496824186
0000000

33404602504496824186

320618254307112826326012391498546
31622386509785

203612514198381873617388252695697
617420664206433583788711896

1458531203119210871401373

1208251191108510101584475

68386491568493713177

71284342408308512227

1149301455130412671808340

18111651590163115002390425

43218142216156240127

1179231824141513832108276

52365828624558737151

21115062651245024522928627

156155131122162198285

90427832635560927205

51271738653615852113

746308659865370457677551615
1938211469128711881858419

125677120911148841591322

1456041359117910351770266

91268427444316583207

1927161522134611531953401

0000000
1368501644129612951994310

1368501644129612951994310

0000000
84369571456461695274

84369571456461695274

0000000
17313732649231922423339565

17313732649231922423339565

20510062212202418072746711
0003194

1084268067657031126421
0000000

1084268067657031126421

0000000
975801406125611031611286

975801406125611031611286

4143435464352
0000000

0000000
4143435464352

4143435464352

149651134512049981367536
0000003

176985998414088
0000000

176985998414088

132582126011059141227445
1365848770111109

13287555589026

2415026829825832069

4822456739633541969

823530182526

5405427283138

1465691229714352

7810890508856

611815389
82058291081194738371130702518

64354705570581912117
30921584251347832314929950

9753613109749061422330

846881303110710501545251

645809338276941050252

714829148046921106205
0000000

714829148046921106205

0000000
791618142421

791618142421

62433626661518889270
0000000

62433626661518889270

80517802727658958367
0000000

80517802727658958367

0000000
28522294184377032555156696

14710041869168214742359422

13812252315208817812797274

50316602630498755161
0000000

50316602630498755161
0000000

50316602630498755161

29521074014370334084992788
0000000

29521074014370334084992788
0000000

29521074014370334084992788

0000000
171194351414446

171194351414446
0000000

171194351414446

0000000
121680120010809811718275

0000000
121680120010809811718275

121680120010809811718275

5164297445203546909419716088427787
1040785617779049387741237236841043736697309

24119543286302626434189753
0000000

00401101
24119543286302626434189753

13210061712143013271999435

1099481570159613152180317

24018643360300627384135907
0000000

4324003
24018643360300627384135907

17013452520219420022955598

665168388087361180306

137679121711089561390383
0000000

32114112139100121103
137679121711089561390383

34190356295313445100

71375749674543824180

3190210235157240141
278712342237372343318906279476688

0000000
1307231220118610061758333

1307231220118610061758333

164678838712294
0000000

164678838712294

22239196176591793413822208435164
2056961479127910271482457

213710965628964

51117175235155208146

22111462438248617202419779

54196413405353400149

105278501525393596249

515079757367291183140

1703155431985452678190

11962811739649401465258

1114707998647421014251

135473883768659926280

453988436796601021122

77405457503437764200

55170349447251392190

75511803727586919266

33017883355305428634566605

13147912399728891374268

379413813787110123

7792147196163187112

63281681756552839161

90115159151102211154

377810714914116474
20512322328207919692580540

826441427114310311317177

44263299352398455139

154570976211867

2720242533833752683

17310292198184918212359374
0000000

17310292198184918212359374

0000000
9264467444542

9264467444542

1105678048127291117482
0000000

1105678048127291117482

203107024112214188823781632
1193175288141159112381109569154894177625

78326652597513798322
0000000

78326652597513798322

15910922148192618392772329
0000000

15910922148192618392772329

0000000
898531589150213691966303

898531589150213691966303

010235328
22611261870174915322458785

1559451604153412982133446

71171264212229322311

6192629272318
0000000

6192629272318

0311141
1135107786976741071346

73401669590556852166

40106108106117215179

24612032277199719122504897
13204946434858

63323911716752901218

15105125137114128111

94428740705659925279

2522527525422434583

36102177139120157148

28697276708192
0000000

28697276708192

480302760665182495369701532
25416032933252224123448777

6125730362240

2424146544135948983

41991586012143

83444894816742974221

40276770586623827147

33155236201190320117

36277620528531769104

2110134613287327314262753730317051
9888634501187049201391131127931170295

855171364104910281454494

15811442042195319012756475

147817310714619577

1277211322117110991697469

764981694121711651782291

805511264107210621328316

976044436638

56240481421421598128

166615815813618886

584649649127771051330

42144392276315443182

685069108177911208324

31179390297269385326

2865114110118136109

1128231461134512221876375

42124543390473533301

8058710299849001373367

182240142715

6736011608008511254321

1298334468350

1578671823160715192204473

7774881604636824455699417928

644701416105611251477375

6949810358317601080380

694699027236801020305

595108518498431273234

6753811069239501194384

803761477100110811444957

1206401828134814402131513

63211610815713991

38125261320227340218

11232741404648

12601001099315197

658910454675

49249600442427680332

4837511299039181123637

21715673559267726434018763

1106531726139113951923756

35231779614471665356

4617729731528239393

2254034364935

1329032379178719212693686

1147491167110010091276437

86720213613322391

94015492141169108

15510701811150015682232397

83355733531631893360

2019229526820934188

31518401310773988738103986966

8346711018068261227366

67290594472488687285

65337887753706973376

2860199191333610158855811931110136

5224428325755

2330751541644278775

61180700406510592388

2186940544842

886531378113711301652322

1508441504138912811956460

98284717620576860358

1077462299184419382533566

1136311235111410481494458

1817967046947566161

914101547812239

11193218223519
415254345664399366159421074

1259011421154012641961354

53233404363325490155

9653511089067751436223

704356907756521024183

60420911797623996140

300166433903072267237171047
0390002

0000000
987641349129912161502343

987641349129912161502343

2028972032177314562215702
0000000

2028972032177314562215702

52246479418376501341
252915344267572324322137342159658

0000000
21414372475226521703336553

21414372475226521703336553

20813602168200019073028604
0000000

20813602168200019073028604

0000000
1888561535126111841825589

1888561535126111841825589

9405750968685168284127693328
1217511443110611811576514

1165919589158801260356

1469871530132712371994497

1568751550141314282123519

1457089669308901427614

1369831676146414402445399

1208551563136112281944429

0000000
123539692655549951376

123539692655549951376

37111110744
326188530772647235237741998

14810551709142913042028870

17582313571207103817391084

478327166455481531580311869
775859678497711099394

9147010137438361360257

7851910828338581344351

23216973583305628504228867

41911511712
495355764285558574777901480

23018823791327735514715696
218124847

13010491949174415502545404

988151830152919932166285

0000000
36156215241227338119

36156215241227338119

1248831177111110401338418
0170111

957098798838121137292

29173291228227200125

10161712349249181392235
0000000

10161712349249181392235

1602369758495942492064564732
22011841319

100119102186154191191
45293268636176

21171130284841

15522849373939

19213139264335

1500355857365748476262524522
771152122752419193025432145

2311621610

82324151924140

217166119151117141468

51415747

120109111020

343330515746215

11

2251453

38121781218962296

35

56222

57315443441346400241

2246429

31

15

106248466525398659204

854579008207771033201

111

1121

153

101337946723599731359

413766311

2724729533130847677

131841233

31126746465471

23410161413

1112103117

396104810

66122

333188329562918248136651532
0000000

47164155223132179343
333188329562918248136651532

1608571458137411911898704

1268621343132111581588485

0000000
4175611348113210331519456

4175611348113210331519456
115178353317274420121

29515910

227944

24810423710

11

91646218

3234191

99201110513

715925

252310677

43

411142314

1052817181115

2152743

16142614113120

211136

5921103177

81449833

72215232

639158915

32363119

449542135030343559

10413431211

171813581517

84451241

71082487

2312935829226243551

11614783

9311491218

291437181222

0000000
136312424764270246587100709533

5549153413789480
136312424764270246587100709533

651658117571555143021405960

330262127952699248638771519

327273130372729263439641574

34320323703352932065000936
0000000

44151416650
34320323703352932065000936

21513762514239922353389535

124652117411169551605351

20393118689130382107065101261132420230421
121869821165897079019108678738

1164388162465518510180096696
13659511389168581348944

1664075395134785941216

15252680770764610911104

368106519201626154425591519

88271533423434586329

1596409009267291204876

95377409407412627708

70315225722948201911908125970149601
149219641170893610005680179345203627

387211519691787167424273326

493241539233587329749801277

381971049189134492

256206183171275710214983

153895101389382612101124

424207834511630146920812607

542338934002787295837165427

16610591771144613662046553

28115692252226517943141597

5494669970913868106414388

278160028292559226234081298

24316112349211019792995893

968045715375317071884

1111

994358837533677288837842825

491131517671489130817221281

378198121671804173623222427

2289971466130411141934702

456255154834478430554641583

18811987126055497421738

1537735624334325991259

303170621011829175122151804

792558345003873376756521558

10713635246771859817290194573703
3090114154158530840303403419911360

24311479579769141230524

11483604119638560932099683864

2565517440384419131

603296434712266243231253138

20961141366251731205891198647287606140206
106376061532512317121881673611066

24195405412327560289
166010936196941592415668232959024

189110622171592166725071195

207184336632827281140951358

12987915071114111016191009

111169131190299799753145145173

282207849854005382155501438
8256219525653

20415173734306028734142969

7053611899268961352416

2427753745938563679
508372182876787630795282432

17613782460197119992834951

1209032535203518662963656

18811632755232220573095746

321188332412398257433392670
85861461126689008472119145465

43538909681598840270

95683119111339601490507

3191415213620

5143656

32249441296310324336

12011441831156013322009651

24015833507274025993795985

43811367676820

45758

6361426886940358075551507957747502
16410109331189318155654149945217222110022

182138725632171207230201205

77534826618633885779

6154187943073317336104323492

272250331172304225435243037

14311362076177115492509695

60337258188168278449

244164236052818294838711447

25516433581273328404127966

1034588366426251055507

876238687927201223522

8103113012

8462010698707941185487

16791331722601257932311080

258152236512975291537711335

43415705569549814283

16129192159147214198

46236456347342383485

42130309240214247219

161191069685155140

114757124210369611565756

3617136358

80672111910529451296558

84599642604612815558

2201211151415

866071452113811401522489

281187627302526204335683071

330137726071875190527761261

7958810699949191258718

1589931941144415502118761

959641895140514032156796

25114155120103160102

67570129810059631307917

43219547351364449572

7153430293138

90399688499485735746

200149827622220221534031194

26262486404329621151

4237

23917293230290727244024903

35216275227216309286

33102829497120117

176806120088892313551080

180133820301766154525681231

4211

104785134510629551574391

283155722211751188623831345

1078891655140612801967651

1349571438134912241837848

654138756056421008245

1258731487105510831587838

174112524031792183225551234

205129629272152243631752178

1496338227536491031385

18613382572227121343169817

43292266196204383296

877559749878711407503

1219481464114511551820701

1486115809412987

11323348568381

20912222020185017062426892

46423559444491580383

209412610496125114

906031410101299515131023

52381499495424639225

14120142109104147114

65221359331285353405

1569341912143414131967753

18379312181010102712851119

21415272742235223153217900

392246336022926290843332405

1

43236301297258343333

92910201814

28239347360301501208

12711441726158413692119687

8196353334633

331201240473010283342782128

1317891718143515432062702

30246605512498644108

1349271349113811151542722

977739759567951350320

61416632524497671434

145109821541656175924631063

16512772372221920093019776

24145200138133204163

3785186121150210207

190144225192112202230001273

51360639533544717324

55351465332342564387

3125332343930

0000000
18015482856230422463361759

18015482856230422463361759

391173639463419313145431158
0000000

5285347506862
391173639463419313145431158

356165637713251298143131017

14104432414720

164278895911559

367264648574115371359913932
0000000

367264648574115371359913932

70285441450355499200
0000000

70285441450355499200

145410384186221680715332233977278
5132828424425

9061811339999651373412
9194233374166

49376741629581887189

32223350337347445157

4224039445536246193
28021803539352730554646694

16914292302227319973039389

695118437996961146212

0000000
283171828092502240135083428

283171828092502240135083428

0000000
90263577517380617219

90263577517380617219

68854041024389548220127842426
147014310610715998

24620283883333829604675612

12911032132178616952628725

1308401568150413972146333

16913632517222020613176658

1818829328026942574
0000000

1818829328026942574

1339431517131613421992603
0000000

1339431517131613421992603
0000000

1339431517131613421992603

27421653899335329754665947
13491125

0000000
1249112879912579

1249112879912579

0000000
1129511850157912882399372

1129511850157912882399372

56478557534496661226
0000000

56478557534496661226

936841376114410911468265
0000000

936841376114410911468265

2844143612334722415194062952710625
0000000

2844143612334722415194062952710625
606262941594207337549992176

18212202414211419102954507

319135221072085194329611198

77318446471410497239

238164628502366233737641375

206674100910178401154763

21811781686175913232146698

79408615527602782350

1847821317121210441788783

16210671909170814762277466

122721115310949611314431

19010591424170513202140855

26113072258215018652751784

189110229206581805517520248174369
7132536282436

1298991364135815921705382
0000000

1298991364135815921705382

10924991988386128265117152296
3918825322525129580

108363754564587843276

239397783825826967401

1429061849160714702111386

20412442004193317722771450

1137921614135013581875283

24711012626210820012853420

1217161951161914982104354
0000000

1217161951161914982104354

542361074356430613792691301
20114863074252224653654427

1128581889170315812301291

63326754732696968119

2394102131122191113

34136207221195279123

1097101409112110781876228

124750124711309951536755
0000000

124750124711309951536755

187691117659632
12117499128131211810528162293457

663389065906502554284742027
106694105510198481239338

49289556609499718233

57340647585578886185

886711274130410341645241

994767146766171090223

85462662689548813325

15276371557047

80376659545501712207

8455596010048621301228

24216142605248321803143674
3117036038534135343

102753112910539011464314

109691111610459381326317

0000000
28819193527301627414516724

28819193527301627414516724

250078169713790212606011381817099446427
2742707012280111919985144745289

0000000
39113242282195294119

39113242282195294119

2097699646098204
1932281150594535422664622689

766164832182981266241131483

841519706617649862648

11656810368738551389354

41718291057
6853879836975786877103241507

49231016586597254978244984

96541137810369981577285

93220387541372498231

50387149688145
20336113

55108479

1077101569

91026217233

861217141013

161013912248

39377626680453765116
18410761938209115442288501

55148185233168221190

90551112711789231302195

187613358132101183911039171595678
164637513476418616404

16110061385130012252059547

3039701249107494314861299

814357296645961017179

311249324012028224030141092

92327530405314739238

1257761459141113031849284

639671449444481400063791635

150405745700665880294
0000000

16173432304920

134388711668635831274

0000000
804337637697111020212

804337637697111020212

200512691228042116519233289305519
388250548514627396961061219

998611454133412911964248

101503688661616878314

59257484414400520202

25224104101349232395306554

1369281996169716742387346

93299280350267312280

46266469419407509129

60280492438475649209

3630864161858488982

62320566668508740166

433634264917

46134141161132193101

421256546364233381858221151

94426689702612938253

1086261280131712151668248

2820133719002311174924241593
83868409131671261710609162237352

3652561076011185

17116363253692973721103

10272433182921

2667314652294672405467022865

167334117427

18394838183329

6212524023220733183

2089311673157512651871304

97221727

79187249268239287164

81162

47225487398370496114

77275477408368520122

4912422718621329441

366534764732554871287

98261425461367479338

10549510077848061354167

30232334223641
521118422362043188225581145

38231845701599843110

101246336328258405290

21557555616567

218325504412495725360

113304453513447484277

6211297275616150415460356928915599
18889384180811648215122213545026

13206383518145

1487931547119811892060293

10131152

69296415406393512165

3

1011162299

225431

4337237240638061699

88445761640578929388

15535482577360

129279174140

83184331575240431161

1144829307627161031257

12424326304020

8407662235415

472115343915

16495985868989

7578462617645

1347901284118511041633311

51196405422305444128

26130203151156233116

1811650033738048129

82637125610559771508170

147816825741132160

115162813239

64260547524563792177

3724250944438064266

492111314713

8684641456720

1215469217946291084224

6352613099129011518152

246194139189109136262

986731435134612161886246

32017223888343931974672962

4622537638837354993

796521291111210221456194

5245610157818071303187

10558911729098831371295

1699471694145913232305337

4482152136107183125

57231324372331538170

9273255264516

11407662627526

72176258234223334174

491615192719

685319718657321268242

88318609635520755252

6133825393715

1417345027531343423

3383831384638

798713

682217263118

125517316413120062

9866667555861

994057357376381071309

114198394346269441508

67335356438321525193

24345

21111163

43240418372329455193

8861310029429021312221

16953111419718901450287

1485479147007951165181

1004398788337911208214

613998187356131089167

16313332595237721883499351

1968651615139313091947329

41513161194

874012212321

328987706111176

1

25188267230290407118

191045417

31252514142660

15103219184227275120

51260403372262446110

0000000
61155222194197317281

61155222194197317281

0000000
85348635592552695197

85348635592552695197

1114609339147951059331
9665427990588368366125322932

1297341307120410371657342
9372642184627

219623122424631533

716134526520946249

245675849910461

63380615576461712165

5415134187

20612681885176017682657618
20209312

1449751465132312982021407

60293418437461633199

09601494
34521294380387735855324911

51491017413

55202381383346448141

19515153193268925593869452

197379836481113

71316712712585913188

154764129396210781709661
0000000

154764129396210781709661

453116214014
217210711910312669

10343670465527

7334033363128

0000000
419265459545236458265771525

419265459545236458265771525
0100500

666391479131511601533397
0000000

666391479131511601533397

0000000
11141624142326

11141624142326

342200044593897340350211102
95320413211719942

25177421429366545102

32210489456419556127

735109868968051241172

86431730682604913367

39179291285201343122

3016040338332951270

48280935634562712100

79655910387903769954797
3066740285317

271363102
321288400301345415346

0000000
16287362679438

16287362679438

54913192112
0000000

54913192112

23155021144223
0000000

23155021144223

270234245195231227266
529696808710779

1514835116

32754

741736

313

1125102511

1

561

1032427

2314231141027

291262071019

6211

131177735

525

51421651616

1

26333

81310159616

96121773

1

11321

16

29

14232322392116

5114312

9431071718

112

1684582

510411215
000000

510411215

3791994683543294352355
43572428413

2281648
77218851818397

1231919

512265193

214121216213

3093731395054

72816710

12132222253024
53030010

12552

12841

161316

52201411215

2501342612213111148187
1132311158694179

3131310112

21625

1711

3

2121

1112

3166227

1

275123

1

33681

4

4

1

1

1

8623175

2123

13

251

361

2

2

821

12

311

8

212451

43124

354114

4

47

44

1538731

212

3102

121

1

21

1

1

321

117312247

3

2131136

1

146656

13414314

1137211314

15

131177110

1

11242032936416

3

751235

397615

1

1

3631026

11

11

42

12

141420382

19233533334528
0153314

191041

614125

711812979

455991

110892128

1755316516
0000000

1755316516

0000000
89812027

89812027
0000000

89812027

1012915221512

484586796011760
0041000

0000008
28306051409845

811131711011

20194734398826

1210538911
0000054

593313

7123534

85172412104
0000000

85172412104

82398647574514863212
0176209223

0000000
60240475390363610140

0000000
11152714102221

11152714102221

49225448376353588119
0000000

49225448376353588119

2214116616414223169
0000000

0060012
2214116616414223169

4463123222918

128811212210216932

671719183217

519314681102100124
3000002

20394219232534
0000000

20394219232534
0000000

20394219232534

0000000
8184933274113

8184933274113
0000000

8184933274113

20365529523475
0502300

000000
511210713

511210713

0001465
6517918924

421331012

2345437

7209166726

0000000
2412812

2412812

00000
11671010

11671010

1041000
104541247611090113

0000000
8151510111610

8151510111610

9539105659974103
0000000

531442094
9539105659974103

822611112616

18114101487

7713312823

1371541367

141012127223

139891068

1731212915

19644763605570
0000000

0000000
19644763605570

19644763605570
0000000

0062561
19644763605570

7532539273536

12111622281433

20747816136576370906603817622620687
133058142549611411258238511814078271647828653621

4211125

029197131825
1017031286110410841704243

35226592363430607104

664486756706231015134

0000000
1546558484358534814834380312493

1546558484358534814834380312493
50320813715934776910

361844243327161
0000501

185172418795

181327102065

0000000
191171071396

191171071396

38759512787051515127570
8191453614714517128

3786624461061034

6653978216465036114

72793381813422175

775135257783860

417792943114552

943644477530107

9718349911802368106557242213
6192188419247436299

111252214119847

5315242142951

23

462147839553039179

3544223475525116

74662873741173890365051206

26078981381232465

39310110312327378250

0000000
271171324783

271171324783

4814831831029248566
234412102129

1714431429626946332

821315

3272121171665
0000000

3272121171665

0000000
351943213927120

351943213927120

0000000
2433698163514614

2433698163514614

835781668766376
232716152914108

462029354532166

1923836

1081510101046

32124220

362125371623120
0000000

362125371623120

674158576360157
2537017

431537253833114

22211825252636

54182670458448647157
20146812319659671397561

1691151179514838

147910151559

65148101418

314561019

1133111517

1810211413322

42184310285285466113

18316845516427

10214913452

1923711201139

0000027
1007259498960398

5216138401149

485646414957242

922024302854
0000000

922024302854

2531963462762933395280
3513171420261296

1710188132179

29620132410166

721227261617173

96131013247

911492512052072633519

18093395227325314649
452070497850146

251029152214114

411262507467126

941010162557

1643917273114

10114720123938

9

3432138669688145

599674101058091928708113754937
0000000

599674101058091928708113754937
928214116510711787

1851667529
0000000

1851667529

80509455469771
2320204

2511221552921

53367040396846

0000000
275227202024

275227202024

127116141915173316802014975
4921572672156197604075163683

21232417143029

1

1392514131717

1

1882119163320

1582416151828

14222531153416

32101444211515

4112

45448148367433

3213639728529937030

13184218332417

2310

89121211201626

16131326131815

5191646305018

12221091512

16111812122315

37742253316836

7314

593811162216

2

31274622315020

131073491822

258294330472

21243724274233

413481

101519210

27233931236919

22201916141611

21084885

17393512121116

7184776

203071101839314

25283235334533

53574237391850

15353519162220

29113135345623

38191611146

4918429729722639150

7233313411313516192

1723168211518

22244024431922

201612172214

19142933141918

1112193020493

44153130294324

15234598

243738397768727

85109117132117126101

29354421324332

13251652393317

24255328224728

12264821245321

337522627152519

71319518199

14871516199

2543416171831

62931058

222113

2121253091828

158171193515

8131012104

17122929212820

98377166

43906480447239

5616721316218727698

24338

101097771

17365548416531

331845

734

5231081119

31113817202031

1

345566434410472

95231052316

18321132019

3395476

529812613812913760

28618420212928

239201121314

208141117118127128169

13172518202815

61416351710

6109627710

17234326463827

34133224314134

466511

16413420253125

2413402392623

21

9230924267

1515927426621425932

32611

19201845232121

514883154

20201735373029

32116225

78151825262420

46752414162125

1262121181210

541710171411

152329150102156139102

757311193

66323217181120

317114

121342389

291297988677958

20815318919

12873

261311

41549143

261040821328

2119931122418

241210127817

27585328286738

52681322

4028067249850358268

1481158205

1422114121914

91204732

11

71784779

2223521142011

1

29253126503621

3411620412916222849

19751912101028

1382336243216

4410616919016923052
6789771775149614452011769

44125571

746377879094109

2928566354155673978

126487225505729

26225130203912

14596119

9284752455546

2310417012510126455

3152013134525

1655115848412645

28342413264018

10243636254862

7412011315513014074

1327114116999844

1704887152618110

5123138645234250553
115355670729570806124

5112027326422329264

53821

5135433

38143

933522153424
0000000

933522153424

56207612515458769126
0041007

4019655646942370681

16115245356338

117511472122243424022378630
0000060

46616451355357
2335000

0000000
21221523111624

21221523111624

0000000
23364623243733

23364623243733

112910862058238323672319573
0130005

211399226
109910522005232723342266470

469572911118514281168277

609479109111338971096167

0000000
13182734192339

13182734192339

17152322143059
0000000

17152322143059

2021516291424

9426365666541658649539647751913959
4720044738036944954

39328085493484143535856959
296672515

504431081818773925108
18514393082248023903032479

1309661989165115972101341

5278217521

349319

0000000
47289449352384503141

47289449352384503141

5149111058498331110111
15910711956200315722296324

4212913041411117086

664517217406281016127

21264221222230
0000000

0000000
21264221222230

21264221222230

3629858162858685796
0000000

0000000
3629858162858685796

3629858162858685796

2143460176126230545282261911
1681213171026

0000000
945799138356911268165

945799138356911268165

60793222276183283150
417322225247

12626077497439

228165995113339

226336578585265

1100050
58365849556544822138

2365620222921

3427474952249576176

21544414272741

2511718914011018442
0000000

2511718914011018442

349111816292114106
221115937

8614846263514

1591653423830

9194348153855

9182416205628
0000000

9182416205628

0000000
17666149484564

17666149484564

35587053657476
0000100

9141424161221

13242121293631

1320358192624

11495751493115
0000000

11495751493115

14110181626
26716283631289825853737460

3523063439451055768

11181848201314

946151550138111651730148

704941076789620105887

56230343285262363117

4328249441637062694
0000000

4328249441637062694

221442318191462
1474547972765678976547

5010258151335

57153415142625

85263424272831

1856857119629648

215283827242327

13917348636534354265

38191411151025

69185825282641

59282620173348

168347429146363

82342737253726

106507667756551

725079697977671079207
1712840333431846023

12285739616723
0000000

12285739616723

8375943475350
0000000

8375943475350

0000000
35314450381341499111

35314450381341499111

12406951586240
0000000

12406951586240
0000000

12406951586240

0000000
111248361312323339191

111248361312323339191
6510124711

15313328214222

20343038414140

289518817417716635

13231331201529

15403115392437

14205614214417

1298201398136412761636263
6481274084879444510412275936710196

5415613817314517483
534316757455142476471731003

1659001722161314452166352

20312042301191017982822291

30235482493396582111

8267211029539801429166

0011130
18312915236

1

17311814206

806478748368131048254
1314023419218722951

1015213517614419271

3219432128528942178

2516118418319320654

905211147104110141459126
0000000

905211147104110141459126

0000000
65489101396716251170174

65489101396716251170174

1226321315116110961432207
0000000

1226321315116110961432207

0000000
30185765423631

30185765423631

14065243942985317763111232129
444517150291972675624282355757023

34181295215221322126

71407508566495683152

5617126727124134598

1211391527373

99815697

64356766674617965151

3619218298737542

8415133228525633450

1054427697727141059184

1894187

132181614248

976911192100610371475226

101910592

56312508608501601145

4730973760857483197

20564454527377

561569915797169134

62451431401423621148

102914881715

61352619509457720137

80259347431323512145

64269354415353532132

3270871057912437

4141571076

5513319926618326472

21253275226216258114

2723231436132355870

12656047539348

1209091433139812061842238

722241329399

44362

2512929228326648541

11272330313829

1469401906146113542158212

2392249205819

81245581492513623107

24191819213947

11142213161713

314442027374

966551132107910061562148

2141810163110

38213146243719

294779877210529

43312483421416572124

10301411153123

1619341233333139048

82816871613

5717241

44209258256248367116

79478772773658996151

2102033304014

3621346847036956474

4125461513

20614852596874

36454637374231

3997125757714045

17324550436735258387

19385134353955

4336367575312

51316504456348602110

30334342252823

90565120010629511450183

37202402282309405106

2351212

38167171138151170124

10751221264766108

76927285546507243796810772
44515932894245521713301450

1265301469133511271805138

7212040315116

50176181355318

4518843746437663436

12192421282221

4729657058952982665

215

356471878111328

127181426267
19912332490209719213005337

7958911399608061402167

1196171333112310891577163

0000000
19104952376035

19104952376035
0000000

19104952376035

914209998277701202240
0000000

914209998277701202240
6255268438524

39195499384375576116

202682706910048

2617436630528344152

39308775137331225511168164655221
73878130531186648172011153279216277111633

34547962617931
135218773343293428154143895

946221344122111201738193
28515002582225321363290552

93349541412436672154

7532164

221021241159911140

63367545449446705129

6552554354832

194446280525856
1005304578518536631263

697205445274872

11254

113240461391452525131

28191041018214349
0000000

28191041018214349

757315111996146100
3020007

34242931286139
2100503

1182017143318

211591492818

0000000
20155636373525

20155636373525

18346452315029
0000000

18346452315029

35183390290324202672297914788
1127137273929586

156745184708181
0000000

156745184708181

193194379352335479230
168615514513021943

36335741627030

35143126322338

29142835242025

20212215231127

2152530145218

642130114812

159132126217

158279131530

608280483345386453881
105165515721103125014311354

211

1101659167

192915727

786156

2211

4718758641340152546

3121

12

9129986232

2213473

161

3

38113928553554

126183281324

30176442549562

1486831020224224570

25

2

20120910755

23614

2215618311827

15378446710260541
1510124132725446527711965

60575562103901431

53935112

14614217

64145919722

44111519341845

4254106

217

5731112

2651146918

1133108613381176799864

17183332353623
978312793111120130

26202413312517

7153363

15211817151623

716810768

1372412141236

1215661920

717411212092147127
7217761611

11232747221919

20193418292641

17151512181928

16151936176728

328998298387310415315
0000000

328998298387310415315

5377180473353430481279133913613417
70128114140139175123

1042995321542426685195
0000000

1042995321542426685195

12675302964288198503113543284
272910330178241637915806215606477

21

21340580473459603102

1282

161062

8820955744445044599

179331416386404750152

2407611312216048

126414914514416323

20150281273967110591117

10049311128488121226223

47162512227

451411449110413458

7317111

94271311

4151923234127

1153841829

5352068

355151365

10532

11385550284028

7133941366526

105765914912811

156372521301

2494995

103852532

1722444638840450551

775899808018461249158

25412

21105576

2712619024714618559

2

1813124

19184237194832

36412691783185118792382571

3623836835035956544

24

25412775315934

151212206217198248215

274135

14512883368264323403466314
0000000

14512883368264323403466314

0000000
611912161311

611912161311

3101249
2088501985140914241900300

1023351161700751915129

103514824708671981162

7856611909619261394144
0000000

7856611909619261394144

1228233348004473381055253341
143390906736687854324

1896291610134311751639307

154415815708615939151

9555610399717831416144

465176166371304361426

136721441591251611907

469512018512115582

296193899711340
0000000

296193899711340

92528105290710041222157
0000000

92528105290710041222157

1918064764063204191201
688165325671926192530832315

21322326253595

103172313117

254471584445156

3348122

411444123

587740267016

123178543126412884338

12496365414610

203851127768358

58326

15154025384239

1015016113210213373

752617223335

22406949747146

165013716111222030

5101338173327

238418719413520530

1381718292528

4321754138039265478

105815172616

14102215161827

1812321224618026866
16247188137071242211421167472988

251071291319413496
0101294158

7367860556434

18613962355554

1007411418132013411839194
23213402660245523823288443

7971018364

3715730636426435557

72385868707717995144

16486154426344

2114324220620330046
0000000

2114324220620330046

292782115212539581529471
709182532753106269340751103

8727144342339260298

78265581433410685119

4237

35114637145315

5117543

48254565530499649110

7262127284116

491424838

55310111012

16163827122916

24223937456928

94272430363

51198121514

182215810611015922

6163638374223

6256

16301121162531

10315033324430

32521613

14223638563544

23242423173414

609363871746257584986631221
23718893779329630394684560

2213138432230848031

7171731142113

11311710

10247611659189581229144

3510814420014518932

1

12486139292540

32

2419829832029144736

74393725568562887113

1

1

22881091069012041

532168218

8771231069111550

57207335333306435153

0000000
10121521201913

10121521201913

102651088911103143619111118987316
46368757049302585320736659979959410

2122

290292520465489671244
0000000

290292520465489671244

55320732605629741110
0000000

55320732605629741110

86328515647485045407013820
22012062629212421243366257

3917328527623241956

66187256385623

5836875166658494077

1536559313014

55623326194129

1496311164109410781390193

66293855316627

18293226194224

95527135294021

33234650194628

4925746138333657771

9248511498158701263134
0000000

9248511498158701263134

000000
910111912

910111912

0000000
3177118117

3177118117

214

5784
0000

5784

8342141

4124205939423438311944682129
3287590121295188112501444

3112

1469791720152114652192227

34

184398870764625860215

3302138473844140

2429126

1696710014510911991

324881143519097110
2338248437205089322242531213

1310235145

2322

77375829801661976124

1

186319702645384023823059905

44288051556247

29204019274322

0000000
1157161325137410951910143

1157161325137410951910143

245424

2333222

22181

5806731097128395315061206
11716228827925740970

2983936158195488841088

43163326112117

657513614313017910

5727251671321

121151

241214

4977304642985039512446334416
909580942088513529139281567110669

1617

476851

4

1657158413

3127855838342663759

131376561420478524158

7452303120163976420355374304

174411101952179118782519356

15212526103823

25636464737756

31428251

124

23125

125

991322519913877662320

1

143206343348305445557

282131

16113315241518

122

623331242828122

244

122222391431398450155

1111

2

67885166656683

644312

46425714715426439235803864
19412972408217519282945383

52220266355323405120

23473459514141

1513424120320433825

5201110375

1245949928968961307193

4024662550745365258

162812958587839

2288441764150614522173310
0000000

444217667545941053138

1844239987528581120172

2534841

41136

0000000
298812910912316657

298812910912316657

70298577568475783101
37913762293210318552639579

3531351844138764278

272724119410879771209391

11

9312

5

23144939

2111214

31532910
28576299463731

624273530166

611111

10242748131113

382121

32511

1343532
124814101220

101142711

137327

0000000
5786328364722

5786328364722

41624410

744699
000000

744699

0000000
25372912311841

71543915

618175141219

124848517

31218006565452224675659611327
43811138510319929285641195320136

11

25

22186

5126364258660779688

111

13

8011964908180111

11

75368808684641931122

1

118113424287

12112210151424

112

82

221231

23561394

17134258111

11

2671231057365667552

1111

111

12252855313018

31115

31431

1633213

1

63596858979653

6311

73

4201174835745669893408

1

1931253

3

3

22

33

25476761547645

511168112013

1111

1

14693

1

47423

2318140033835752148

121

66407688672542893103

4111

31

2625571213

53

12343917382626

13

433

12

5942069456754477693

26671

69376914

20365320343428

4

772328
000000

772328

1114300107
632155681886147913192241753

964799307686851108173

525149469567116331133573

122135

16288158150173235189
66235353508772

953715202

11876233

50326434454773

85161638329

18231433192630

332

5341225

1311

22611081851162215622533464
6191315132830

4135668757256591872

22876839359046

15764610839969491497316

1551160127052272214426662898
76969315031157108313181615

862322202410

591395131

11151

2156267713590134

7712299811337679

1798620116

1114231920133

6266824

134

43768096090082011221031

432

35717

0000000
91104449

91104449

4619581540236852687
0000000

4619581540236852687

0000000
9559912659838791446125

9559912659838791446125

0000000
3819648644939550287

3819648644939550287

623212

184163

0000000
4922276

4922276

13121

153136151819
1135762408510091

7161

21321

73312105

764677

84233

816156149

7146108919

7692658

14221934

112

71081342

712862

14

1819351111

5521229127025638265
676570832773675967515

72186388366272415110

46836560555776

575956

821111026248

4956370625784250

7428771

0100060
123722713

952219

3224

1132

47310658
0000000

47310658

32310141

949162

41231

0000000
30366449656348

30366449656348

89439666121411269810933145787576
5865548956405928509566004839

204236218232193266131

139157155167280213199

12422926212736

247110111212014049

1552

412114152

3324229631225841065

15363229281830

9215623235023132358

6133

21

2324311272817

123614430109

422172

16381838322532

2111

267545100910068811357216

389324266230198238628

1999137356620

4613168407

305564136644996

2312

18423037223727

37445742556044

2

3118252843942758349

6225293

2173211

11813

156915419920230322

104213235346

10122532

12687047723642

311

469116711

6671116108

1

34343743243830

152

3

1234

5420735433731345298

2281

155165226198177276248

54299310396307495109

6111013143214

1

423

27142929152612

22113

11

3227511616166613031740296

621715861475510

9917737

3515530625324440934

525121385

1

564127

131894121331

18224041494923

0000000
35617810610412465

35617810610412465

133164258241252359117
6810819118719627543

41455032454962

623122

169411491

82794149

0000000
954727836935931100163

954727836935931100163

321

0000000
44946311

44946311

3381411

11483716
000000

11483716

3586161476119912221673401
11370138952328323267208223318114329

0000000
437237416338369442333

437237416338369442333

0000000
641374106

641374106

8327983878258682494
0000000

8327983878258682494

673133926492266230233013612
30143176184117202131

8748210427969291220171

2412355654234876061189

756297985813472

24041776976571111392049

3001013
151961103877884485

87372331263141

6159873555252441

440292322359369699256
0000000

440292322359369699256

1914204231633641300441791415
18920911539427725199

756186332348253361356

32384953547366

233348587011214

13781681585828087

4122435137432351155

83849521212613068

946684881109190

26224454404430

33434750467256

14511314499118146113

2637551493142513001693251

399518018614325371

327595877113743

21181855152516

1431112315781661160532971116
73088990142961428812888219697727

171491071567711256

85356977589496

801332104125104508628

8112825820822727173

45664

21172012272115

87302683640632771177

512543124158959

8516312710612358348

12172979722

204621111110869241364719

7921033621528438095

13022755541541364895

12714851842646159374

334378701586481724332

25102353224214

111258357401320615270

190121104116121369145

16220539430729352486

2296232037124313379057112908

1286129113

27151811126510

7223139631728544090

47617272507255221653496426

911262295

427471010

63418365388335

13824539538933245094

24274352015622

26415822969279327073681485
0000000

26415822969279327073681485
53410767672662993102

1012494

2610412916014018460

23416568517333

4016125635430644055

1128651752153715441982231

74296526196657637107637817980519278101382
0000000

30211609366219594995847
74296526196657637107637817980519278101382

7124512535114638710631781715181477661119
4565301548142325537021087999692828248

35219991134

94361692272323

1107949239462530

1705123859180632

998281861703511731044

80468142131427

133731722911032537

107728146835561312364243220

1009717391981287331159328

12766479441515483102

1349421239591545105679

29210241150539114

4596692332279083125

164105359016145

15134663237784772334598

201197335

78512240137512354

37613726182725073

15210544303922853

260137701436137134

15814933319522433

2553254471960585557137

5521099874442237114

257256821281057179

3324777368

1574641249412946

133371915111419

6051247158945009242

672820146345

154149404714841543

6100114325542161039567150167315541

315744118662945

954838391114

116163492637

16686383806437112

105801038221120

61

23811034274723758

12133204712

83410331493011472493

7133101201319

1111

2034179124059342779

1533330722697600515297

1007734189301785

25360418465330354010925342011

3954298568918996234471515

93437098128530683197

892118112511326

303417601168582327

2

422265142163978174

2257660110833032

53406168372462

128908825

2518179437913

895434256193031

146723026743765

5871415692112738234

134937915313204175029290

45320837394451695

884426145413068

6526164586177151106543773

1807437260363145

64113215226412915060

5721612540785329108190

228642987321130

20089328030268

36485173781516

411422258707738168

287813863624

680272538236060139

9942112738134454260148

1404166957382225

2592151213411896078

2981681245495564

11

132611483322581394198473

324258683001331159

12563647332510034125

9543216336113

2174752

113364018346912740143

1948245344411349

754211176939

146634596925989021224

12672537261102759

171616220234432726

1935714291366

2795116947102854

7614446643842644293

148221020035117

474264163041053674

9462729165136674091

3559537138494054

1621

212969547610917424728082
130734140266357913772197734381

2462112224269

282030241918235

261914121121251

211323161718274

11455524

126821818305627493

3323111295675340

178113141372

161055264271233

1436443717145315

835679985780599

287549355112315

86872826166841832517104021455

39133236201994

2611156912072571330

42184246535347
0000000

42184246535347

38787131891137010228811623
14378268881795168351323774988

758121817230

2153672311919149

1434741352531108

12218194272109

12848126907891109

4085827121013

14881606011386238388

1119199432997667112329

105194130251644

54916204625

50851204188695182

629387917658619798225

243328054511578

15312542326454

2815273373010145

271357226491093506213506

3

1742640483621120

656212614

893414546432667

125242419221480

13116674327747

15691384931376313392

199555644333695

12918471624756

0000000
1399991853153015371996321

0000000
1399991853153015371996321

0000000
1399991853153015371996321

1399991853153015371996321

60308447482375518176
0000000

60308447482375518176
0000000

0000000
60308447482375518176

60308447482375518176

103657716688616869344
0000000

103657716688616869344
0000000

103657716688616869344
2821412111489161116

59402547513462649169

10261222131431

6153639524528

929219737833433328042421798
1443313

1724558740443758762
41710592069170215821910944

15162126222817
0000000

15162126222817

13216141464239
11214734353224

2337

4167

2141711

22355934446149
0000000

14152617122621

71028719922

11051013266

55671288074107171
0233062

11103310211639

17132816104345

321221511912

1372023191731

11142213131642

0000000
17213556422763

17213556422763

21822232395
161493858803758813309

50847249786252

3621144442938745182

962829191915

1192217101219

16422619312634

17527999846451

207116513812617051

0000000
20154629272619

20154629272619

97146274229132219215
8233347163540

15296016172843

18357

69107656

27362729294743

12119473384826

28384257235150

20263753385213
0000000

20263753385213
0000000

20263753385213

1919531351147714042012517
0000000

0000000
168984877410142

168984877410142

30291413478441592133
1758641267139013301911475

10533078414030

63261470440482720112

13203632274053

3515625731523844287

16553631845444

8282516172316

300155322198253267321
2043208

258138294184230248263
51324436406457

197241151030

321312

553611911

4261

212713141322

721385

71824158

14826818315

192118

3983924271917

620201316129

16520811914

1883724254710

411345121022

522812132312

816161618621

40172411211950
613103620

131051046

33102129

181347715

0000000
36155528333344

24114320242519

1241289825

16528921152261512512664355474638
1253510

0000000
543239906886871116216

543239906886871116216
13312933223733

2422988059761399792

17638158528291

4523136837934853092
15978596142311443411972344304422

4192529424347133385221911263
55296307333281363182

5142318232627

8794541415027

6431769055962387284

3416932835027337192

4336271257255074293

19736253625031

18665557558955

33132273123815117651263

5457610007617621039155

1477791035910178

1816229629720340633

186885606210678

3613828827124032565

0000000
52243300352274437156

52243300352274437156

51267397438376457125
418283239824298377054191537

11259099811309801369721

43491579642529820100

39179148141154215120

947349829308591316244

7957187810178721242227

27207318283248309100
0000000

27207318283248309100

5272039323912
1285801037839692952299

80384660536456638153

43169357264204275134

0005000
3990213131162186100

202312460928932

19678966709768

44391633666596872139
1311625025023134248

6404234413325

2523534138232449766

0000000
2929892272191317742533458

2929892272191317742533458

0000000
6112326931922429767

6112326931922429767

72381596541499704211
0000000

72381596541499704211

0000000
22103321201411

22103321201411

168129998110039291326227
3399172505110552941552612519954653520193955

0000000
981432678097051634089591841

981432678097051634089591841
2238771504146613241794446

3315725431024645784

2717927528825135681

13121442445335651797

5647610527777301023101

103113014259

30455055486437

1542103866012135

23725842706158

18354031395024

1319152292530

8171114322315

73395575563518739138

26957867577373

2616856149047861755

4432177360353173095

2112335141712

624193382032

21353424185034

4221132131626430987

4717322323216531171

191035265678339

2720336328928347632

24816296667958

3737092866469293998

1810722517115522674
0000000

1810722517115522674
0000000

1810722517115522674

0000000
131179610410313559

0000000
131179610410313559

131179610410313559

3387372499261543830544283512427642874191754
503657748157101553414159190396052

876078626487111
3115661410

47192920263140

20191816172020

17112620152241

5975216946581027569115081338
7460711939827991136197

70569760776643972178

25021594820385338095474506

20318812692249123183926457

3248662385423491539494231468267579866175748
180741127655821573823087620067625651185865

61614678193140

3187821468130512451778194

13516191690143314351873267

26332227243918

55606272605176

1659352646847

9927154

1647891244632010111474191

2057611713148213711850233

2112675445144964248

9035010794829294

125312229174

1997201529122012811655199

53912978469948021131513

1736211090112010141537175

813114181419

3937979453458785763

2531458652247074543

15310641872149515151959233

24768599810508671634192

11121212410510113590

10877101845810482

1574338528395414564118

359363486473469574142

18311131968188415342505238

69296292266242331103

11094871075770804943284

5113734112627

342

8632444

1658231517122

8181646414

14701323124410

16551768645

100364529449421667134

1368371414131312021676216

24471120142741

91814264153432

97371612859439451466186

6740321516816618379

901127085848684

1213131316255

1240172318246

14233352

4429147545133351776

6310361718

10734450453952766698

138271602534476684117

494392155279187233215

478717318317427132

42296989411112498

27019612318614918595

421127141383

395350366473441458297

4625627726624729867

23717815721

121387499372375568123

624271279871977114297

5426748140039854684

2423335941627957247

2585049407067261039191

206881318108

155132511

74438677518560666168

39725923787309730264685559

27542817232926

16423311400116112111796322

1424759277907341045189

55614372359227420732934527

136265491503566516192

6510111470666636

1725297636525821014252

155454697699678836128

3143785451226534787322

26674430315319

281692036372936

12128327133421631085

130322431309390490123

10266234738231448757

431108160598253

3526526429627742559

74461261296239365146

154173142149144177114

534737

1939509908938331226233

659956561768558

17269587585724

4314120424516130956

3458511321128111881831278

4667641643122912911777300

16711721394121210381614242

42410121869141015592249390

19513242342417

1320251629

7101717128

23672161249

19534798142135172165

109249156184186203113

22312112384194819292698288

18551218162049

8608201190102299912921936

73152152169107168137

23711472139165316462403314

46386013706924379951714246

100390800646613797103

9728855049538069073

45191113131911

89554966766054

16717434073300329144003372

91451315133

27318253635293527984185457

184625951768736946212

5217119712

1001386762856780

134242400314293467100

239283106528624

1747961310105710291589201

10676881125981207

241296

139367269256241387163

3644159111414

45209135233137189114

121404749482484789100

88491546645496649156

4916832426321333933

363775695691720971491

256491577670604757221

4445051042866784992264

26373271426423

714293018271

90201014201211

41917262561233021463251548

34474937112224

90607568594501587179

146335630581513876106

674374682479563561164

652151010147

72994977876194

825181851917

2112361112

8726949236329645180

82645187528463

61314161

105782598496529818159

11251623291713

1766459998589681188176

217160244237207257120

18112076815410442

2942412191113

88424658563544756135

31142

18766510689497921316165

3212721316815227331

7121078611

4926054140933658281

6835443944239952566

1711258107911159191524732

10670112689829061745165

280566109010048161235229

65418286598265

104648321322333487177

712565158

56791239

522192501025819242

56822926313152811

135297636546537637160

3916629130728042257

5828270147747773648

3617120018913522479

17214203933284527893814169

206336265

3530324820139

3621713091115142108

22111723182

651987562394462

9417766636716

12785410639718321247213

514351738789656943303

12212588217106128111

101343828669622711112

15912841793154915512481265

14185111999058751116241

1481629084709275

27120333919323730204555373

471111

158493743596600873159

141308271296272404157

4911710216

886365603433013163671095

8642610267927681100109

825934228826047452

27411331800154313991929353

11517313618512923154

291391171299115842

109116977321199275

123174102102607992

75782115

5311222321517717424

16710851540124211121732220

591451311288913738

2425849537440069875

15171175937482006189219043142

1049191774144114351904159

13355

7094273411533567

13673113

11591019923752104190924902216

122121177

255867637673498991176

557896793657653846352

1725536424465388499511

171620109810378151401180

129718315914020216

331015054493746

62713236

45224

3123912610

147280275294279342131

1988991707153813942059240

5318829326527231778

15425315819614519097

17392774821

8617318220717623158

33571091488011130

41353933372823

231148242

62628811768717912153

20117152039

15413619818515321363

15923001794156113731991306

6029261344052875656

109627335306276311123

23815252536222520953152373

18712824172539

211431579526409713152

284531181716

8128743440032637396

1777941215118210151513256

35315382007181716622447372

66524426122035

7237213196

133242348407367547140

145596699768610863239

4326143246135650877

1315151217167

121440488493408608114

1523333763345169

1375917967096581198176

24616510593788460

812118987729686

47606058486446

661091354233531

123132176196195261194

8863902792573499

645525645597553786126

105489893780686987108

134751120310919821658164

32383

12250610108407771126145

42423703090273424394000697

6116660627411275

1878793269352258143968

1248151744124313111887143

1688371208120111191586262

186406496530561670100

1

681151214942

36461225752121533684

6030828334529242768

94655210202202245156

1746249669628291281151

71095639

8625038331626335172

1201257386659763

12425617421715825763

290160610668699781234290

75281161386

65328391397341571103

120404653599236

2515734132224830885

9736838

1606049866704671

1371297135

2

31520360137632381

9122516521414924572

861915142015

46511951464140212141535366

10403118393211

302315410

1001382475395369512165

91701789691639996254

218135131615

301759101910098741053283

69820720418223526397

634

11910311813616813064

76121816204

404135701094932154

84793675310283727

1240333836302

27373339363829

22

9735618519466351037327

6631328192633

918135938

115257613812

13569118141679380

34674857706346

731898471798851

16115771734168514672236386

88387587620534832102

19068011358989631278287

13422197817106901036226

15011401903162515522291224

1589783805411988

7325638140033561778

26716232961245322533188314

1454411009813768956104

9304126114

48352289517262627

7032939642134143376

2159011707146714142228203

6418022825626438474

1457011531115810761598179

158104556161683028

160877122510879721362212

110353549542401667163

69957974467665

14161632283223

7328759752247051695

1086061617123113691747150

194409244135151202110763155698630

11322091461281101021297

214231

1824843222

9999545156996675747906428

79991451178411362

177302355459405468136

101233

148756116611359061422187

1054858368727321121104

57544359408345

76113971039513577

2158641799154814862124189

10651831489430042450

304653032524024

281241353435249460306

259207250379660330315

541492266415207276287

754019996221954

22583

3310617919615422538

8233328305113

168504594548445777154

44963757255130

1697477421596987910191

32743653495614

121

91320349428344594141

948511813916113828

1312113

13107110848014140

25158351015

6930952745643054285

6119943336839552570

796029174183582

3911042634351826

1134599168488031035102

81336951508985123

198136310449108431344294

572815882384

761604546305364

97206263239190245112

2145907628026611061251

4324251348448767068

499522210

33571662014

31339247286194304110

90211118155131147115

74218195239173272113

564339617567861210101

70566264321291429149

2217671212117911511443254

1677111755125812311647270

6829158738045853768

18932552843049259278

1164858198097301099140

2877131693

4823230328631642677

2424278316794106183

348226678310008598731271201

775121425

13458773119108981194

19445593580510161116582

351534596122131143510935148185240

8818440529031641238

1632131

11416438125024841135

63838076453943

8721122020311917

1264438577966821203163

23233029344527

31745843505585

524897618

905185822592161410930401362

1508381603132612891910211

277424533538464762239

1

21841220

3441977102612561651

38233627222812

2448135420

71941342

212653

47616904103310499211470486

1557194125772480277436371543

100409869750726104486

86232813712062110498

29923556426327

84509643572493714103

97106113777415634

151018111716

15144164

87279572446404509100

1271640731656627926169

273677108110719391320288

26263527534320

481274856727682

2217151827179189

7141428528

164431762693641873114

1659477513557205305445

21611372299193019482731335

1755245426375592302142

124359161216156164115

810791610

26075265165468338

40157498313977561065514

12435211510

6462392616

63622313252274527374123503

65239352295275396155

21375161329117

8111885218617170

8117819920520025548

367870122410349481249274

4416543

166432517475388547105

5261

26916237345920

37411

2826198797937281109195

10060610248638471338120

74527793542332830264275867

3912111452

406023537041433361146

1544621101718

29159813512

20718212590245822853696435

25190638511313218

3622545535130652547

22112966

4291796129

2198121634140312921761190

1082414

1516561499125911181725171

3002471472996660605087672004

28447364526319

111114106181

17418310710677136132

261372218292330

34125173713974

920262127265

2384506766116126711155

308247357650453341344

211311202215285

89309362375294541119

29353344342829

27816241544224114451919388

78683451363641

0000000
66577581768831

66577581768831

63358630543502711127
9045048875075467072100451839

20211041592126212041881391

15311232767230721572994258

34767673698970

11255910198959071150175

2511568766210471

38357454430391620112

63178241278182242166

15751522152018

606111415910087103

3425039633331250682

625649787998141187184

4322840036935745482

0000000
351481088712411092

351481088712411092

710045466179871855615063220566492
28662519053265229430662551777

51380791049011576

19912337619737241223343

312910433855491337231106663616

26342196567367795

966718959488411099137

13611342435222235

12601527222520

1871351761722631889194

301197397549025

1316761434122111061595174

0000000
469511884337551

469511884337551

15182433284023
0000000

15182433284023

5222917121821
0000000

0000000
5222917121821

5222917121821

924181510128

156611914514417141
572419890319322970327710407778033

25157149135137201129
0010000

10595152578042
0000000

10595152578042

0000000
159897838012187

159897838012187

101041271339816248
1026692711141103039382142282800

2198681177211079
42127203821362032964895962

35173326239241296127

755618807556961020150

117832108410879751427266

56130147109125158106

1179261316131311871884234

0000000
44281300339262433114

44281300339262433114

0000000
56301334393325515144

56301334393325515144

624658876906941027175
1131000

7405548525248

54424829641642975127

755751342117111291732264
4331526201339

34193380328319463104

373499478177901256121

11323710253
18614302401223119593140512

4117527427122641760

1414521821719029252

725298268527131091268

5856810608848201315129

7497335445282
0000000

7497335445282

16510021856169115752272499
31171315

55338620639522710160

8255510438908971354229

25108176161153207105

0000000
1207561154115011601485360

1207561154115011601485360
06352117284

67406680639722826178

53344439490421631178

41068961138911307312293179393780
2779124857710176

2827197
24243634284962

143191241325

8131515232730

19010801921168116482229466
1162719352433

76344619491530679163

74598112910389341343153

39122146133149183117

49316415398392595123
0000000

49316415398392595123

1017341191109910701625243
0000000

1017341191109910701625243

41291441517417617123
0000000

41291441517417617123

0000000
16813522144202618872862380

16813522144202618872862380

6162717171311
42118126140114142122

4143625243525

7201926142531

12191638122526

13492834474429

14910951922186516802741369
0000000

14910951922186516802741369

106311711113114724
20914632363219420383022402

1018521300122111071771228

985489468628001104150

0000000
56455563513451772166

56455563513451772166

302857257845695
289810461126107111411145850

269109294174230254111

107463418414036

1265360204249207190304

42246044446215

913422462529535543289

4101530173520
1358911508142413122031368

34178236290217329124

977031257110410781667224

1717112638830
0000000

1717112638830

9058110219439371261217
0000000

9058110219439371261217
00100522

4020832634026438977

50373685603668870138

0020000
109523132110299471250216

0000000
8474067526253

8474067526253

0000000
833961200871821108699

833961200871821108699

0000000
188079917410264

188079917410264

0000000
23319193136292527104242490

0000000
23319193136292527104242490

23319193136292527104242490

16794322526241422706619880269668585
0000000

26382817482649
0000000

0000000
26382817482649

26382817482649

202371835381639
16768322488241142704919832269408536

8211744353823
0000000

8211744353823

099961520
936158677227751196255

25897168918582

685177876456781096153

16345320825221512512517923243167822
11280215837790412008584875574026

23119155491865423

1139774111

95396

867151282113411241607196

1951011114910829671507239

41714104

334122271

79920112792276724743366714

581122213172

3246470606610038

124713336302182

11753429101312008201252468

7805357808515

121683121

1256782541336111210251525334

54119437431430539547

23101061219

174551691428

5438320216715720274

11703181214215

9743059346120

424261

15932417132220

57337489390370529109

243433926374113

163113

82051618101012

143225113131

331517855568428

6444146

104921805619652102592

446615121314

3441041320

2731597518565498560353

544226

1171015461

29238781228108710871369388

15387126117

181103631093

64741599450405585118

3796152

637157102411

610062625

92803048463720

21354101772162314992156262

785807458087781067162
0000000

785807458087781067162

101181841510
62699776498556

17221721202313

1612432583013

19242912171720

8112503
162141219239234222179

58195066636846

96121168171166154130

0010230
148181510640

371011317

111745323

0000000
131578117

131578117

37432576089544850327155966
0000000

37432576089544850327155966
0106752

0000000
1059191655148114021860287

1059191655148114021860287

0000000
8160511109918451321281

8160511109918451321281

18817323324297027783969396
2622431938530244638

5550310428918591155130

10710051963169416172368228

49117198172142223128
0000000

0000000
49117198172142223128

33134810
49117198172142223128

15101212168

1717151416922

13511381079613935

6171216102532

924222042621

40281267336273362145

70410280894340824317049308985423490145556
906605410976101309393132933714

5101389037959969215622369668530301
183155535413089270543942360

9977491155231330811897193025272
17214302591233721703354945

172150032192795241839801170

997871793157514002305517

19514223777294725614025782

162109221151848156527561048

19712602028180617832882810

20300825553550688143
476399976956609615194321858

22318073600304828064414738

1118471336133012261957392

12210451934167815692373585

12998967176941605314679219233656
10351010577997611307284

1076651327121111601605362

16812132304202217472753578

1369551868168315252186451

28418853712345331454497534

1096281647159413561941359

23818883988356234314978612

15412231791172915542656476

1637129742885924293219263436614173
12579718871508128421681212

11810991971161814602016889

155122844213644329850571659

156151045863503303155955305

14111252462211119472883892

236195641223547340948831096

387290151974653417564321392

13110201620149613262167934

18813382593221319963165794

12411251662151413921957465
0000000

12411251662151413921957465

0000000
19713892172216218412749783

19713892172216218412749783

0000000
987781491121890715091209

987781491121890715091209

0000000
906259629697381053525

906259629697381053525

3828157781782917899191002135912060
64403235937250249237704237356313512111541

15599433157971371713080189478095
1517721535136511891848921

291170326272520237036141331

222104319651529172822771298

20599315871444137119841125

304333050393831368554581268

21062111851180110912761449

1769711859184816282490703

3111240
864787647296611071236

53316522471404630140

3016124125725543796

3920133972159322134339822773921702
591223534013496738842803215

496193028753486266336793800

16870769310962447854745

1487233643283431712645628731

172371491643531756477

1908711558155315312148636

233121920501992200625251664

17081986598151461155788

1249081483150714352089508

161971

1408972109221820993498495

14911031734173016012186642

1685189349147971029505
22288468138671294912182175915121

21111111719163314922458542

63310601802159115142143618

22513302330224719453224613

95296382512399552300

1488871508143013011913516

1277411190110510651526387

153426463554598727339

151497707647633808467

21313842604212422152955564

104218228192223256270

1149961475152213451960408
0000000

1149961475152213451960408

17712331640163714382184575
0000000

17712331640163714382184575

22151727412118921381191562533510858
390781172236086158358535577337527310

16421516191310

2338103316149

83280430482406653100

25602854283049

476261617652473573410

2135901827442226

7424837439535452390

1

31112625171930

552185346497166

67452528323029

35213463284113102302

25452721243962

1667041278121410901754313

25613582988250422973498488

3276949769018391252728

147211232019

1

4822534630729441298

453793045223690

71710711919

102114219218210181158

858326542194681727916544231484806

5114117820419023560

67191225152039

16343319916

145219136153190175145

2675849209778581162331

27810492266177416662149241

26666463535958

48173087199891511991309

1049114914451591146617081762

193752013715

1184397937506481093202

1461651078286117111

51251224212923

184960552634240923473508931

44975110671237109916252486

2467991431139212621686389

97694462634871

8155159145

168424676648576798355

5400335935447551152480136942017076
12287716119831158210857152773982

3616124726922428395

2217731926730234298

1679581836155914842229652

22117502653245924333472623

19113421799182616092352615

474384771556226602290551332

103727117210809771579344

15484310309718841335468

21414412586226621073284507

12454527465431

1669061395142912261719506

17635571566867

84380514580554760481

1759301840156013912149776

33162170126159203192

25316062847251024433704607

20914462767271024213687611

144580823862757986477

2810410522390167117

315111215261435161919501035

17011451526169115272304600

19432752446537

95428523505463589391

444332558435480498873731254

25014562542223022182867746

11255851425322

1658751089110510701514410

201010428181191720416235238425858
8013353386194857607539987986618958

21115213042275524304390514

1274797997677411001368

16710091915177016672359501

3811397139110132165

4114529624620836876

314199930092766246938471003

1

47223255268275340133

2702322352615190510068713746

31167199238232294149

1067979539848891212489

17614372767237322563510463

836551594136413591783353

825497626947271103191

401300047594337421161371155

74313460502425631208

39301686623604816104

55380463573446843144

77342432534363563321

28596341446361

896841849155414172126240

17812612323200119773170397

1609211511146413072076338

80629114110159591416134

1038931596146713161933281

19202419201521

1429821819167915262263498

754248367636831052213

13961311816

1228922026171716772365288

44151240268236302139

32246770571597785106

26336690686261

13310231810161814132188223

0001000
1015809169187521216197

665018127716631093146

35791041468912351

862013321733

0000000
7303818393825

7303818393825

74265169

0000000
20999132122163198279

271061133
20999132122163198279

19897142319
0000000

19897142319

0000000
1813108211120

1813108211120

17071103101127151237
8912312520

103916221322

102712212323

38162621224661

271671061524

59152528283968

18101711161019

0000000
1157151498

1157151498
0000000

0000000
1157151498

1157151498

39262574494519704101
0000000

0000000
39262574494519704101

39262574494519704101
0000000

39262574494519704101

2211616416114318541
0000000

2211616416114318541

17937118130001114510516146504104
9163126262223

99131315922
863191441763253317441951847

0000000
14262221202555

14262221202555

6434380373762191298
0000000

6434380373762191298

0200210
5251012859919771436180

3015555348048166079

22353732511494775101

25497382616954
0000000

25497382616954

0000000
4928844130832444281

4928844130832444281

16576211119148119260
55251211428038178781125

54161400232227310120

862788715443122

599616276109116222

8675119163166114230

305690896414192

72217253493579

0000000
34404229224142

34404229224142

0000102
64137355269317383190

176725320423130469

8112817232535

2032308203624

19274440421860

609321247994461408659191435
0000000

14310271757158414622160329
609321247994461408659191435

81007070488545

3113112612811814348

1678119796310572

5111910481768993

554987698277981179132

12195949424434

9312735312819

11182925341316

31888571979057

3313413114211617790

3616126527523936187

3115826525619729883

1

12264346135029

4127640537533654895

10277321484242

1896333273918

6294449544826

2714515715211619186

2813820816317122834

2220375
22416563497291728013925549

0000000
2622168744852870854

2622168744852870854

0510103
58328631536419662180

37278583498377601113

21454738416164

2116249638837151364
0000000

2116249638837151364

9312732414317
0000000

9312732414317

1089121654151314391992229
0000000

1089121654151314391992229

13101141
88320497488429589250

0000000
11175636342133

11175636342133

135110149806845
0000000

135110149806845

63249339403314486171
3396860577510

14192732164639

1813315125317925083

285893586211539

0223248
17142619212529

12191010139

5111569812

0000000
95333565613483737542

0000000
95333565613483737542

95333565613483737542
0000000

95333565613483737542

115260234261192344263
0000000

0000000
115260234261192344263

0000000
115260234261192344263

115260234261192344263

0000200
7928713116

6123

332624104

413412

1197154720901745188019552045
0052572

8162619111417
189350532434398485319

3514721219819921395
0051653

2312117417116217156

12263326313736

469286977810880
0000403

91981351119

55313598

21343938325424

10293132242624

1551882

26308140705148

351583220
746512780409979

42136835207032

29474437172727

1008119715531309147714631724
4292223282646

9233419141750
0000000

9233419141750

995114514971267143514201628
33102961248369157

2174727262545

34202125283731

18153020191539

15263224165626

2363138977410336

33635485115632223

12292224213845

121610360908041

12204822341530

19181629283235

13102725224436

191133144404462

12392232132231

151510748

1346258344535

9373436235245

15264329305036

2267105107758750

32272920323351

17205926705248

15767583913862156

165157263643101

107245381339317369217

13312523231144

201945705921607015422139591721513913
182709413316292322216

967213238274191382541941371
19142114121413

0000000
14173724442735

14173724442735

13201811474225
0000000

13201811474225

1592427402420
60229517423405527104

932307162224

810142723717

1813640331429242222

4382345234112

6423311119

0000000
25423034342740

25423034342740

14443242393635
0000000

14443242393635

0000000
19102720142418

19102720142418

4247381281181916051456481
73216493509452640155

3202917301612

16413248612

414724

186264373893796401100

216132

7651681491192008

6462525206

961518221121

4221914171614

34475768644643

131

12385122253754

64223340233332

45202510139

3522106

137222

28224241276202235103
0000000

28224241276202235103

5211121623525927398
0010113

14111925182318

11162410222028

278417220021822949

33425438383572
0000001

21272521291830

1215291791741

0616241145
132267743598600831179

18212627203226

17141714315034

16254120202219

3992621152025

4219261749250370370

116347577627491652136
2000030

28319295589423

4519828126323331540

7262727161524

349217724218422549

18273330351532
0000000

18273330351532

452044263242102
0000000

0030034
452044263242102

16101013112551

29103113211447

932340031
1150956468172806703595182073445

1147356432672366680593181753412
725242478821981826160219821321

14452319232126

5827753527262820

3324940036836153051

16122227613

23201918202721

22412024344522

185053433185228

24111541272728

19025633

10394141231115

146092748122714351207

3013721112233213

1214415016414422633

13502324191624

47865112

1547820152324

198426019220628035

1510611513710014040

1869241128

3118130333121841249

633791552

13293635384623

752215201185

352511210101221

51069198

6262531304319

13565819235324

6542430262116

4971714121911

12281323163118

6272125724127428539

1111470695711515

1718811916411817330

32371411222037

8544441292930

83492091416

432531

6463537324611

112383833221639

4334446735031449469

222196771910

8102389586627497701118

36170717841837

12614326154333

421112456

12655064325223443

7112722301525

25250912920219

12375745529519

4754466799410465

4251618162819

922299111130

15309637518120

12591128131519

2025554

24201418212329

45769

2913197610109

10272325214924

141303823243714

5621821525824831840

14617744143468258

183122615

61513521618

7643047474135

137513618152215

1951241191415

1675621016010148

3011020517118122945

12155640424910

617112823316

25291033442220

645044104727

11273041334526

2613219517315224636

1218414918012226668

0000000
27324023202932

141730971724

1315101413128

892515111020
424559938783848883686

50103130
129177224191201225234

17151616231917

15101520261614

18202427291931

9312129333133

9163013193124

18182220181837

14272544164722

1039396122024

1412213242132

0000000
1362923292126

1362923292126

0000000
1991618221019

1991618221019

6915117109
59263437338366404134

1713132924627429648

23785046436236

13454335423641

19695207198219213253
71218116911

3852714123130

1881523201336

2242117162223

1461914351818

26201117272537

14113824242525

2091524333727

37204354463346

109871158578117112
0000000

22193224262340
109871158578117112

10110711713

14141823102216

15131613142216

3454844

4236341483823

311

170586125010019831227287
1002020

1217341625720
0000000

1217341625720

6373628172710
15756912169839581218267

13313041404313

22474033142625

1314237126731640538

9132515262112

881212181515

47248595459456586106

162988105568235

23141923151313

100406734592604837226
0000000

100406734592604837226
00100242

3114513715615518649

319072755910753

8304236331428

3014147332535552694

4001208
6688141214691725134613867468

0000000
1572329122632

1572329122632

30214723274731
0110000

1813368123420

1271015151311

41114094556214334043969
6639138413991672130513137397

1110458263

11173939353527

3431120

4935855

11

81436126

105171181623

3223293029

23441463

12

39986046366178

6215125176145

13423111

13513222

201859214

124423912

272111

23342

15185310124

4913122381613252

221124614

15556673749245

3012131441423

47735444568368

18617414173611183

1320311718329

57195331055

187239611

2142

22393027173223

6012179202525

14121347

57716101516

34527138355438

381426

1912169161520

217728213549

811823

33131712101733

6315821150

131666826

10202635192432

831251016547

4763298

139114104

111310314

24246140225639

81

1113512

21319418134

67152220344722

21142819213633

912565

3184335

34203927373731

37368356812

1874224181420

921152

3228

1134360

215

35826112061246

64147121422

53636514

23132910241927

7813484
000000

000000
7813484

7813484

670294349584317403561221235
0000000

670294349584317403561221235
15233141314911

944746827446531028264
0000000

944746827446531028264

0000000
83336478428399582198

83336478428399582198

47821103767310429524463762
0000000

47821103767310429524463762

175495516907286278891662492519722766031325972384488961
2608225050338853938583133150845567168575

12741100006714875613599912423717820429522
5661086768496856636783571206

194160747783515340945363303
0000000

0000000
194160747783515340945363303

194160747783515340945363303

18152451797180114322091391
228546784244462276720376294095285

133023265165491463413614196633086
1142535124111969911618274

6409730912278117455105691477

17829931764166516352317359

15821432022176915152410405

24058642400219320182749571

5051336236963947329245811238
0000000

5051336236963947329245811238

0000000
26949122404238520383074570

26949122404238520383074570

18915832307213220102788483
5474461127824771677657419523712889

244220096373493359231027450555702
21818482830267123133747575

6645911135001141210831153591624

1028371807155814232029307

37326984655437140565897639

33227834443417637615542851

28725024195403436965659681

36827174743416638775269766

988001176120410701553259

0000000
15813602450240321982979414

15813602450240321982979414

0000000
16213201881194216232246441

16213201881194216232246441

281367164
478428966546509576283431048

22219463284303827073820455

25423353357346530484507589

26120973447316128603986671
0000000

26120973447316128603986671

69600830695661872157
178415367241592193820261298404130

42436145907535850937507988

32525664564402637825383698

35737005619496845816866939

28624973798349130694690661

32323903441340030754522687

4222894697344363118428344406656839
58413857959251167399

33128284984466743456227766
0000000

33128284984466743456227766

25138756121050095538374122262293
896450861432395308534430

5542298168257176561023211

952247124211019581327271

543528973466905602787211132

425187399655435425621249

252365833282726267937281235
0000000

252365833282726267937281235

0000000
15611781609154814331982383

15611781609154814331982383

989011524151113961791261
9127283134361209811002158292063

24019563272319028083906588

29024144599404835695570604

28420124041334932294562610

0000000
5242614169799691124117

0000000
5242614169799691124117

0000000
5242614169799691124117

5242614169799691124117

578416410
61607269815673

515188101311

611126195

5643618

1161914

21411106119

817244128816

613485497368725288707427509041068657168981
166797615470445271308452419581522111336317038094324564

21037418852503352416319942028132514074738493763
143290013116391247057312116965619790968287653753630981

442751410649592387707747022729119710349114924856
825217608811550917136699012599221836715186856

1109510285621714318093117151825606920341

369134050690515866755590820798293

81167537415245212734812110518282315354

51904818897175807867835611475410588

208319597429663608434170505246052

241823089467903999238295566996737

721696724341773111145622613730861866391125710

453341315843177048166679994778963

276426966656575230751394718025385

4586433679074074739720731047329095

530551492102552857388162012470410568

447541782103772848648142111639310013

379534857734936118558259859227380

87218222618801615347414720220974216437

387036019861476920066607947097609

91788573918412115193714496621632317482

520049041103744875278299412387810423

46284346688636746187131610263614251

540452284111194917788839812955611268

2483122713738614131504129903142126178398

224220527414693587133782496454922

569552054112184942118936013165510840

400738138798096682463652964689561

553952121100460855668039312314611323

424339667816496777765019963559146

178816169323732776926158401653608

418839275785716756063246958798207

50344744598898834447913111876910098

265926009569474522644379646725562

102289518819604216262715552223516318159

281125559539064517342926636855749

1041099550772380826198949418612832525791206804

50484774199068824047788911686611172

531549198102340850458068011996510729

527250167106093881188377512370111773

6607609781251901065049970614470512605
2460322949043165536638834534150516947876

8657390143821230111907177651608

122610528204331818816904248712234

181117150360023049829053418303303

666301397105210451474126

150714223310772571024439347103142

56852131130597148846124111067

383636936352572999428451422157042

124511358221281877517915262302794

37236337704691263919157862

474430287947259689598991100

231122103462363880536271549434294

152014928328342762425816380723319

8197531137761185611248167771695

137612587251402119620454301102685

0000000
204519515403513421931959467434098

204519515403513421931959467434098

1212411449517655115500914411621373753169
44882427532592975509768479192711647329173

7987648136061177111044165363318

59475639937330304323018843310190329

704172580228320903028149

153013644239722003319036276923597

1134104331881916767153892337311775

129611383199031709615808236115432

68061281196299869143137911959

12313382275192417842710633

32930114884410239526113691

40633376444515449177563921

737471335120810561609243

9368689159521413412726198202328

9310032458180117692448271

175615533282262443322981353764343

235124005148521261611816178307721

20416522910254724783376567

372838694307302627624666371789501

5515561198147169888662269713512713

349632017597085047447712716658836

56350161087588368464123391970

173015824261322299821831314078707

0000000
50388688580612811144

50388688580612811144

11210532828235622983362248
1715115938931910027615125939838621032524

93788778817362615075314061021017218160

76617054814264612304211649017267614116

3534233245367298957925154431979943369730
97719265718592016216615226622095719788

380395395907890753511049682

398537409770676625261530908207898

122811430219091964918084272812708

113810799207181792516959248472267

360532729686935845555075816566823

11511152315205118212775331

346032125641475479551698750036424

10179580191701668615664225602095

4497428548555173063686401034139020

434640948821657075666395972368374

180016854357442956328652418363320

381832212616333334543084907
0000000

381832212616333334543084907

17018565034358937284556350
427395510430774778309649803

17214284001294231113470266

8204730244939

4235461457743473096

3529773460953384452

20921135919459444995197430
66611112010711019

15915734744342935553979266

2230082779765385490

810416816312715626

14706985579829

31910828995634653424405513337706615595902882087
6278285748642961244980697427660385113964741674252

1119310301122130418075317566226083920785

54364966289425724506970010456215056

8988270172311361213609206381790

279525146450003624135334527066607

32959299666377984302402295036439687113787

26823654403380237055474632

2456622945346886938145836785055301845921

338831561544724378942421636029031

26727705417435842175932700

737664712756100969731142791533

88678215813534711056810654515897824684

2648724110540858033093232172848157169276

27624904380349435885033775

99599055814902212135311796617311426957

4410409676918055594536477954912009

235720785351902943327833423815890

132112059206391670916276235663517

304127480525584285341811577147084

350132478581684742245322669079022

90578222113270310754010444015741723946

16363325234

1942018190636990829808928848443085739492

189518130346812729426277392944718

128312350192881603015174221513906

763273513116624942129171413692021434

23420162534217320622832685

34276151142150220141

697264152101266827348131012033019318

1224211528823268819008218112227019725196

289926606428313532733597515627926

23728115014406240125787703

4930456049120774870721981100769442

749569977122766968679528514171319636

592655533118495957779231513845311279

49704685794577759107400710934310774

4738448937028656534550808171214314

1238511266614316811603811124816769242708

110910996219311780417293260332448

73926930213037110594710238815133116698

255122958346452796827314407427782

71863451018182947896115652189

30333596272507747417904706

7128662101261881019339874414089118351

1675215383925747720897120366830797043612

19219193862316430574691385

6196024146081113610810148951139

67261291004379548163117571772

19322017399

88358050913621010830410654315687823884

14516222505230219983512324

42440088101671064399152964

7125664921263951012889857414753716044

14812402674220019602936253

9708824201781628315563221201818

62175419685657713056798710392316595

234421652335472691626088405676514

0000000
365934125836836659063780829365907

365934125836836659063780829365907

5113462179117581279759141087349887
1733315164830992128149826237236365532782

217115891274072706823379337204075

327126697600815053748819717406124

120510560202541938017752241852359

8947934155611379813181184681770

2025455436738748251

35732215932483848777624727

12491011699616313

325351032745755107862

35455638573564644113

264624954602704757246582652654606

157714881269163517230066315522995

340540116917319371117196
0000000

340540116917319371117196

397328579536289591079293442
2088116353060021622224422939813540

30155482215306402146

253118304433

1735189629111682

11641151129812865

31201629506459675382

3315242512422

108722917214824196

31149515322339499197

5501597110612693

25446062624867

1151135659596107

1350112569213278

9478660788656

12116168129150170139

31321723543499752121

2115640736033043096

1614626221925938298

5606854569961

1731922242325

24146781

20119398292276421166

27363285229180252138

352938765735101021450

34353220227182234442

29377219267245229432

765189108149195141

84911276718798

9170440279276478197

12561199611717479

9356555495227

9151241289241341204

12110311190261227113

4412

14132035223020

133320192512

988083923235324223237612

1007871482120511921629629

1644514

5335437372710

658223483294607443560

13

121031788910113167

125222715720528599

21123235157186304191

525231190748886986576

2127536434534

2624957840041855970

5056037642357268130571921

4644511077388221154400

35275837563598803137

17591389410413468

531533116

1280199154201249125

136716911114416192

0000000
594326350134238423061371927

594326350134238423061371927

15514859376221
0000000

15514859376221

0000000
541320252767643452069574574

541320252767643452069574574

60681018726192456848515787561511439
54417644852515703461826421846621181105214

2085321843815392413779412398318358636059
470462419326462986926873391838003

42188427627225129995

1663212182938

751741376356889624489721293

42454334778400036726101819

950911984237430663599091935

4651752063425828542776511030

9338478863381297175107402796

10297650814072176673101942055

9259080839777096829104811702

1300674961005448513874961822

762215868730525902115

35398503309283826534117621

3253841313952114469868143434285

906105321075097659117125611490

499880066915731518979351017

940771549

51366255370502945087023903

116014273147431296011952176482128

1084499412731433109216732228

14611688855705043413963251675

41538176487560550548053840
0000000

41538176487560550548053840

335333058575241504068811161
246223808403083497433134471998742

9809138165991412113400191613643

490495371576350590087981686

65763871069592628794123592252

7516688795973306887101811553
0000000

7516688795973306887101811553

12710821914173215922203321
0000000

12710821914173215922203321

918148141040691628264124741992
0000000

918148141040691628264124741992

400739541583335269548472702119117
69371891068897098617126531593

37534554669430942155783754

425404154445439494575371023

4975248830174206751102471183

41835565852509247486552985

44343807187606557558285937

36934094082400934265001764

42543695784527448556975973

36238946326537851607178905

1039792154167515892168748
59854361038778908328101903516

62532920606743821390

50543992455632731591

12012542118162316902082679

17113552621235324333047537

927731582117812411341571

184819741224521972118353261833663
5502572746488958198541397834911616

9911919008517891088266

22224052767261123953559526

37841934858429140046000769

693660065686191557081501797

1

43344455938552950767568852

12515351635146614751865362

134740473487605617272

21118052159207820132865360

23021862142195818362673378

65325729493559777154

19718882204222919322831422

14313371991196317692785332

72488821007383307763113881463

740712911165103149127140191503
0000000

740712911165103149127140191503

11711341890168216112296417
0000000

11711341890168216112296417

590462036191791702414482229377183
1301307406364356403150

132611717838675756334104352005

4448490121038790857792120995028

10211903510964101189237140941880
58161006986495158669534038157610558

529876370836233557387851002

77211324855778467293112381323

22731442762252522243247429

50777915445486843456831892

40572034374417137035539833

87023707949780777381111541444

20929042402215719823076427

680857779366821647097171209

596825059315853519578951119

13910851987190917922292358
311133319338

554958768758061150145

36329633578602671100

45250465423365438105

26451823
8395082819870781301598583183

5933631659655341140378511856

244144515981539159420051324

151559761915326413573312948418222448495
634492769406230623778562882

51342471477387649155
345112448200141907616562232358712

433366059505361517674541420

2331437572596968515970654342

636407163346270584080672795

822527974406828617692753451
0000000

822527974406828617692753451

8765581909484117691108742865
374235138893590340447601096

301211538213400292342361066

20111151384142113641878703

8746432866578227214105933215
3501228303039

500325938943686339450081228

371312347594108379055551948

2302529262966
187514181230992029519380281996411

6875485868876967532107262297

11868666143861257011822174444048

252193017781571158919251079
4619356685672648256478076610514990

380292439703629340149121037

445232330202235245628931446

33028141125792999851117841866

98812117711109821525384

614472862645379511978361719

535434863175519525073911855

77265411025088298556124381874

522367240723541349852791631

27721603029255725713603880

394341655924587453465191219

573241935913218339041981529
0000000

573241935913218339041981529

6245153976085998334122222011
0000000

6245153976085998334122222011

0000000
20616512039167316832207744

20616512039167316832207744

601388058965325501074601685
0000000

601388058965325501074601685

695510163335985522379962175
37250449386345561136

317232622942274200030751157

34125253590332528784360882

8079809796675474431781288023243007
758138802755677411267959749005677815309258

196212223189761636817131218084823
10712861289114311621666508

25222283802330632074394735

14947898129381097711960146523082

1098119479428021096498

12349102145421269312592175984086
353269437873313326144601349

131

504362666635726574280801377

64392705663682881284

313239033872991290641741073

2

135910324154171361812887179874466
0000000

135910324154171361812887179874466

255220045221472087422481274469573
667413446614660510558592275

265252929512698255436471016

56254377385539426209

472385012525780158

32249228267216313139

65413453488458581311

298247139783356330248061070

322372945314011387154451050

38189120126179154196

272205720991899185225851343

369305817451790166523531484

875668258741973982178

34158129195510215144

22618962050182217812605910
0000000

22618962050182217812605910

902775034463380282640131814
59320152132103161151

62655711342142111181540891

21718591952182716052312772

76560731028185648447118852296
5752020362768

521404180696551637890521283

23919572192199320332806945

87661117710559571410248
0000000

87661117710559571410248

5591381385809051756493777050619527
11397078977090228428124844339

17013402094182018342758753

6135270839375517122110291883

163862109510649821351743

319243438313547312543831211

341214431352858262839471452

297223236343139317640991121

400291343003824377151071348

149821587585648747740

15813411847156715922096752

515375665565047514677441557

19715002737226721312899650

447159723212285211324871015

20517112404229020293283673

478313953864890465260921290

0000000
259213232422786284433641114

259213232422786284433641114

24552933683671162488109101903624
1403426697456359691193519596834819204

18313981354186013571770618

374287441774071364053641119

15914631987217417652235572

373163853062296825753643778

16111741682213014872001402

393673831466177175

24218462294221022112551642

22247121646112854

15513271100120211521400680

579116766942245004409850612261

14912421926170416002288638

20919852167188316702424629

26020653130253427324129761

699693827762936265336392310

884146105676519402535567781492

12381040431491305426843372896

14615533397286626243705273

495409157325737556975991380

0000000
346307736263230317343881562

346307736263230317343881562

0000000
555576259296050535678612411

555576259296050535678612411

867737993396087670489728104
69570493349428493793

545647234305197341521

12593311799679751265918

265231536502137220331291687

217179119571315149220121868

13712061337889108713391317

946893163232622983082671
0000000

946893163232622983082671

6190949602226219306190862751612659
4329659091142997689470141084550

3284125104

6452474237324

20416578677897341133260

10561998345093865403454316372

4064138247425846

86146211438759731272162

466482842253708379154721261

2571224453639629479297913942712573
13810751733147115251807819

918581404127311701549585

39424656485577741189

252220746903339348544411786

177144432392206242528491193

399434923802044216527431447

410340056275028479265271451

12897725411984197127501032

482390361515312517976441639

48313411296330440178

979271908142315301900607

816982805204221052717580

229187028512576253733191067

1561194863767838717601416216407
133151772493884614346227221543929110121

103110

390235362507783768836421850551

112512

274972970200890933204025263810656

18920492303226423572715886

32423232492278523773272922

529799224287099970129213484

355316619542692275723732206

882800074877036803185565083

12611111017117712281264523

621774126753626439830854730

462507921082583350321804536

1383975432524177526936869196

22122122278232521262847931

362329552394565453465441168
0000000

362329552394565453465441168

147610128147821545115015179805730
1057251297114710941540348

25512941970370841182428746

320281940563767346950341486

341213127142638235731311512

455315947454191397758471638

3686276594199637450351635152413255
124539407715086513475612503518527240803

289178422972163215730921091

518380155575103463772511585

7455767945587918081114482461

17315572082191617872729607

48166909521726

496403967145642560680671464

304253437763222293546441184

366293751114113405660341175

3694284834817943189394225895511133

305255833913366285146601183

18113752283203419622808606

352231936433259341945501167

637423469426536579686291773

15822123203812

505377972916035538380501543

18311611961182416652576538

534491748134707438856422388
0000000

534491748134707438856422388

402286243304008384856961364
0000000

402286243304008384856961364

13499829161861583813894205482688
2391815270127057024586522447433209843694

19315343111277925993436416
0000050

1117031183120611021488246

828311928157314971943170

9844667853780007096101431525
33515282504244920592953529

42120754171371333975028551

14626286396130

515751417

67322575640562787103

86323706617568782171

34651064

53352508483456512120

041011652
2311915614912611652

171011311191109535

6141519101615

0010010
6365055993385838064116411283

15311302823227620402980256

46938297003622259368564972

149610685889655

136301625505446566148
0025243

10812619014811616565

2817543335232839780

2207226203
24722734013370233985029782

79657106011239201396249

16615962946255724723613530

0000000
3012529125322329571

3012529125322329571

0000000
828241536145313981829211

828241536145313981829211

132231201216200147135
111011784

119991141241147297

121317781796734

10116442
692631110886101309232134391833

436423170426576588888521179

25520803833354833404583652

20611612775237922663107471
6201010558

76308854622613798151

765801544131712851809173

48253367430363495139

344758466263183
0000000

344758466263183

131246992912326811000510124715061620074
554125600439453998836817538137366

67121604298382634235270754

816598911572102749517142941754

42272

28722744257356936215023531

906661280116910871584189

38660311329078211238231

104450660752592898146

28923024204384033345346519

174810539162561375812723191572994

6384063794573766678100391178

34230784012380833115114781

29520103211316427334303733

61817013058269224783951592

1108462053176916062314208

7184318856575647052106901133

47133266818554954527575963

0000000
21317812770252622643395470

21317812770252622643395470

0000000
17715372657246921572883393

17715372657246921572883393

10258924154611420512995198702489
53984487279993731806668910023212279

2726263455952766776

41237086978623355538809972

14210421946178416782462377

1713918319718223365

13510211832176215602368294

59658110811029321547117

21416192415238921903425565

17812792229210517772712407

38331165392498945516917847

28923953992364535035005709

19419273383295826674013521

35728624669456842306548816

431385771426988613590031011

31826775178433241515694705

5784868960483587704114211080

36723974107377235585005657

27221213740323427964533571

0000000
26221053574365231134613680

26221053574365231134613680

786669911177104619692138872167
30325524081377935065253808

17615162740265323573359507

807601524127312681828226

22718712832275625613447626

2616671411
553261149334562409058662530

490221442633930356952052381
3413843513830

1414709349947591204175

16613702564235121673266300

1803337275425926971876

0000000
16335859656534

16335859656534

45358596567449582104
0000000

45358596567449582104

17427113
14171623173630

7771661412

635541115

17117303215281427143764603
0000000

0000000
17117303215281427143764603

17117303215281427143764603

227612817114511433
0000000

227612817114511433

24717233111277926163659703
4124410240214653214074413742217401147185

33597312843354740279444043781613022
4373438117353080261217281047

179111631607156316491791747
1923211818234

992373519490595600195

26454845402669

30325543777995

57260520439407554157

667430444528512530197

0000000
10764712549838981440246

10764712549838981440246

8221495416633291
0000000

8221495416633291

62363211
1777381158109710111440359

77254354351352547141

94482801740656891207

644801711123763553
0000000

644801711123763553

2378215614124851914523649127637051
8754536840316633807836991921

60234553562468598140

4441979510210896

150316448457408495133

2384945931542824

63358585692558879151

214786271205627

60768911389118154

2672762371864267864255

404488771009985

143481554147413

59281481511408650148

440779114599910921273205

163676566311682726723316

41866494805167

3810612713813714474

13612999861227537

32279701683731337

1134772553158115

1048594863033502394

39302537443453

54368604588536770122

27541711232196977359270

108208233322337301115

7324324258542

25565341413478

928912212414711665

175514521223966754591

16503469259955581239

372253444569372

324526545933142

30951993590712221197216

2718439792151023

40303242403458

294440454848204

131408160847593

112306573563464744776

24120333560351332164507883
31548147253377

683734

15715752685260723813426613

247214

513987868527961043175

649871109614135
74148182239191264234

27294039426999

417055905354100

0000000
709461731962872046

709461731962872046

0000000
523058526080125

523058526080125

877318258517508328272
7991840352029

171907015414011046

627219170323333198197

0000000
505413512212815887

505413512212815887

56554441061794008302129261176
0000000

56554441061794008302129261176

10012453
73152205206193276105

1712916316214819346

46234142417856

01063918
5759302877374486925103181549

0000000
24021044299366534595185742

24021044299366534595185742

15610761779137913222052368
0000000

15610761779137913222052368

17961122689240121353080431
0000000

17961122689240121353080431

12211261892170715792290861
4585411076514858419540317975226788

8968284142651360512233182622471
239821424382063431731318473216190

137317418817620392

14015892722238521483066433

28224314759412537325701623

62610124510879441528181

14812051993182516632512360

16613301997193515972374425

22618583749304929104494494

4636450145444974392

18814612785224021813295452

23122194016342432855143567

32426274255377934745084890
65996105

1389811579136712591899388

18016412667240322093175497

24122943954382435915367672
0000000

24122943954382435915367672

9739070903175047298993816966
170164113171235109516263469

195151625442074217328473163

158155728131998199427225432

114121515701491127718392116

33631417877067599042786

527456678107288677197521209
29617818716619915

13210942214184717722494301

22719683097302725823915444

16614082321222722513144449

52646122510079801116154
224018986359533181929446424665123

0000000
1109601859181516582156371

1109601859181516582156371

56424543502395656258
12321245

1911798909975123

36284443411294577130

19715993221281227463870528
0000000

19715993221281227463870528

959071750141514412000251
0000000

959071750141514412000251

51222156148
10863410109298441281228

3117313914315621984

724498497716821048136

3019746540241744955
60551301018591428299120581157

1008981661157914722052280

47540358059716164109557822

9158130152051336612245184081856
151356563624

18114923002247323333240332

733658712168108289849151061520

01447349
102556955831838921320

23173438294394332115

79369513530441585196

6592815606964762752576028044918259
805879409438281103318

561337259405442482969381265
4274544

65245387323327476117
25913312081208917222450611

56208239468213372112

4230844840437156385

34158392299286430107

13374035413947

244517015411815736

25330405406366413107

29820393852334931024484650
1711627523022426137

4120635524824329682

74518835688692103794

58461111610009521337172

5627549249436557077

52463779689626983188

0000000
6074664792677606821100491242

418363734588
6074664792677606821100491242

28921733501371332294618560

31424734389401035585373674

5935066829579757206103781510
0003001

0000000
20615002220225118982756530

20615002220225118982756530

38735666075572153087622979
1911915917917425028

21819583549332931284315550

15014892367221320063057401

1756793
431343352885179444062671224

24616953040274224533467708
0121891

91540530510515803277

13310322295209717752485331

2212221313415517099

0000000
10410911284135711721635310

10410911284135711721635310

8064095910748081156203
0000000

8064095910748081156203

1721128326024436998
72165931004394168436121521926

14113031822160516222464306
0000000

14113031822160516222464306

6194233303030
56227454363307434201

1851133937612574

3215727923720127997

0000000
10614332258204018692504283

10614332258204018692504283

544788778576951034204
0000000

544788778576951034204

8421228911511618
27221473387338729164047571

1106958299898241124223

15414102436230919772807330

0000000
757949629047831300263

757949629047831300263

28264223119141166437
9434390886235509646572366697

0000000
876659779319961210226

876659779319961210226

40589716710515195190
293402571134773819753964

10927177236142182117226

686781196135129130178

76402535391357411370

44182278209167219434
0000000

44182278209167219434

129482608539536688355
66273391357339449220

2310766988911170

401021518410812865

6394025442750
121369976479665884940

368777536871136

49161757330487708590

18446537422177

12383734245787

97151424836146
241168944272459314133163341

674671911109914401407855

165115124651318165318732340

265613937225922052818577263264077
2423258549148857367

135325874389394537075373879
0000000

135325874389394537075373879

21019473840306927963741471
0000000

21019473840306927963741471

514418876687136637791501160
66740124310839211440124

18715512578253821953102416

26118973847351532614608620

0000000
20119632502238221573079573

20119632502238221573079573

0000000
45263371311270295204

45263371311270295204

619311121016
30927573237319427824115723

14716351706161414042174358

15611031528156913661931349

1447261211116912241519354
0000000

1447261211116912241519354
0000000

0000000
1447261211116912241519354

1447261211116912241519354
0000000

1447261211116912241519354

28517722972265025703359759
0000000

0000000
28517722972265025703359759

0000000
28517722972265025703359759

28517722972265025703359759
1047991691156214391875249

2814367

1577120559113567

11281233326939

50286170215212230139

75475841648615919170

12733320432435

8781411134

81997891248849

0000000
228364342641856024

0000000
228364342641856024

0000000
228364342641856024

0000000
228364342641856024

228364342641856024
0000000

228364342641856024

134673126350732093879324317794
0000000

134553119350132073872323917786
1631473820

02851123
15810813299143120176

0100001
837976579585111

44505632524857
23353018352540

6514342

421356122

1181258713

0000000
39282025433753

1194310323

12571212238

1614910211122

0004401
75274837373362

60120180
25173523122221

71912410

1216142291011

0001000
50101310211140

81512110

421012491030

544457476361145
132813008335531013726311117590

0000000
79201211113140185148

79201211113140185148
1931137161527

941148425

8513451117

298217210

10122022172331

14106124556310217

610115998

51457384

610672119

0000000
11073135410061094145284212564

11073135410061094145284212564
4281021449515995514

17817153725

47393871107175157694

3061214151516

2062851311

2542912101841

133385

124451

316911154137

9314316

9563296

16122101626

7498742982884482

2

18612713530

167554419

363191921935

2122117123021

829782918714025102

244179461000

162298910

15181728

101247818

196214540991041796

8743813

3563323193055

5261134

73174538345939

4217118121351035

554439

1631721102532

383339223857108

271119820422

3191611710187

121210513914

38733233125410

28182215202045

9452717

1210297625

3016227111116

451175108236

37241928314149

15610711715

112894317

5244714

532164

9515517914

1921285912

94653214

17210163618

57264511

1076137817620460178

115816182418

48679280858689
0000000

7523316
34375545684563

182823161711

416512711

966211211

91177151013

466511811

14303735174126
0000000

14303735174126

0000000
257818718113817462

257818718113817462
0012001

7285250496413

1192216191321

741112113709727

1024560406062379
12126077705927736553501

3671582181732111561671
23211117256194

4292314148156

12144107667872403

2255126769

302520211116141

881721204031330

2319813166119

1216196136179

62448480

0013127
156851047097104198

52374129514974

37214120212825

34212910833

336199141759

504539264523169
379229209190243184969

31152526246100

503512372322187

258121115649

151018102372

451055622

3217129281698

1513181991759

114233522

26151412121280

3414136172741

24352862

3637159371636

2681415151932

0000000
37215627363652

33205118283445

4159827

10118277639
1716912392126113232

2313445261941

4092510272330

161331914816

2082117192132

518125182838

11710915836

516470648718751781719
02001014

505453635674740763554
2115931726625537769

4921101013916

3610816812

154751311

1114338714

5653428

173126151266289174181

145

14442101724

13211766218

4335

1421431321

265139141325

712833311

1277431114

7711691215

812935710

481522226

1616723923

1228622173

85991696

601032411825

396169131828

271053124

111513441018151
0000000

111513441018151

0000200
274187384276324327362

16146645617025
256167339244295299310

11616751319

22141010381414

131129201716

4525442927926

18182414161222

68611758

27171216162613

13315922311

6133210

25524107816

12128

9529516827

1155891317

7224131324844

2364638

2117129141619

34142314127

18204532272852
2082524

652615141321

1015111581327

12762748
0000000

0000000
12762748

0000000
12762748

0000000
12762748

12762748

0000000
27121719131623

0000000
27121719131623

27121719131623
0000000

27121719131623
0000000

27121719131623
0000000

27121719131623

569165330282862244638631211
13191260

46711371229889128
0000000

0000000
32438468716198

0000000
32438468716198

9102317161413
32438468716198

23336151554785

2443003
14285354272830

6152440202217

6925117610

359414310988116119
0000000

0000000
359414310988116119

0002063
359414310988116119

13565743315553
0000000

13565743315553

0000000
753419192920

753419192920

15335245382643
0000000

15335245382643

1454568777746861080271
0000000

1454568777746861080271
0130237

116388780663610954171
0000000

1070904
116388780663610954171

58107891058214882

5728168455851980685

2967941117412393
0000000

2967941117412393
0210000

10293641276453

19365770475940

71264515476443729152
0000000

71264515476443729152
0000000

71264515476443729152
0000000

71264515476443729152
0000000

71264515476443729152

0000000
3911721117618117473

3911721117618117473
0000000

0000000
3911721117618117473

3911721117618117473
0000000

3911721117618117473

14273029326329
0000000

14273029326329
0000000

14273029326329
0000000

14273029326329
0000000

14273029326329

26127369679421
0000000

26127369679421

34476964597436
0000000

0000000
34476964597436

0000000
34476964597436

34476964597436
0000000

34476964597436

25451511408215580
0000000

25451511408215580
0000000

25451511408215580
0000000

0000000
25451511408215580

25451511408215580

0000000
1335178039027081283302

0000000
1335178039027081283302

1335178039027081283302
0000000

531175143
1335178039027081283302

62148198258168371144

66366594637535898155

2756736436195137072169680413971101574863513
14277243112071096124370214366

2016358101315

0000000
6043851520312

6043851520312

500899160357849568342401917297
594248181153

3662683374524405444247
0000000

3662683374524405444247
0001151

1762313003993964762448
25121319121614

1091952733623224332397

26989451729

16156917108

19037375243631798
1055223

17328172714151761

1691520274634

7150488423091983242527371851
4900002

15000016
6990155920781828221725301594

0000000
2016111030825

2016111030825

7045008
4144365544969136

3602852443766102

47159512326

6515146219721755212724411390
745137146166173163189

2972226195621101

631655950813065

563716175914749

714303504395423608130

223202025573466

2519561652613878924550

790258462372340585191

33254240282949

0000000
2638309111137

2638309111137

1563316231155208207255
010001307

0000000
10463730172935

10463730172935

0900023
441797344706788

171053217272245

27654127434340

216512104
6229994534512460

2513041720441926

35153027126530

40827647578765
0000000

40827647578765

0000000
4251439669282513545972711146

4251439669282513545972711146
20400227

741244132531125
39267101158157105376

73282562023124

234261220712093

519103343021

611167113

6081221441791123396587
1686788124

42137123510266184

1912915171333

1222680754655186

303937473123760

42544228137755342356
414943773683217651882814176

27853241531572637641623412

17227317291248

284125015068433923134

36423768810923730123

12519515134123535

76889198062240

71399124981528

246106937604810447313755637907972319567812
6556109669978115084292039

1018120131117119941936782075
2195862332272710317651025624958762252105

342448565653334316615
1064033

137111861762026219
0000000

137111861762026219

10419025024310052132
0000000

10419025024310052132

14264537614918
0000000

14264537614918

27324845324460
0043000

15253331253845

12711117615

503580705173147
0000000

503580705173147

0000000
9545078706936

9545078706936

32591031188895103
38749883183141723215792212376764

1186541233127211701550202
0000000

1186541233127211701550202

19164330191924
85561371026568105

12

18626

35225729222434

651331121125

2451810111414

113425661753588838272
0130025

13334543256232

3222637749437053395

20436175658726

10428256436146

38809385859368

90156218203208231122
0000000

90156218203208231122

29707253136421249011514155365099
4398771484144913561690804

80216488396432583136

2115051739735949241

19393146434745

41234724384948

22688712093124132

12154228234415

61312171977

3710016113220016850

109210582808831

31243241462624

205317215310918735

1

13111413101422

29265060297157

58631

15331535122323

19334858465439

277170958612262

1

23253545495327

23152724402426

132686710

1641418411120

1511620020516923242

159211951217

29172834293218

8103951343733

17134931222832

1543

144

3115931027122735066

11353236274534

14102320111811

64521127

112121414186

15494758487524

441254

10771210138

36431024

17103921131327

5416720623917724161

9352021413318

16212520341519

16273720202746

112156201616

20614545669168

7353445272824

25176330484436

17144840355428

1712211314613

1431416143

31334438815123

19408459666948

26332731132723

15133932373817

30137455724033

8225254436731

28204023271537

14163549395214

91017123243227

652113141212

24304039193517

13163943354134

2281620132112

216810014013520041

21414336225016

2192010172

25

217218413513823186

12225525176116

2712940233830846565

1313435619

41122424421823

66419827700663985127

14341917133224

123253517318

57248461406420570109

75123455

17183929341923

123582697810044

9162026202424

7846310689128271008161

4321263

882919321517

43101354

4315319227721435977

3311920722718924761

161439129

12813311114

3711910292914

9245161817

336498639013060

9292224262321

9405242336611

161224758

31456653364134

47248738621574805104

7651012159

3513735233227940853

1912519716716124435

4222037931834551480

3711149209

123392101809849

29332552221632

9244755496928

12504961455413

309920121520127577

9121353192822

36861361049212858

19164961295327

754518213412014260

19122731142812

14193223143327

13263768

1461513221010

15191919161630

33444039413643

19183637226315

34196462346309541131

29159345259235385101

188623421618729744

33193129403847

10275427323125

10482225231326

73343411385332545131

21

46612802320229421592919861
111361671604577925204

63101417910

33444154626361

1163276

10522770737211

87614211713713970

14757613010913635

25296352429245

1117473

15234339324638

9381115158

122412725721671882198

10145769608140

536171713

299115017212016650

37591401098613334

283613410812411831

11051131

23383

183442168244348458921315
214134124813427759287295329416513220975

310571535210650125
185628315

2823196514

8912331030736

3614715281816

1

4717189131121

851218718620

7733

57610411615811161215
80213014

1843659133432475

1710371423

3675852175135123

0000000
15366118225796624

15366118225796624

67990476925380733966993143722857
211118924435424590241285182615727214607

753112257201137

1763141414231029

5164013161516474

14361305453418675

26322285265542341173594

236231837247107569141059

50745173038031321088

2969327792383415312823241

3203539111637

109573083125532484369454678

28272453425442088

1533

18112

207218982641044

26665606895097453613524

478896419423211120931

3423211272130133

3

21131601245979260148

27938451157

389304131725

428532827221327

3798394818472604944920292172807

5939281514955

284411018325

79872811386117

8100773252019157572732220672

2533238247

575247210313152181137

213

144142

395113

1342167179223551

4844417484464311054

2124122

60560245181616

103211344315

430276617524

472662939372966

140512631525830

20970539255206923082798109063216

21

392362374532119

8

126

22124159110616200272398

2174215311521

312441123141725

3031343472042207610510

429279371867494701323264682

61195992436210453157

125610623183532379

334211251877

4634444981311420135583027452

722861017202331

416039412240241230

26211

469359919316

1389123812121357

5537820297261122

356602114116

39473282013281241

1167911032656895191

136182

326373632126

224214181097

1102521134161839733787927

1679174913131470

43010973974971401819
1113212441

693433436441

3507467919591321737

493811617171
92319912582252771902670

271223932474543246

11412108222232

153292714141624

3105311655733292040

24322761107913168248

22243131415119

110828522072619340187200
45277311271913

4015658946181962

5351541774124428112486

12748302317342539

2314495591844371014245116081092
2156111833632

1035313210231378227069
2110356311

301127214621716

39178136201724

3214979861361773528

6203335
7166431041474554

48252776821819

17391625622430

0000000
221522192112513

221522192112513

26723241041827
70464493

62343598

13516142516

27412323943635219392
1373715117620651797956485

38334852864112

1223058169912835

2230146455820

37142454502127

198575947724020

2523517531119

26386328551713

18183123531514

1110961587

17844845201822023

883944971533136

32121213

9014930725623941555

26241829631715

38171144591221

32332848573641

1921227471016

68149118492011

60172911854125

50292315292734
1955054373972774

161651465

10662512681122

2314523313

514724242713330
0000000

8911399515

433813211722815

408485243403751298413321
643007528361972548

11010847501594946

19222116861211

2211101734821

21181113391812

157473136572

141042914457146

24332739445519

5163584

143216855139

19699611413614140

321331881231527

22298331222526

14527114

122116171011422

145520745624

44261921681817
0000000

44261921681817

6666105511857522994771601
0000000

3053632391046127
6666105511857522994771601

1478194601441859127

104161692617156

2840574954428236333989

8331119

530

192623512012931511153

1189472412018727957822225
30801902731441431391012

106534971221047370

5216315919619315254

353751134131772826

2528446463

271126863216

971613133100

975874917214529

8252815524434741

86482116181019

1573148101665349624

3273022578125

28141631245316

45161414263131

6582117153632

25127

2608202
407164737633171290642761395

13203124172130
0000000

13203124172130

89530139510689811417133
94951112

74504130010099071344105

6228654736226

4574514
27588021021824169225211115

8363022393031

9303732354130

562925322623

136153208320201264703

2611365453149669174

35276562476416724125

6262931442439

15154311151115

3122050337240970971

381216201221
28211235247214317115

1913612911610719766

667941158710828

7007104
39086886816784963242360

74225014773542
393171309191375258282

431219923216

27234629413538

1611611212921

13175963

2615151419208

23513214216

136661057210396104

26102322431230

97151325214

0000000
3508517372480120662074

3508517372480120662074

0000000
89101183254213275108

0000000
89101183254213275108

89101183254213275108

67564910139102085661682
14095177101406217946326571258312715

0000000
1152518432717

1152518432717

692124221315
0000000

692124221315

538618014616819543
1201000

368316613614717626

161149211917

1050111426
231104101160288104266

25132811412841

35212723501548

922520521022680

69402663813391

45141319432325
0000000

45141319432325

82517219111
94112113104867491

1618169121713

30182415161729

25243037241419

15272622252519

1192013171224
1349913411719497134

143434

2113307421613

16235742

2183125441721

64634763814870

0000000
26111915321817

26111915321817

183407725607725883136
1601200

88152413373135

4734063455559581259

21273318522124

26193420391918

64023643053693647335433
12436762728058276

53351103

37829152362021

1425602

84172213431462936

53155411

251210191162831

664558772695545

232115262501426

11132

23211

3524911116518

3013724638913

21211031181515

258271811789

292113691725

291318162101321

33233101171925

262081086730

3722157139621

43222232

4013408371719
0000000

4013408371719

1037517510016990237
16192019231537

221114

7448984912359174

341413643

844118161119

45272122281865
845132578324122

271392650542

1211295115

168170186139269140138
20031041

30223213551218

37163214181921

11222

20163828242615

10191013311513

18421015601613

21192018391928

29354433302729

469219348473917275347
443943431322034

5621105821128437

19127122746

3311116721133

703862861734547

14123425472219

1328102049

220847820933485162

0000000
904179691219177

29102129303329

15151617321225

46164223594623

5720935034332151861
0000000

5720935034332151861

29418936472358
0000000

29418936472358

613060100643289
41938922222325091751162

384625114662878

541546

22725554190526283857

52215473592380

161017151714

2023122436538

143192223203220156161
17121819271817

37796155663758

19415635311623

1824391826619

20222645325121

32142331382823

0000000
27366545813338

27366545813338

2229912562645
344190177160522153285

3274

2127271342532

32262119562040

308912451021

3944729511

61464131722963

1011533281344247

35353131841626

1975387861144755969458
3677921050176710811332926

12383369254748

915122015211

317911112714

1

9873724

16103

31312

151451821522

41251

1313712121217

5732152128143193

15287

950951189111523

510666310

2864

51692291713

23356595566795

18696222140403161172
70461415

33337428454122

3521168313340

40628261792231

3156344944630

40313728401844

68567235954568
2931103

11242614251916

55234320692649

54264268627493217622564
73151162543592164101

63465016884441

44295839606428

36241641621512477350394

5984075615271202545484
1919156471914

3011292535524

33191929662020

23202929613726

44261511872824

31252163511431

50173029923834

49192822613129

31403416753628

1421132044924

28191914643131

313214110411410918

19202619451829

40224738952827

29283229573630

18152410491715

42262135831634

67281828765346

18181916281936
0000000

18181916281936

831916201050
0000000

831916201050

0012200
22409671735929

9157135454114

13252434261815

0000000
121018514918

121018514918

903792761385964
1300000

2510277552119

41133329431421

23113240402424

0000000
34381615561934

34381615561934

0000000
30293328565321

30293328565321

0000000
2616723411416

2616723411416

1652233171326
0000000

1652233171326

242160155421526699
813502734485949673614

8022514517227172

635

2581514141731

64367137845361

346158175157262164212

5011526719026534634

515357399314488238340
1416479511083353

222117

10263734512011

33101522391413

21623

58748921443678

13216718418

54332313241620

11333151

6172562

71375648442736

2162025312627

67222035492727

36324852672244

0000000
21824274413205146700

21824274413205146700

152115202148334141141
16812512311

23202725572130

4940100581014947

27112419483822

1733121623623

203272593248

3427721081302418
0000000

3427721081302418

308150156209553135242
66204638943756

7467101

2913101054217

21312534361554

60121457622421

189111932322

5511141

563827261582836

46182219991234

693960631006656
1040516

1410143127712

3581617412621

19212615273217

10182617252814
0000000

10182617252814

211792625411
0000000

211792625411

5282460739883666686036313052
233923661312126516331216998

434114171632125

5816193431831

16165

52

4218

20232718321815

261

3216152116372623

1111412209

22

2211

74415335837743

112

1211

71578955

314125125123195104189

4438

1

20135253343630

35221518562620

11106261566813

42

45656

21

551

2723

591561110

3520161045921

8211182921

27932723251120

29181635211923

83122812520

167616944877060

972

41533115

2

135111

71371812373624

43331025711422

19202219813445

181613314210

1161311301214

412534

1

33

34114520401944

7311985

2320318321530

5937251831723

35235161583026

211

12767867716913099

10611113915819412992

702111115281820

31152021371825

22162020202313

148931029717710375

20291519423418

291044644

681616177743424

2

12

312989561136648

33362919421526

412

201091821312

1

21124

10

3615139331117

1816185422113

8991414119

67622

52145719625129

893815282449

2151

441411198

13

380253177124211148178

7381257

8311920157

80444356784558

36473

28372721443844

21

1193211

42

26505863396239

21

132

1512913182523

2513924491826

22293836301923

2815756633937040165

52

3111

3747540142121

1222

32313

7221225

15234137471514

1312319372124

44255

242142

18453416462426

21

845120141

55133639

3221241024511

23

227

58182532352738

664080425734232
1812159295910

7913912911

565412612

36134720233119

9436234522079152116
0000000

9436234522079152116

9313682139481411770
181322000

212432478307317

4344276019662239

2860222917982214

794943331003162
0000100

52312921741129

27181412252033

41161841663739
10267631041718272

1261782

33101374

46424536843827

51101131492730
0000000

51101131492730

2087203
1003663861206173

31171428291125

1783127352515

50111024542530

2040002
695910855653560

0000000
31125428342932

31125428342932

0000000
3647502731626

3647502731626

68225124912595958120
0000000

0000000
68225124912595958120

68225124912595958120

26444724205499081393311
25865142680327436026512132213128313668

929419151519204077857143415690
4611162319596

0000000
178712231625

178712231625

4921014
2899259566636143

1718154201425

491215816434

219532743301780

2649520315
463182276162237218336

1541411142812

1411820192014

22347811108

2910114923

78502232351971

98322424363246

1875020205815

7022129191335

29132714241833

21111922225

2321445413

202399231126

847916221161201547512115915090
310825837219872981735583

63108226

43954897813449300

4

380763531642963463682636

511

33

51424278

2231612231116

22

19102014312131

1111524

3591313141917

3229918241372

39631441459919155

15350666296619951

5144143493

17371519637

30295127

32191316623222

79231711272145

25115719317

65352153493863

334

5020162237927

391628719522

37391882034381751084876

5023169141025

92284081314050

2193611

5001721057
16794264532061531101164431129

0000000
12799476260138123212

12799476260138123212

0000000
62281928262357

62281928262357

7114191013468
0000000

7114191013468

187219141212138
0000000

187219141212138

256135111248
34313042286616308

13723912434156

181101201112104

7346889132627165016
0000000

7346889132627165016

1468145591179681077119
7456616290678644156025273

5911591211972

41988151575766

3486323950

11623815592550

1868394901023197476

11820103581706

367615313721162

1573411951354

1792692015

283317817154322692785103

2989848939562925753985792120143
1102174338258292954404

6943428227438157091096
104226042792772

721513141216

527215820816617630

7617047238041443275

46

4517611710014262897

213232318138
161668962635790

21231529

262543

4111416

611621

783978

7122011484

2341325

110341416131846

15635149798093108
3251100

1984612162212

39113830273929

3473353614

921532817

5951998

47532312928

42368147358231
1010003

1555727164915

26312320193313

132564432374479
40000023

87392113213419

201215911625

2158105412

1020000
51163046564357

22616191927

195215222315

951012152015

1521523513
000000

1521523513

7514142610
0000000

7514142610

355448414282091461061924
2521474611583

6632819

1031191197106375264

81343181488

218427011117776401760

172441051399580171
112131820922

411

61305182182151

214511152261

1542410141210

253351659

351913111117

4175130231838
0000212

699639

891374

277332012723

0000000
75831047

75831047

6992144236102201372668
26173030133023

532736359201953

7354172510

84384012211915

1552717118632028

1882952974335149

8617162761151321

63186114736

12711510410

15810104411

10234637195812

29112325571826
0000000

19102114321517

1012112539

16253166323484465130290674988
174722715335618912338415

104111012238

8358523

226223311410247

3837144224

123768814

122167101047

206826553622682121954193

2021251114514

39239181391336

4622

111356020259106

170271001504342587

40257106677819347

891136156

2393023127110829

21658

13135

1324

4256197431854223145

3654221208445429

201217357716

6331478

2240

721723616221527

16495101113

2649710187

1910815414

25881219419

43319911774

16553131314

351125698175671196

36321

5857486420119233516611437

132123922

15214863121

219397203

1551391115

102454563607285
3000000

197912978

14552172043

66333149344534

1556219215
0000000

1556219215

71476115392953
4400223

13151336813

191146979

2917374201116

6722112

1000040
41222821343638

252193171315

911131156

699561417

0000000
24233687704340

24233687704340

137961012
000000

137961012

88571123341065
129736666653950080325055

212461653251510294

212344142213468

10152014183348

769999119

15924186188124923

268111111117209785094

118312115314132397

1241415717423

649191024199

396973978359134425

0000000
82391257

82391257

247136629
23310411982113147216

2331558320

241431191617

1791420121015

18172312172713

3815114171029

112139247

21831392714

14126552435

19675939

241123591728

0000000
1641510152019

1641510152019

57011769120615460561
322756416366054762772576

1411

104319711106351

7083929194135

12930501221075546

10313

612

8432997572

431

31

13151929

1240201100615420877

840105901295786330

686182071793

892936111502096

40003010
1074610487128137214

10845171516

701345323933115

931615381327

777641830

7153229275816

592454324334388
6722046183724183427034

41506111314

19122220251231

451779812

4810912

7

3757215912617090545

1491913242317

112375

168491495440345999

17241371039212121

2613111961019
9573032635792522121079

29135013201443

82121111621

912916284171166

7240331811817

161601459154650

68113637353597

4671007911111767683

2511141815512

1058951071

181527284961608425043565193921112421
44365484911422497

3515639619
0000000

3515639619

175256269341506224241553063488105410
512917956439670001706266126810

22116141016

30912611711220284245

3538813431261135

927193771704552106

287994208795141513

56581392141131597

201020146139

9171882221829031275

721381323943

100618278234412599

354913245115480

681247960

3746654861665114

1249198981897614119

13081767033120021137

18758163610733

118222817019788520124

42311434115183878

6185128802372214

4711231119419

745114151322251252

7082624401335

5110181110219

2381620191026

1815147147113778102138

9372984856255766

14094227513561671127058449607

210353199991127747291214029

1492519182215235

561012021696125343878751400

7979133658725883

26443141310

361592191524

356471257447315

189021613587323411152

28686949

247573245153032239

1241432271018167

82685161513347309

84422938451080

1411114721

252468527813019317

369581011

39471221

16442

143491557445914630522067

8819611420357

1444712111

5207731405926862

734713454642395245486702

114023219714699226122

291381036684297

18556101027

104392094180414938531861045

3408324102120156

62710143136

120114917217883120118

236168841825

1571138

1111112

83712327248196893

1841010171554

34324621220

58172020392473

1773142183335544

58766953444911031582801

149720324916179715111

13203281102685741114

1072182143242

21038304560820

66938122928

141242343284476

146912142799

14

410

3

162613111813

154832011420638110185

676562011307275

3121

24411621434

107337135311023

199928897

10631183846299229213

32769121021

40375501385531260341175

2353830282215

1438112019381

3211610151613

12340476303105

7593803210121028149179

5793148296875010904056495
14023782032132621211709

45482336517139456

17104413723

2423141333826

1231

171

563513771243

1311

112

117175345215

1615406169259159662268

1031853932962961

11411623137

1580285141119251621311

16110318422

14

4138

2263612175623192

1631012206311148

211318465721
1849442591952347849629119830974

0000000
1015415031532446217628554

1015415031532446217628554

5420119459918961853433700
2311231501

8011201471556483359

4596107345117181190430340

32171017471919
273923899117972139

80241420691425

2083106318

32161098719

44113331014628

51101621261618

146349712

941021421100937
1071955168208666158381

1007513562021829

1323837567849149

1566291935794658

523636522452466

114247217

514317121381023

4112172

593134791642831
129344946274317033931035

1087280170365122193920

789415014915716335

694410815016110949

17447153231440303098
0000000

17447153231440303098

88627571122946
0000000

88627571122946

0000000
308514611310212274

308514611310212274
0000000

308514611310212274
0000000

308514611310212274
0000000

308514611310212274

44322230132236722933220282310070834
14325063044344003804261

0000000
41241123211255

0000000
41241123211255

41241123211255
0000000

41241123211255

0000000
2491220121016

2491220121016
0000000

2491220121016
0000000

2491220121016

3000101
56461894096911891206792

2714920325319040553
0000000

2714920325319040553
0000000

2714920325319040553

534469737716998801738
8163611

0000000
2013419213719

2013419213719

26710097134487122439
702221421013193

2911813671371

4318314242461

372415719937

211

8625226827545176

4723747236635148484
0000000

4723747236635148484

9153624183114
0000000

9153624183114

0000000
9101110111712

9101110111712

64514076344162
12976511387565121

410212325

11924

2561913914

62356

1012686

1664104214

3238910

0000000
1931215151822

1931215151822

2626187142626
0000000

2626187142626

2000200
25010413982127131163

3082413151832
0000000

3082413151832

0000000
314819272519

0000000
314819272519

314819272519

55231819324141
0011010

22633171717
0000000

22633171717

33171415152324
0000000

33171415152324

132698931514771
13912881210

0000000
18291545

18291545

79535820322351
2052541

3616321114931

4137217131019

0000000
225102685

225102685

21799167241562116456144131654831691
151166136211157175404

12003221118989701742
7466024129

81525175737053501

31165301617131112

9002115
60345841608056

0000000
30162316333426

30162316333426

107985276
0000000

107985276

0000000
11112615211819

11112615211819

661119103323
0000000

661119103323
0000000

661119103323

0000000
21131419222737

0000000
21131419222737

21131419222737

1913922183617
0000000

0000000
1913922183617

1913922183617

0000004
320138351358298319747

302723581927
2167313212410299592

18112818302139

231191181749

51109531610255

2144229252445

7320118158177

0000000
852118222728

852118222728

103823213
9660198216174193123

16661629721

32172439121636

13204437262934

251411612210413919

0000000
6748253320986

6748253320986

5049629474101119
0000000

5049629474101119
516102218

19172527333844

26162765396267

2582540
3044124717613146

0000000
15165613305712

15165613305712

13236056417034
0000000

13236056417034

0000011
173151195246217259212

0000000
2692538112224

2692538112224

0000000
35243956263027

35243956263027

892925445127
0000000

892925445127

1003023
54603463585748

176149141111

19331033183024

17211018261410

13292828364245
0000000

13292828364245

0000000
25132523222220

25132523222220

1271513203420
0000000

1271513203420

851841728152456
13334925740104794458936629213

0000000
1456471025393533

1456471025393533

105405433251952
0000000

105405433251952

0000000
608261432309607410593

608261432309607410593

15211477454376452455953
11883818130172898354127678031

33114453

6562169292752

712244

27151712241529

2083415102722

121905023553249

37441419391632

95984718421839

433161

1016727524331102

141733628412182

76702025252449

76132321820421128792

39161778215115927

363223

106952921412463

86554212323657

1211715151443880

682255108126212160319

3929

13818545345464129

41272537

44522517101434

45281915193040

267265111117137128197

5231513141141

1075392417363161

5854163544853

35563115452317

1635691078

34234610122055

44133

59762712281369

105303729352345

1211878346793795

51271628272053

104893319383580

38702622151642

81361225192123

7426119311433

113741427372053

140883248425882

681092626433665

911082568462578

124104502422473384

52699574443673842053648

392131232527124

526428116271953

143733054493757

5010231251640

208963024344892

78342425221250

11

121471630282370

57521219362945

90617257585540

731054622523960

43341624312829

1272043130542686

1434

225174127126194128119

8111523119716222176
3826111091732

111

418922018615320435

18

1004000
155122681968259117

553014161301643

43451711151540

56473720372834

21011073455335157
4050304

74351826161156

81392313111060

5136276231437

0000000
60921326182143

60921326182143

0000000
133965837477155

133965837477155

352741351962112
0000000

352741351962112

0000000
3229169241231

0000000
3229169241231

3229169241231

0001103
81174243287208354203

0000000
46130179216145285108

46130179216145285108

35446470626992
0000000

35446470626992

124533875810666998312
0000000

124533875810666998312
39249521421362452107

41203240251194398100

3252314303220

342975102628159

7271622183526

0000000
342349704912069

0000000
342349704912069

342349704912069

2000055
104174324315240345288

2930107565810945
0000000

2930107565810945

61116190213155188194
0020103

21711011318713132

21222743343682

19236039332177

0000000
12282746274344

12282746274344

1619331382140612951966343
0110080

0000000
10266110179859361426194

10266110179859361426194

0000000
59271364421359532149

59271364421359532149

0000000
51727802338113

0000000
51727802338113

51727802338113

874918137146761238156
0000000

874918137146761238156

154267577369470463352
3210094

0000000
16772021243167

16772021243167

2020201
6351154118176121106

1073012211510

1322131221931

24897731078230

14141221251534

86528915418721434
136929517219821847

1

2

446189413

41186153
5968107587284128

1793217152147

82561518235

1511269181827

15222511203816

371025381824182
0000000

371025381824182

55143805614363265095602616828
986748604445318

18242357272639
0000000

18242357272639

5923421659997147751
269551816191893

5423219121931

12133814132224

32144220184133

3427241061548

70215683400

12118917242129122

0000000
42892161817152923

42892161817152923

549914782764716131
3301011

4947946525829670

529131718960

43241417161438
0000000

43241417161438

0000000
7497124129129140204

7497124129129140204

2168102520541942184618529344
10233175625595204275196

584334262339153

2113298676

2716121322308

104215918653652691110

6651841923102851892072

72206209236214335190

431712282013116

631926283356143

331150634262267

5933183612713

232281274306274408446
0000000

232281274306274408446

47488715603638935155
0000000

47488715603638935155

84729917373100483
0000000

84729917373100483

9114127262241
0000000

9114127262241

49910232539
000000

49910232539

0000000
868012187128140223

868012187128140223

0000000
3824436033328146775

3824436033328146775

0027004
10873611288560120

38161824282528

33432753371755

37141444201833

4724155949045259693
0000000

4724155949045259693

0000000
995911212589146195

995911212589146195

2037611010984152268
201020111

12351507057108138

602460372743119

114106230
8132722649272261

3023227221937

1961714231637

17201648131966

1433165281691

0000000
21026242313578

21026242313578

0000000
34268528882474522311

34268528882474522311

0000000
502029553340148

502029553340148

3973714331342
0000000

3973714331342

1000002
1286570997468102

1317512131018

55274338332927

59212249282955

0000000
196106129167123126204

196106129167123126204
0000000

196106129167123126204
51343133313952

4442252

913102213137

27584101119

92101154

14113187623

239162174

422141

2214162710448

13313313913

231271218

54616367

1364217

211633659

2010010
68135427485768

56103820364060
0000000

223266282939
0000000

223266282939

347121481121
0000000

347121481121

0000000
10315712168

10315712168
0000000

10315712168

652516411007120711198243281
3518122635

11481334818310424738
0000008

0000000
1084101231669212648

1084101231669212648

64322517121282
0000000

64322517121282

0000000
89361917182543

89361917182543
0000000

89361917182543

53121010
2759415238244265199644

106481559878142149
811731111

4419109102536

2992123182956

251910763498746

0000000
32231924263458

32231924263458

26163416312016023427
1632622418028

17632252142785120

342412028336152

3484920263112127

0000000
24222512191431

24222512191431
0000000

24222512191431

0000000
42212126302345

42212126302345
0000000

42212126302345

18411558688458157
2000000

1407043475546110
3000001

39221616171544

4710175111531

51381026271634

42451521291247
0000000

42451521291247

865217389546
22448815976555974751588

125514504
3541269818913254193

156404721471788

18681461548037101

2366827313339234
0000000

2366827313339234

260174129658575148
11262301826553

27512016163738

39243110201216

82374821232141

1553219281217152
0000000

1553219281217152

490120147183163150429
0000000

490120147183163150429

3121819854
495226967710692255

93612514151040

76461214131336

152483415292762

1435017154137113

1688364445743131
117174118

70442815221638

87323522312675

172266124
132863129386833214343353630039

0000000
63362825295753

63362825295753
352179132229

69161272014

2265491510

0000000
1107349607074185

0000000
2715137111836

2715137111836

0300006
835836535956149

44171626251256

39382027344487

0000000
388713014314118859

0300003
388713014314118859

741613151026

318011413012617830

130582931365930874097321629718
741103185129155912242

265721176315543265
0000000

265721176315543265

0000000
38087444580581331

38087444580581331

0000000
16493767326391

16493767326391

0000000
35112197189153201109

35112197189153201109

1741914183553
0000000

1741914183553

44112033
13091803271351803185174

72110710351111392988

19235381718171897

392341856649262256

0000000
993429505632165

993429505632165

65222417292854
0000000

65222417292854

0000013
256100152188177136763

32102418352086

36154255383294

61492428242752

1272662878056528

19351655812470884
17213755204097643857207

69391195475

17711515759

3520731312160

34151725148108

34257825783531359

411530531338222

719526271353

3931269039278493046

1

1171454293420697

61143335162478

142139533

33442116372577132

153142610023
19232155216215832223

51477144837885

12561243610811124115

0000000
194585614274097

194585614274097

2211618619
685822128022366988

126281511129639

1201145202313220

6614341222432

35128117313137678

0000000
1207309249292250346160

1207309249292250346160

28111639840
0000000

28111639840

361293124113
462148158991351391280

2075090295481732

3112810661

431161615854

1065720292334247

3918211421673

9241889486851004764
58001010

778155744954783537

14125203730221217

0000000
2733121819445

2733121819445

94442412
245155199161171184260

50405230535337

56182915141347

35301833251747

38102324291644

25264319153848

32273036334325

0000000
171496213020

171496213020

0000000
169262219940

169262219940

43567118783101061697
9235628

344376845256401584

456218292351

37222620194154

0000000
431623451930104

431623451930104

0000000
73122011281350

73122011281350

2771921161881
0000000

2771921161881

11922252583443422431290
0000000

11922252583443422431290

90142416720115
0000000

90142416720115

20885192135170177554
36091718849

633698586092178

5892318227129

514062427070198

0000000
41143616282079

41143616282079

60031012
9963878010185151

30253140302743

33173320322758

30212317383138

0000000
373138161524

373138161524

3037626425552
0000000

3037626425552

7001108
15162595810286150

301619141235

532278192138

2981410201228

32163730484141

7689127120118140149
0000004

0000000
1642015201719

1642015201719
0000000

1642015201719

0000000
608510710598123126

516998918599124
00815115

17123318213846

19183635432841

15392137162232

0000000
91691413242

91691413242

0000000
212711383789678

212711383789678
0000000

0000000
212711383789678

212711383789678

0400050
40234224373437

0000000
249151012928

249151012928
0000000

249151012928

0000000
1610271425209

0000000
1610271425209

1610271425209

0000000
13754446444425875581741

13754446444425875581741
1309410225

2030235
537201314235251259663

9232001
82419055434577

1436013121128

59362740313448

23292986498105240
1451486529

1471541828

1915610121531

6118514

181461151925

213634166

15717791026

107332721121375

187610142026

0000000
3141618152562

3141618152562

0000000
47252022162036

47252022162036

1563530222830
0000000

1563530222830

0000000
11111916282010

11111916282010

596141299125
0000000

596141299125

0000000
5816191818478

5816191818478

0000000
1527752561004394

622351919543
1527752561004394

231561014613

32213317221527

331446361710

244491

673166269147226254959
5000219

1300000
2729116567105137267

892720212856141

112473521575199

701411025203027

332157922
2544167513244167

1651621291126110

5623451714935

0000000
42764111942

42764111942

1002731257653474
0000000

1002731257653474

18547324992456625622533691191050
8971042425701117

1496548266029122
0000003

66262111301858

83392715301161

96177913098
18114724386436624052423636181410

0000000
64443631444071

0000000
64443631444071

64443631444071

18038224048410822582194397180736
2729440138162942234

0000000
1621165708616918757

1621165708616918757

771069003135288680612837800
0000000

771069003135288680612837800

70000010
61813425153425559

448911991712367

16343661713182

1171916166155011
0000000

1171916166155011

0000000
1757122433619231

1757122433619231

2872592118474222410
35262625211035185947942932

102634996323914873

1263421043562491952115086

1830531505011633002223758

4255732271820805

627547964138267852010991212
636182118388561411341

2246527484111281862821730

2563030745173271793030104

32396041107331163886

26083788045271613404

2451339811741510747

22011877345362191
0000022

3521104101338
0000000

3521104101338

1859767304347151
0000000

82593418273364

103383312161487

0000000
6536308302479

0000000
6536308302479

6536308302479

8042376283063
0000000

8042376283063
0000000

8042376283063

3000012
2408171596183172

92494446425460
0000000

92494446425460

1453227131928110
0000000

1453227131928110

418001061
328043712810643268401

2743355907437187134

496743832581206

212189323012
0000000

212189323012
0000000

212189323012
0000000

212189323012
0000000

212189323012

3921959844440766576
0000000

3921959844440766576
0000000

0000000
3921959844440766576

0000000
3921959844440766576

0000000
3921959844440766576

3921959844440766576

58132212402636
0000000

0000000
58132212402636

0000000
58132212402636

58132212402636
0000000

2051668
58132212402636

406155211811

1672613217

451232422386361493479
0000000

10105647
451232422386361493479

22293847398117168
0000000

22293847398117168
2021441

0000000
53232811202834

53232811202834

6919148243229
0000000

6919148243229

12461211514
0000000

12461211514

251413481339
63231939272361

911

219531181020

8141

0000000
2324152122529

2324152122529

314171651419
0000000

0000000
314171651419

314171651419
0000000

314171651419

0000000
3055828195545

3055828195545
0000100

1932919101926
0000200

8323126129

11672717

1010030
11229983619

51551184

5213471515

195120253264233303240
2024010

14563150
5069166196139223102

2929841006511875
12153448375734

1

1133379

49981349

413241124115

4326

31

364

1

65884155
20367790719027

8274270456514

11331

134

432761877

0000130
1435185649479138

24103381315
93383837434358

371

1

22869121013

3693871119

118191615911

1261857810
50134727503380

33

36

22241220955

1321910201615

28403635343056
0000000

1395138116
0000000

0000000
1395138116

1395138116
0000000

1395138116
0000000

1395138116

15313122262940
0000000

0000000
15313122262940

0000000
15313122262940

2380202
15313122262940

5131914191025

8154851913

2176104714691221145014392336
42322923312573

8303695624935245111099
5000001

1415021
108567889837989

3027050
35192551393817

32312911

14951922611

1612254

14214116112

17210040
40153219141729

969465

1121124614

1910310110

0050201
32182014302242

174911171827

15146311414

53345616
7173134844044414321009

13366827410092443
3412146

32251159

2112524

86136106

671255912

20787181084

493251

7116131245

141412449

278101481717

565511113

1299771311

634358

12353569

42228410

435200267226250243411
45753418

1046841211

1978310713

629851020

52257414

52119105

3442927

12251614

1561517

12416620

6221171157

64211929181829

2252768

268216101118

16888111410

51328395

52353824382153

547115

7104108148

726431219

41041291

53293926371536

871410719

81364211

188481414

144186713

166294913

131147

125614111311

0032055
144441321008691139

151711357

1156518108

69108411

142314131211

15136659

217126

1610434210

643124

12118327

47226333304261

34333352425237
0000000

34333352425237
0000000

989510166
0000000

989510166
0000000

989510166

0000000
25252447323631

0000000
25252447323631

25252447323631

1117152022713
12706138456538538511127

594270373222364377445
3092013

0000000
35202916252530

1410234697

2110612191623

0000000
92507837696793

000000
2452111

2452111

178721713
90467335586693

21625

311355

134101283

468945

721126511

11622510

33191812181728

13227111313

464200257167270284319
0000000

76252422262452
464200257167270284319

4612341213

325141315810

23449

11756139

1515251012

511248617

4153121911

21775753

27866765

86347119

6266151011

14489125

1514226510

13942746

6212592

75637114

12615511159

105945110

1510429810

161171111411

123422139

914336

532619

16961184

514265

96108339

19136861810

1

64610626

2219111012610

620310145

17124217817

182962610

2151281231279796268
0000000

150010017
2151281231279796268

22183761032
46302136293384

15334

721333

15245216

13

313125

21

422772

211

1

249345

167

51247

34151321141325
0000000

34151321141325

0000000
2111151110330

2111151110330

59534251263254
4152534101311

2418384413

3120149121530

13917461226
0000000

13917461226

0000000
271015312332

271015312332

0000000
450198334284370371401

0000000
441195319279369360389

0000000
12151519273835

12151519273835

35265837534031
429180304260342322354

1

10411614

35182330222925

9844728

122596

823414

744716

82416810

82122

8110238

161411411107

1559551224

31881091015

121010433

7224210

97714497

1011281112128

322182

1047116316

531143611

7123534

133211

726265

10212416168

4511310164

17142996

106128151311

243699611

5111167

321614

2372476

7516591210

124101337

175510695

4336645

114156

7271075

238620311930

4244934

5151974

2

9315511112

201197591462448523528
0000000

201197591462448523528
0187718

89145421278336406434
33191731918

162612161516
4546191132140211168

0000000
20193227457377

20193227457377

0000000
593831282110

593831282110

191295625110265
0078092

955031354118

1073823165245

0031105
13114329312434

31313151117
000000

31313151117

1382715151312
0000000

1382715151312

198215673114150207
0000000

1293817242635
198215673114150207

7305921324180

11235935588392

93122720127
0000000

0000000
93122720127

93122720127

1125116217710511686
0000000

1125116217710511686
0026490

911519191210
96491451679810181

1767167132

83201877

361113585

12223

33812193

223443

25414756

353

54111266

68196312116

3255415

633445

816524

112447

1632388411

3322443

162154365
0000000

45163

12210422

916252220
44158092161171408412979181157697

121857418

43658064160671404312933180857634
116197399365335474302

11868124111156138117
0000000

11868124111156138117
0000000

0000000
1056268651189590

1056268651189590

0000000
1365646384327

1365646384327

0082565
295379674471475666597

139182322185191278211
0020106

63721101069410478
73107662

19182839142923

10283743394718

27233517352235

51299227287479
1122345417

17251131316

11829362615

1217359143131

903016182216
2581118526810048

284566

192197136

3153424

20245

16121

11443615344815

152

11222412233431
0000000

0000000
611135112514

611135112514

0000000
51111712917

51111712917

5660888885124121
0000002

0000000
10161011173430

10161011173430

0000000
991525172121

991525172121

813017103031
0000000

813017103031

0030010
29343335413937

17161514181514

12181521232323

0002003
364774564972138

0000000
19255021203871

19255021203871

0000000
17222433293464

17222433293464

0000000
536815812812215291

21284853487238
536815812812215291

27388958516546

132975

3265861

2210621

93111114820
0000000

0000000
93111114820

0000000
93111114820

93111114820

0000000
255089113868748

255089113868748
0000000

0000000
255089113868748

255089113868748

82249592583568606229
0000000

438520020615319099
0000000

438520020615319099
14205970353738

25145105

1

347102818910412

94745915

2

1

1

2

14101426172226

1

413881

0000000
39164392377415416130

2514529727631331156
39164392377415416130

1108151312

13198593879262

1087434477216779625092362348
5404492519354

674352062595429503175201660
0000000

0000000
21136334526214

21136334526214

1509241774152914162006385
653350761965395497974581646

5139271159361596799

8243624413430

110163777

13448548346627

891640181510

91476201113

6281613371

1813442092811

5201922252213

126715316412015023

291013171

841213154

1111117618716320638

9432338253628

55710121312

6114530215317

231024131

14108836

2622646237335856676

134146625410055

2476362476122

851910123713

2020941835637352032

145079787612157

13172440364623

1181926193414

16173010165

1163071494

6241261352542282191

8204623273816

1224622172816

2110311

219221981

1124853

551987514

309315314513021885

8181033293320

3271513141710

63238171712

7105237276118

1016221117313723152

233104143

21013713

3373029228147

1112326021822232538

420241181718

11015991312

34938511910512977

563522181813

5174021222022

21342123257

522242022

8151318292723

8337768497518

0000000
3707511328118110671485581

8260310969768531268314
2139583

1714533920520127294

1711515820615124070

45320575523458724105

1222133382442

3323814102116
286134222181188196253

15921011

84588719

4323137

42434314

810124699

638322167

3226768

205108142138120131162

0000000
2141024262114

2141024262114

3833907710113853
0000000

23141920494617
3833907710113853

22

13

8175840406227

6131712286

13107615
53527913878829221278462

0010020
3941301021615670919239

0000000
33391922538598791132

12198463535621

32172838475545735111

0000000
563758604810079

563758604810079

0000000
524017242628

524017242628

109101244208192282118
0000000

8224034519029
0000000

8224034519029

46116359587147
1017920417414119289

1481266

6453152

2525236

46551261067810728

0000000
15134215222676

15134215222676
1352912192362

81321313

2121

0000000
16327037325024

16327037325024

0002130
3364348498087111054525

0000000
14447514611756142

743627252523
0000000

743627252523

0000000
1374315199231119

1374315199231119

1020111
192387798760593995383

0000000
45288054376338

45288054376338

0000000
1892821221836

1892821221836

119311647640490849255
0000000

78217426391349615113

27365776617939

51395103516346

9456970299257

9394145436453
0000000

9394145436453

0000000
381616251529

381616251529
0000000

0000000
381616251529

381616251529

0000000
459215620114119169

0000000
459215620114119169

247110715410617147
207110714110117143

431

1044

21214947352022
0000000

21214947352022

12393115115130158117
1000000

0000000
84312717442049

0000000
84312717442049

84312717442049

386288988613868
0000000

0000000
386288988613868

26183024184342

12445874689526

88370552484433737258
0003060

0000000
1394123262623

1394123262623
0000000

1394123262623

0000000
75361511458407705235

75361511458407705235
18881327810214332

193347755211345

2410075928212288

1414025721317132770

16841766214081167
0000000

0000000
4264124283156

4264124283156
0000000

4264124283156

0000000
12635353811250111

0000000
12635353811250111

12635353811250111

0000000
7970118112115148128

7970118112115148128
0000000

7970118112115148128
6325142145

18253844335032

721412141324

13131320293922

26162113261622

91179111623

6139231238140310109
1164129430052776231030561833

28487652557491
0000000

28487652557491
0000000

28487652557491

39334048695845
0000000

39334048695845
0000000

39334048695845

5072104
124116216256159167166

1992255351516
0000000

1992255351516

1118713102033
0000000

1118713102033

24131824152433
718104414

1211597913

51554116

39156332434433
2331004

208391224913

1742119193516

314132568
266199130556447

212252218233

115539699

5296034232621

518336

0000000
49317341544765

49317341544765
531534110

34202821372839

1083017131816

0000000
513556485178106

27263532245353
513556485178106

6

2492116212553

21297864667543
76194221811946167522301122

17234123264935
0000000

17234123264935

1620121121529
0000000

1620121121529

0000000
1394747336547

1394747336547

0000022
37466152455049

23334022283037

561411536

617199154

363

2441797282032
0000000

2441797282032

123591239090153158
6338111925165214561966887

22101611101723

4441610038546721074117

4010815182733

20124225322626

20313623371234

25142936294836

30171816323022

14117148817

22173615181232

19103643251438

30231730172525

52252231442760

26122833393332

68185101811

24143721183227

25154038133138

1743819152922

266926417522123654

10155129546120

5131366211727

33163363333635

0000000
515013214710792129

2000000
515013214710792129

1692535291638

12124525272016

12103255202035

9193032313640

23177035346760
0000000

23177035346760
0000000

23177035346760
0000000

23177035346760

0000000
69761131198885325

0000000
69761131198885325

69761131198885325
32483156452394

291356283250209

31214134411

5312227811

13101514181212

1612517191213

1769781658156513732245474
0000000

1729751644155013602229455
0000000

0000000
10278966547933

10278966547933
0000000

10278966547933

1629481555148413062150422
2054070

104614104210268651479284
0000000

104614104210268651479284

0000000
56334508454441664138

56334508454441664138

431415131619
0000000

431415131619

000000
3613225255

000000
3613225255

000000
3613225255

000000
3613225255

000000
3613225255

3613225255

0000000
6212318162128

6212318162128
0000000

6212318162128

0000000
53563210051173118613261514

0000000
53563210051173118613261514

53563210051173118613261514
31763215

000000
618172913

618172913
000000

618172913

66320463534428686243
2010400

0000000
14397651466543

14397651466543

0000000
37265321446361571156

37265321446361571156

0003045
13166537175044

46291252015

9103622122624

195132233254224334923
0110002

83210241845122
57117161180163282875

235775937413181

2628766371106672

0000000
1255932433721

1255932433721

0000000
12691242181525

12691242181525

0000000
245116218290461186262

0000000
210111191278422172226

210111191278422172226

3552712391436
1000700

18588201117

1619412319

0000000
16544953589044

16544953589044
0000000

16544953589044

0000000
491719101914

0000000
491719101914

491719101914

2465771017212943
0000000

0000000
2465771017212943

0000000
2465771017212943

0000000
2465771017212943

2465771017212943
0000000

2465771017212943

8063330238117121893162343929200845225133243855420
2192378655405928526073023111

15604004795880086998101843194
0000000

15604004795880086998101843194
1512148232218

0000000
1323649518326991028322

1323649518326991028322
0000000

1323649518326991028322

0000000
20561031269114835

20561031269114835
0000000

20561031269114835

1393357268907042618589862819
4056116149104143108

20162136312354
640119319862203215325541105

377720986113212341275646

24345797910358881256405

2227611334134010651739440
0000000

2227611334134010651739440

0000000
1947111726149313752202610

1947111726149313752202610

2978511728185714882348556
0000000

2978511728185714882348556

12101938222534
27119767164601562013543220985531

975988798537551294275
0000000

0000000
975988798537551294275

0000000
975988798537551294275

975988798537551294275

20697866133811286110996179214491
99283338426262495273

0000000
788279450544653397163411672

788279450544653397163411672
244173391535

72203290305244406192

29410741828172714832455543

30412492505217118773009686

94264414417358456216

2889421742178816002456704
0000000

2889421742178816002456704
0000000

2889421742178816002456704

0000000
650316650804728422269521359

11102431152633
650316650804728422269521359

2127001180113710711664530

42724563876356031365262796

244681116712669411677483
0000000

244681116712669411677483
0000000

244681116712669411677483

53312932181186817702858731
0000000

0000000
53312932181186817702858731

6286132220
53312932181186817702858731

1888411486119111421875400

339450687671615961311

41924453943380135205660851
5008405

986449487828831294261
0000000

986449487828831294261
0000000

986449487828831294261
0000000

986449487828831294261

17513872339239320233401346
0000000

17513872339239320233401346
0000000

0000000
17513872339239320233401346

17513872339239320233401346

141414656618610965239
0000000

141414656618610965239
0000000

141414656618610965239
0000000

141414656618610965239

518471192353123824541146331461936832
0000000

518471192353123824541146331461936832
0000000

518471192353123824541146331461936832
0000000

44807956304582
518471192353123824541146331461936832

513291177932924322767130741212335774

11052910248847271175211

176427484455449714463

142316321316281475253

46908763728749

719928600212451413300211956116017379490
7995613223269721087552270454194977824326683791658

0000000
121684362522471523228

121684362522471523228
0000000

0000000
121684362522471523228

121684362522471523228

9635337809780187068115222151
0000000

7571110919
9635337809780187068115222151

25413322220222520023045438
0000000

25413322220222520023045438

1288291250122311351711294
0000000

1288291250122311351711294

574317146204559392167571400
66131142188117210139

131737105311019511555335

16212221819152914502596437

145759119112229971738260

70322415519406658229

0000000
39545438787577746902116854296

39545438787577746902116854296
250688616096178276

5919644337636755266
870201228272707241543101030

3677329979087691554457

44410841387142312792204507

4114727162832
1315177227342603236741271355

520230325384260540397

75415282402219220913559926

1519158622282304202430701635
100143067393970

3694557677346601059459

541832114111189671531575

509285290385358441531

0000000
944738027696781003378

0000000
944738027696781003378

944738027696781003378
0000000

944738027696781003378

0000000
40721073282307528324653855

40721073282307528324653855
0000000

0003020
40721073282307528324653855

16810491399126412162089294

1053857727866561078212

134673111110229601484349

156777512512023811168610319816290332580
0000000

156777512512023811168610319816290332580
604222337593586330550751348

3059671509148114822109619
272311522180761730615833247835233

26510651442149312381996472

25110281579148314532306402

23913181818176715482625492

25013882744231821473143487

2219881658156314482438414

154471795768689954297

2069811504154113402038451

21410951748170213822425492

2298061218121611711770384

1635547527317371038312

2268611309124311981941411

18798026131271204211088175484104
7884391116312158087538628568716609

56177375411294401103

68377609502501735150

71354482422415633210

1405319278687591244245

29791321088616970

814797517706771099243

117776765312927

16610652035184717082630270

346897946211870

70369511491415672143

1036148659346731170263

45169195257199280161

71296486487397703148

1066029828088081350209

1687569789988871369319

72171092115

134287394397395612233

117699109110639711560272

974808667207041140181

101444848865730938225

16142443281230

51222244184208371126

104713112610239931513233

1347921446114811131804236

9102415182526

165275918412234

1025609657807751194288

1055649238618271465252

148753110412099971559367

11872110279188621444204

20613922113192019073301321

11571310979369151556268

1134109337437241247223

55368551498489762152

3242628193921

44208317330332517107

124102157170141297141

124634107410188581365254

5940272955651883999

4417521117221632277

358111280101174104

1477601507130812351926312

1729251773144114652338317

1337641258118611831654322

1185237036796431082218

1175757878158211154216

1076069688828031457203

5325139532130457595

5182223173622

875328148497701132169

84761103311149751329179

54222143158149237118

7453110198607791325154

1149211350121611562110283

57277414445374627136

1187741053111510501502235

563788586412279

1075296678027171017224

1175759486648741273184

955088167126311141236

644071015758753960133

16811121712155114002343336

965459538567561298238

28779512610214091

18211272197194016912562339

207772885511650

1705487647296871113385

62268433498452669111

41321293090270824864176939
30801107466104106

1475727557606961076342

23614772225187417242996491

1659441848165215442333313
38118813370296527204319761

118486646520448800303

673887817156601075110

316395786811135

0000000
1847751261106411001650328

1847751261106411001650328

559353653414896453170561166
26283024312641

31418483060264625523884568

21916602251222619483146557

0000000
2046921159117910921578407

2046921159117910921578407

38922693264302328884563840
18659557146361365312715202013876

53125145167191232115

12667210969329201319273

31914762452231819083477533

29914072072203018312910655

1777731142113411201586372

42324783923358434535531866

79357542465404583222

1316611180110510111522310
0000000

1316611180110510111522310

151297773795634894274
34811672007200317722709783

111

54258431409409587152

70342475411340682156

68245315369370506182

2

5251219184016

112436769707631925228
0000000

112436769707631925228

0000000
26914352469242721403222592

26914352469242721403222592

9233383185986177213533408182918919
128397570672548796304

4869971019690148
337016287266452493522318357077636

18608680140701338011796187524096
153581103010447851298383

27513661978192116902721492

1979891388141111701834473

23414332532232520383354549

1686139348628211286383

2009211588149913422112488

25112462127187517812788499

153468730684624954324

22910631763175915452405505

791519171729
40022083158311428074674916

23514842100211119542999522

15871510439848361658365

1889171586136713672251324
0000000

1889171586136713672251324

2659761670160014082034709
874441377346973625299402152

6382214102438

1617249749378021365384

29201413296

1579011567130811891861377

2614940235133967465

11143524274217

24616023044272524643911556

289814196214232033518921301316624
1155907778327181116312

110131172215
35911781658166115642513783

1869511475139513532175464

172217170255204316304

17199086139771309212404195953987
115403656690604812314

31917872964276425333984700

28316112308220820443504575

125425516487458701371

164698104191110221513424

22513481925189218062785566

1768671282128912711976397

31219473285285126664320640

2303134
46720953161303226744460989

24914412118200818123104524

216651104310218611353461

0000000
23812471850171815612447553

23812471850171815612447553

28377438112233127111553151954355
107204250516382271140

10921136016514387498194
0000000

10921136016514387498194

2001000
33113142147225424602847693

13361610339398561244362

1966981114131416041603331

1657326045608242488062912280
5238701408206916291927909

6262771109411398

1404957466866221066239

218490635735688872430

3658651077336811721573281

292307374988408443199

57171243286267297124

633244939063745344452881048
57367567625484727122

1507911255115110491736314

1649681660144013292208360

262323424529582617252

49411821720165814432350722
0000000

0000000
49411821720165814432350722

103916102117
49411821720165814432350722

19859811069348941469403

286581605708539860302

0000000
21677837872152233134581505

1313611725
21677837872152233134581505

17771336982083226833561385
1570298176176266116

136014311193112116

2860426243217298172

4747500253357520197

17266772475341

254062057109212771930665

324420713610117778

03111381512
38627663529595

21452620103340

831811232917

9112119111826

136056102131044812713110864636541
0000000

136056102131044812713110864636541
5322199522784

0000000
147523313311030568

147523313311030568

0000000
2065717840

2065717840

183475111391419277385194
8432220656395569483679799

2319352137316147139302

2714777258120747114894

119026449139348104141

37562556916368

103292321342689
0000000

103292321342689

1407755181625381466138612215
4850378773854230212444789634961

5412834181626

933913133

262422063192757

8202861424121

18222233112337

8193667424426

6143932171714

437110320411815380

3771011622111299710926

18273524113349

31377288536840

1101391003295953701711190

311853109615171

73142612939

12435685385767

561934292253

14371116

4724840940434560092

173345617168731669

3742011814

93305512912379104

51687651048569433

2960751858499100

72184512471324457202

41

12434672113489111003340

41467113150947702967

284674109110158601277247

834094320151237156

311516133110

8313398418476609493575

631127182038

2928448742434963166

1177181147

10193069404020

0000000
221499207713215312695511871617675638163

221499207713215312695511871617675638163
842314132823765337839871380

15601125291627
837395159905626534884371736

33617292538261524063766735

24810871702148914442360522

23810751739149714692295452

0000000
26013212047191018692816506

26013212047191018692816506

0000000
65173686411867911455799

65173686411867911455799

5810249727872102
529178224192398217232511195

17067010159459141386380

116422555563445610380

1855888008187351183333

1941110763973966842295
0000000

1941110763973966842295

0000000
123263328374297486323

123263328374297486323

0000000
33115812320213319532886559

33115812320213319532886559

857189824562596246329881440
45588383978471

277371517665705657275

52157163160146208175

1056268937297801074218

139304402447350459255

105230273345259388226

134152125167126118220

335411759100641141611196132213606
39420371205163316031349548

5720016118919827195

34716372038213320982190526

8467310088789681507222

555323630923268309439441021

34417991260154015491939616

139625584734651878174

14341552716104110351143404

995737717956871164289
29112331479172813752276741

2123315244012

7317918

104430500624506832270

79215158285158239162

1352688310387100019236143053079
6968385116259057535539728420114735

21710951957176818472862334

82332562519450746134

1285557897798301071270

23410561705157814822262468

896441323113810271520187

25465968576938

151378772752678929338

1278051291115410301712305

1549041691138814152185243

21713312058201919512964394

29421673134290025824790686

21410111410144113561945497

22111931786163315172260353

1619171614132713532040358

18511112036175816722701369

1519111265117010871805310

1568811762153114142195437

9952010969188321352190

238015113511012854

72146252196182248123

24016722662245122693638566

15475698659641

20410552339207018693061409

31021163423309328434733601

1768531627158513952415383

795956787096741060149

18710601526136912802044478

1667781386130211211736360

21110311427145312552018404

23425344336038

16412992383191618782928409

24816282928257725763628410

1648871206112612191627406

1128901389131912611804221

22112721902181618142659501

78258433382245519147

18627252678645

34918162842263125193890731
106532731661674971232

1347371370115111501820274

1095477418196951099225

468613505188111843117125252226222
1024308639343945373352701287

63924673984358834055431860

238134

555644688618708942632

642760611639789660437

2738601215116910601723355

2409341290130512242076404

31311541476140612701993753

40417022719273424863432533

11710872017

27510691740178014982245513

308819113612369391426429

232

29616383006246523403561585
0000000

29616383006246523403561585

0000000
2577621258109910081665437

2577621258109910081665437

8874667769768236501102271943
19734036392962

1055289778308021380191

23213562271181618292851556

176809110710519401459406

35519013302309028914508728

0000000
31421053606330331444946725

31421053606330331444946725

0000000
123298331563299399205

123298331563299399205

0000000
676208830152862250136524265

0000000
676208830152862250136524265

676208830152862250136524265
17945633

4014319519316425976

120208376402333484254

15865710649828211397251

357107313711281117815063651

0000000
662361155455102473976951472

662361155455102473976951472
0000000

662361155455102473976951472
71359629635590977167

33420763316301928254537803

25711761600144813242181502

627918410067182963642856212778341788731544468
19684583876274904051180

0000000
15967336133261227111182171743725

15967336133261227111182171743725
1986549779108831305501

103415737662551808245

1779852069179116792592417

1848081591151013091981497

24313022476230220743026473

1668891417141614182187438

137249691583576760224

98650115810248271441247

1667241290124710131753479

1246609208268521321204

23787426606426268719585828501229841
516249305610410393660

3000231226724529328335812774
19863320804729351980439796299422451

37711161973184216252547574

145262234394310407204

1814789158607941008340

3015457958567441143352

7127471149145510561722629

211457409559442630307

186403604714690809209

2468941416132211971828390

339568115010119151409306

82103116182163164173

282265277332420359347

3224926797297051037397

147422675613579965235

3998631698167414792256370

4051123198444367289291

214544671610641996415

174183373436394382228

56154687310237681180646

17518731237194111671371836

24257798410148601161338

3139271353114312031769377

236216229307216345293

2886009209157971302275

2408151454149112211925407

111121178256273193156

589378428721548575503

414521813862722974411

3148431471159612571938371

171196252258197312288

375227343455352331274

321424433615454607349

204439617736562795328

251302427571545798310

3528391185119811001767424

8089791379163212751823576

181484670687601863337

3889201294137412111623528

1975609239297801180277

7309631936170215132444916

158482788657617924242

405551719746666994523

2008251494143612691983331

185165304253236273286

436910410283136142

188337758664597854293

2998181338122310681894419

3157211384126712821798457

26610451566169414372265392

2155749659937841168352

2858231032115311071715467

231476480604504794338

1685187949496841179254

145149164251219209194

0000000
4969401577145813092233637

4969401577145813092233637

0000000
61130045205447442216306986

61130045205447442216306986

0000000
2296681311116998415951415

2296681311116998415951415

0000000
1809511562158913872023482

1809511562158913872023482

89250311309271422249
0000000

89250311309271422249

194289439528387503265
1803451866987130602190462961

6186124170128167141

7975172200162223178

169316405441384562334

135296478583501692215

179466704820648996234

1638441500142512471960329

186819117411299841400335

259305280401289459282

149488601615587882253

104183181238209293124

125351640580495909271

19111381970170716002552494
0000000

19111381970170716002552494
0000000

19111381970170716002552494

865512943606084711753130
6251642955126216419202297205980737301509228

1503524684803656681189
0000000

1503524684803656681189

338426705923802955830
361024423821143

124162255386317365262

84107154143134171184

94147272352313398241

5722415573
483112216162252181123251269

2453975321204794816641

2337181082102410021504555

0000000
10677116121107144899

10677116121107144899

3316738112551
1825308247644789392064922345

9438091137132010161740781

1613608006776071145410

114114234264226267272

19274810139777771254347

38210351573151312832061484

0000000
198198243298243354304

198198243298243354304

246593311161155214614302275
5391118822161

1205287132113120358

1776538485430667692622

51633453357513585961134

3141174105041
15755845611213780886751112

1291511493114957775612700

253696746820863371

6231146942842200450172342181623527361479137
23747530637843667566695219948505

45988178901041138384140125637

784178199229225250443

5499043803569180592152746165372474231369865

1062611213273443661571441

437230894479855108848124230633246

371465959103110851101998
2899280139985155465053488926

163213282363275363425

112113157258259223393

15143943304063724574967

1745938427957081080440

113197350435357567266

13912883966730203405

218405462467462674771

95293533434402680261

388108231721216437
0000000

388108231721216437

143361921326117
1414130719992020173324071375

237304569686474747472

151210314307255353236

883757109710069721301550

130270434534408491329
0000000

0000000
130270434534408491329

33143948565666
0000000

33143948565666

0000000
97256395486352435263

97256395486352435263

0000000
55315588461415748138

0000000
55315588461415748138

55315588461415748138
0000000

55315588461415748138

220827231622378061493926395098461396269424
50206706113321407412347138588415

000000
833448

833448
000000

833448

0000000
79415752410336421113107841

79415752410336421113107841
16151252132115

3564005871596552814287

163365543511482682223

2597951268120510641590316

1000001
221573104210419631305453

0000000
55107171251150227134

55107171251150227134

0000000
1224498337587661050247

1224498337587661050247

43173832472871
0000000

43173832472871

31310161851183615752219665
0000000

87154169227175245137
31310161851183615752219665

434910610389173107

1815314217713626146

125397790740680950324

4026364458949559051

735258945373983381456081287
3241103

49515982692248723653568937
177818715015015740

6480111149116140173

80290585493477714208

1784779358517641077263

1566738748448581480253

0000000
2379891841149514482040347

2379891841149514482040347

94663450067839817310012093757028100796
1752131526663006366416092555

0000000
4053107124939597

4053107124939597

0000000
4392731482265

4392731482265

177497639603627835326
3838961130130511971536675

48199199241261377102

16274237373210

788914213716317193

881913162912

19282514182037

317633726

121315190111225

419688

814134

1181158

9152146244224

858387156142116112
50404366754565

10191654474011

25242836203136

7468123741914819331187722250710454
975103419551984218219171331

78105122131143150120

34268082847761

635499541219770

58153676

789112111116316081

5037448011312281

2960741329613063

126365662551522707189

16164458713469

513669597632563

933377152113136140

23364848523816

72359099824977

1395398217146701068205

4634568410611377

50557571795376

2011119919517624135

14213513191616

258267275424278414295

445825356828268

76112228211220231144

98321496432393639173

64185433423420544112

54535810011460103

7720038431229438190

11640112057848751138155

12861100126161126122

41338583919053

72666584658693

9024569333313160280

1213198747107861012199

7869868110214788

52213655835570

705313213610812993

18153014221916

866613011011013799

66101141112113133142

57498211810677110

27253569704954

3776971468126313841876357

11773028183418

58218302404390524125

98442813624585915208

34368691919281

510202414149

1034848227586951022175

1171301125111574584

205125202350229195243

325137137

25474149803744

26105819281916

4835858847540780174

59862985616619271144477

64335477442398640139

943230250226644198962

4464947710911880

851210232517

6674163198180207111

88104182171134164148

53618991717485

246400536519566716224

537078109120107122

132115209204103162138

63312552597505719106

50213226320282332118

1073346426706861046242

564356911269986

132162283342233313123

205470768831761949334
3507541

112386596647574737202

9079172177182208131

187844060621054613351455930936
28511715712260118888788870546353

2737852646592467

1051177791142317983184193

541265421501449230687

83945516451391746790551

1484850515042259

6522191028322734214123978644

275147237241173267483

781722221510133

2280305559864375406059271856
361263463341360352255

785233993786648

62159423405343438122

5210924519923628971

586259505387322593103

5111319918618924267

13123018131026

98344617401423632121

291511431208992132

104290648533479724143

47223326275241400118

90225444244260456109

19895731554175

41337137474436

24268510018687381360240

657515115310683104

655628961586964

15173523233622

1256991152775510966846332770519092
8954511123168264915561284

143201069462637107

2218172578870

25012217736114871121282

29039410449951843853250

5662548118320989807324154711039

182199288287377384135

371259363255853257300

10131757955135128

1976534280186922923863508

2677160634416041244103530989

158287513457399543293
79194048443395

78268473409352510198

13

0000000
2931591009243111

2931591009243111

2152938394238127984
0000000

2152938394238127984

6314021046970852674433
111655223

292126227147257102187

328275813818590570223

69110981687204121302029768
115260249269278272165

42352853855964

20757510269579861203221

374228563277192

290186356706454424226

421168132502398242793
2411138015104224578221553761

706811774058132

92342044119069135671183

3215212318745

146616217912082239

24880115344237130326

2315029226324938554

217284269365303478329

1645157177320447319

1203979328212103211

474431608856130

37089719734311390327444223112719
131322512933989958539

4387939439724364

1202136468631141

15730936561170142

147602351949783946963352052

40168210171295851711521749

417310742059894531106716

155842667846318383742466830

9060958712879104

5382217553082

1035518029614355134
0000000

1035518029614355134

2484357459767110551573084929
5004911216418539418581616178425446286852

0022300
2255046837586671017437

8898182237153217163

137406499519511800274

0000000
5551875529987425937

5551875529987425937

15649387012508451925408
8693951671574656060112381942

24715592614255422514111558

116418694739641936204

1316169739989331332254

10555398212557691624270

1143125826696211310248

1447981333132012451981350
0000000

1447981333132012451981350

6188081169185512741892696
236321571787560857299

68616317912615583

13489104273151191115

180337431616437689199

8184625941897641855687
215362089

5332983241294379481353

264159267541262366325

10002000
335910551573133914261914509

3134302329391472432260

21575312449469541482249

0000000
1649821269123610141894285

1649821269123610141894285

1904598829107261164400
0000000

1904598829107261164400

128449787733561928213
0000000

128449787733561928213

12314471415
44021833897369531594969835

1748571683154412622079300

1699861731164214532234351

85337469505437642169

963736466906261091508
0000000

963736466906261091508

4976191177147225190
0000000

4976191177147225190

225137287322276278195
22015520916189308093122922603

28210771372136913312068387

2585489739019161204279

20962710189498131316288

2038441655155813362271346

5038361175129510681478371

320640113498510001573393

2018111547155113532104344

29014272182241018903077588
10394293733475716212101541914

1154549117096921219166

61173297587272335154

104361634564517868208

6316725626922840290

1898141244137710951911364

3810116319018719564

85484107810088851451166

94312569457446696114

16566912189848951479295
0000000

16566912189848951479295

90134365279300368227
15185526376845

3659217171178222106

1432322232423

9381727231722

16165333393731

1282622373032
4297271255135411801753968

208265449520457607306

80130190169146235217

129324590643540881413

61113956662677989265
121420618219829371

213740622723132494

918120747014228

144115213412815459

537245507613

170241417422397546252
827162325292487214229951323

50166280217148293137

124109159147174219195

7789151165157196111

43831191111189182

9014014714515120286

114381684707521722189

75199269309257303151

84215303264219423120

10065013779538011328269
0000000

10065013779538011328269

0000000
72105134163129208168

72105134163129208168

1736539129848421575725
0000000

1736539129848421575725

0000000
1091624222320

1091624222320

33155599
46135484348326453149

1585630325553

2412337428326634559

413930234428

0000000
2234240265129

2234240265129

1046296452565102448373531674
17456510359728141492270

34114142395139

28557676537968

9958697810068881320314

7878210212189357152

1365971223108010041559305

3105869359218151340268

1874867587936811155258

19047126122731191510927165283893
1253558957827111039282

2029881771168516422483338

1516941082121510411576287

1599681544148113392079415

94436707668679971179

1356811365124412461768309

108208250286262358176

99368625647562882185

112447669689599942233

2008741952170214732512350

92122253231254292172

2016547498037181071448

113156180218180210279

113175231264221345240

742010745175811768015675292075909
15434177704571056487124842270

174341551431395678209

10272115294311

80351498564485873143

7231731143722

46264224

2

112282486612386658175

3721356042841963651

915915112111

267111710910919938

691618163814

131142281218

17403331276829

473745895510385

134412730789682968219

585512215311415094

833433

6132412132512

1172326322128

4018728736329946177

7611514016316419298

1144817587377251128168

21213245505924

81364654642607947138

942032202913

8276931268513185

435010610511215381

64277466420397681149

3019333728432542870

175210378466288435155

84412602620533836125

126510645680562975261

201945564613132

12115141321

117425486503402718180

775671358106510001681118

137262253265237414224

79187356356255485159

37352972542582622291275

118354591690544821320
1104211

77219408474358556215

40134183212184264104

3937671528141612561718915
63122311423

62371009273104103

3555234193136206113

74121231215148255152

80178192264239272224

92343663567582811199

443096626766101

34412652358234919983062555
0010012

1644689579118021056168

1034598668606871196196

77338534578509809189

2019348161596012524680292720
64411411780175114582337926

148166276281245280156

500607103310809121387379

364667925712387

98401107610059511430138

152129235212184299159

29199317326241432116

37264363534272

66396836515582

55789011685164149

6623546935638161977

71227443408398558130

88137221219184250104

295041674653145

12835236929786628142121602819
75102235263207194163

1727291511123112531873300

61235347410377488144

84376604634579739266

835708187887321202274

20711371933164315752596300

1467651261111810481763327

2544125998510096

2543761057711298

43826912112597131

8779134136145101158

6514443631933447993

131608108811499871495289

79322660646618921180

97383653706602922222
0000000

97383653706602922222

22712842333211419813155385
0000000

22712842333211419813155385

198244806475812749076613710132140567
48039420148491532612543194199214

2114716716415429375

54442260413331

158204300327280340252

293332495589492524270

144379714662605858413

4196871277136396318821233

131272380440326568216

221416593756577844604

2441149129180164168159

139184309474269431199

142198321404309505268

1396921207102610841579322

267410701751559905515

142112104228135205196

215210214010113036

1456059399397871213324

77421671665575791109

140256370362327494275

47011031983166016832838679

48303369497165

5623738237433856773

20141224297145

114148291332277432239

61365934766721960184

9313217112220

2211410712513215292

135353550602480774251

20202628357233

2117481323127811511741358

3014608417076771089439

80116216202163249233

172470609668548975347

191356426415477677248

75101887869115113

7201416181533

401556515563103

30512811910190317752998801

2089681610154714002078368

12594170765881191

142322407543426706315

221739130310389311492449

96201228267207261128

17453710109189211397286

43910391561150712842153510

2074626576676951021372

335857534768143

2658481453130612091998506

127222273364298408224

148301537529407710263

928147719652134164825314390

1487061168103110881582292

449559691884682900607

266388635740587896448

167144226248204282269

111119154182143194223

2196571784160914942249362

131240273284241378250

1552672923392523111160

2288671538142013861940443

172262406473404600338

6542941338510994

18978811209799961477343

158750104710279421537231

2147031220104810931605427

26654996696615431593391

1565528958348331199258

102255325401239479536

137399634565439721355

128124183212172275228

321842

202337392437412566379

1707221554124610321932421

2211342

120454703565522914258

8364261428729

26361742253733

159739100910329431468324

88238396357310564165

4167221139124911441611710

1355007587747731191216

33021181012

1918371414125611311732352

1627381410119911741777342

2368131271132611281693509

376123425

2058591465143812461855398

71013815

2538661784145012981911506

243494619748662912263

79181416409372483159

1867481323109610271662358

1406188008126581274284

33101018103036

1464639148718701298302

0000000
411439415637113

411439415637113
0000000

411439415637113

4933609187371103091601211818373
85143916210123163

0000000
31710591737166515012267562

31710591737166515012267562

306665100510099431456397
0000000

306665100510099431456397

0000000
157323414477408551263

157323414477408551263

136212318459299466242
0000000

136212318459299466242

214214264404342414274
0000000

214214264404342414274

11369558
448467616946629813491

167130261365211249162

270334349572413559321

8536877571354109611836118
32703137434459084937612815981

375376484631559642671

182524898100981338

4561401772172143294655

24345687294083711141360

347735100911759351470464

31384154213229113318

501607843128096711791057

5954522311762679
2900504585741370710249102193630

8732643422147270
1879281053389598674260182253

124445870775697

1714459169828681302205

164171243556391290229

175571106213948561324278

225324616872638737313

246182287509394324310

5227091703379827661418515

1653323891075538495236

962218131843878333141751298
6935701741106473

4429401517173314681841573

161407487595593753241

150476635741668953206

140323475635492564205

120867714481832130214142113
0000000

0000000
120867714481832130214142113

120867714481832130214142113

0000000
33088102851014015770129971392316897

33088102851014015770129971392316897
10422278825455485338334445470

2109587138124132127

168434673669097

17856371046791127

548287030915087544813683

25689108149125124152

133249577444433680119

223203253351421508851

49710510630499226331

9227155947610751592822787

30082921267893126

1491551545631686888428

5225776479228919661864

65183671457982179

147321470492465677301

1760175821902225225227591549

271452837809723922160

204715111595101100

361221559497419622162

585112138177245215284

24838919150161414213091208413720
68282430385333527120

122174336
75122363273321429764667971

1815768958807711262328

2096229309219471296287

34910171441140912552106350

1044381063585881542689791677
43612252321209719993143591

37315462286235020073480652

1779161595131313112223255

18222816213335

305710884648185

10442021241959

62025914955466243566668952
29111626262526

1908621797157015612369305

2057771490146713212099288

1969411652159914482175333

592102118261793158323871230
0000000

161024513614
592102118261793158323871230

109197289361307493263

2005129909138191262350

135144233259202293324

132158290255242333279

988215535473563327643621870
86121213129

0000000
2044619138897291015305

2044619138897291015305

6891114167152153199
0000000

6891114167152153199

84335456440386559157
0000000

84335456440386559157

188719262331
490112918261820178023751009

30956299210009431166683

1635598278018111186295

0000000
134133226235216248191

134133226235216248191

488517328282862651524334380858597
58126117158119198103

4718761458144812651802690
412315461254922363722021344157326

1485409306997571328285

126383588560526802225

2159121619142713252044428

159185282272221379264

116438699688614921223

22211291669167516692594375

2466589829569141401464

1427489709917951297272

1689881621150914422171289

163314442520375652243

1245598587616551005313

18212411861172816152697408

121382528522530798235

1366491319110410361671266

90217622632587863163

138614107998810031401266

28410392031176815242508374

12164312039459661501269

121485632631664987249

124412686647592890213

19210031475140713222071401

31410461938175916242632411

2405317067576331017371
3053002

96213283298279394196

141318418456354623173

0000000
135371529504445595271

135371529504445595271

0000000
180389752681510930292

180389752681510930292

149450690778606930234
0000000

149450690778606930234

0001204
3319581538162013692027764

1685398078877001152352
0000000

1685398078877001152352

0000000
163419731732667875408

163419731732667875408

66171862331251
1714179701081517985115831436212148

7826619792481559810097126368481
118177010702571155516041236

21153671281268879

2348031192123510461574357

5942563111127486417382

200475690825607960247

240295510602411110814633062

563217289762449375541

3323747281067675915401

37248170611268511169431

5517191220146111831588496

278136206583352353283

12063141202154166287

199150196311188248151

5897451372178913171716528

0000000
21153945522662

21153945522662

175127176780140155244
0000000

175127176780140155244

5816126384323190
9053161413341500126115333310

69879486516846125622547

1485605657778606948573

27111322521211319843106366
0000000

27111322521211319843106366

163395607597579928286
0000000

163395607597579928286

69354338065563399514856473893750115137797
0000000

69354338065563399514856473893750115137797
991385564366251562684591973

32312302019182216682606636
238010960175271680114882238014819

13383864389519

83274520445394675207

1999081476142212572051350

1948811629153012962200390

1456159698987791340291

20313132150223518643012518

1345079169337361208233

19910782062177717442765361

111518683706644925300

15911171473140113152052341

185543747794680965264

128302395487428702299

79429514524414698231

21611681871170615882438348

9396557376931

49325814160380334995482905
0000000

142724119210539211590275

35118572968275025783892630

29715862460227721703279699
06635138

1115538868467551166282

18610271568142814102100409

216779776616198615190613834921737442834
64699316391528662481662443894703240128368

252825265319

1004878879457941238179

38317564618658

29260311372272527125

59201452402314487170

56429761503621107372

5111574142

89209244310210413138

915131313266

1013586066735911042159

43

44205307236249405134

15211292068164114832590275

1335

4119030631829035397

1248031301104911001639197

7139411158478211235205

875829918957801393204

563717936796311011148

286686746111247

298073625583116

106499814596645963187

32761

34101112134

66451684604550854115

317152019299

23615932713234821953739509

47112168197132216128

58269459348354522139

15211652186189017722905350

1649991817156115852288361

985377426966801050167

214

9944010539478381229249

61218318342280447141

4018923023122338679

4473726285134

16502921282038

112188238279287411237

38771339410812765

16411332144189817492721351

4331752144551362470

88530730770612960150

674389157797321014130

15132

9488585758915

22537164959869

11136415

7122615913

723262233375

61178338355280464105

87476816619579934182

63423571477415754131

964406205955811032208

1044778408447131333226

33721593424286627994424559

43187457464359575137

44

6226245338740065186

157814

3510123233

1131418181222

20771611538525231

12394243283417

32585758599838

17211491742158015452286296

91562778722724946161

1227836213932

121398705695605907265

191

9758311739038451322234

243

7761412526

48231262270289308111

12264727304125

3627243736837859946

6614178610

118382707666578856198

20801351509615047

11353410319418111435196

672328272616

5212611141

6172996612

2016051186105410151395255

67327443416373573136

1197371420121010571769276

56145210231202313149

743181120255

16610511910170415322468291

26113012244197218323099515

13572612339899831674257

65101111113

1154527977896791006174

31421713112

11161611151523

1914436730426743723

1225148978607651308201

89436688713661932192

59319680569584761115

956388758817991333204

388995849114585

13611931991148713852417278

59433679683645998162

477614915213820060

3721229125124242825

4817131228222440389

67399541573527859179

120717232814

18131931242721

83405625470407619116

1559451503150714112098294

71367530444396612182

83105177

124421494459434642197

13369211719709321464171

1165678157656711104212

9659210138958621337205

53511

55221259282248394116

835499287147701376158

1178461249113311121811245

126376604726644991277

65374501544478732135

364210

691811162712

1332666

75263298361296389176

6132714133119

11414953527141

163721104910169051455358

2028141702150615272442382

1208961449125511561948302

1439911732149014412297234

1268481428130111421831254

108467924736803882204

4225338835636153567

1213308076767451078210

1014486446865551042165

12010671811154214312483347

825107788027391104184

611292817398

64164650536440787115

20212992158200318322793404

45127160138135217135

15310841954153814792429339

163775132310879611537323

1118971402113011261842314

12292846324021

89382611599551768186

83436650648587906202

1298491580128111162011287

61199280359287445147

67124177175150233144

85294488407422615151

117415459

73257528450409632154

64247425384435613102

12572412182026

1065858747967441034161

20010791701151212602320520

3910313710514118186

61287286296233398153

21355251527964

12856110359018711322221

80237248289259381145

31811135193

1199631802160914042248245

11289875537945

847321381131912351780165

11852211039429301333227

862421203624

43186361332366529105

167932117910989211607307

5203544336715

148312536475427672158

94672118010768761431260

52389657524521951190

3724030419819131948

19113742579215721063082343

1225698056896051104184

1235929168938441308208

197514

1282036171630

77347501507440754167

2513161921253

69310492591445627136

313587108

95331620579528819147

4816825823330430188

147131124

56127253413

1424

48193239212228319142

65356547477473790127

3912320718420424368

1377871342108810521868213

1268661579133312471922306

8886197710499191484238

1197428998548091315214

56273435370347576139

3161103878715846

296597558911985

104231333315280459249

804477886056011078177

11368210019579271489254

579286446154302385462051171

77304662594541750152

1779251376125912052066320

10253956587224

12463110318738661312209

11610116114

911211682122

1538721424137713222039251

24455878446848

3719447832741765767

9650311249458631276212

119239435379383549203

475921223921

1215379568597331190247

2119771116

1167061427127712271938257

109397657553507714169

762243

1648681824170115182670290

1113420717215330529

6454455687022

2641135

86274511487463649161

3228049545537546273

55251711315

2586469339037231196396

77421497424427681150

3414922919820729187

1387131314118510941822233

6121717115515

1347741373118910441762237

1203647397246251079248

122473707511625

1175448207637151145202

53286409321362545141

215151012149

813261412151

1591718102712

46109891167914487

96417549602560829198

79115203185157193164

21528462618353

3038821047915360

28455240416030

15062810439948791456209

98474639632559868161

19615542578219021823236441

39190319323313412119

15471930355127

1216141691810

48191192267245365135

8131015313029

347113515215118883

66156371315245402165

120436674652619940152

2061969610213152

894918187066451094211

65206259266254345143

9911420019016527799

50911251078711472

14510071665154713582149294

223588111788661

9244232235228

70402657478531827136

1125901383116110131669235

81622208214

91366670685602914150

871625122319

90365586546513759157

12415054516640

4010622418618031383

615411313113127239

271058590719078

61301421380371672101

74253324320252376167

811195127

981811182111

71212251510

65403495436461734147

133877

6154426205117

49252486481387617109

696168838618071248141

4223747947640562768

59267516373384556110

434132

75302288391282424184

40935387454616458539404712

592718242712

875488197697391121183

115116758

17614582484201820233020335

1709051345127010811819306

881113212510

35221719184

55239477413412670101

2417524330418838162

67361567511466711140

5525336840036850492

57971061219899110

134692986910218

1015037658326581074253

3318044138741964739

12752132

494519488697441070131

5633045341337458291

319732110

14211930283222

3120044739837451941

39916022514229020423279722

4446105789713080

1617828626729734551

1648351461133511882056322

2912525025523542853

65313539455508805144

63387676718643942131

11223614

16210931822167315412544332

2912816120112823194

49283527390467644106

14554164447929

291128411010620695

4528237138433656578

8249811278628101373155

1206288277876141121207

411631083

79125201612

1654630153218

13461997610158811370232

1084666437376531072198

976831409121611221843225

237164133112234220293

128383672564571836261

2713517017315625083

2625146940141562367

1669651529126412631952267

13183750545943

1847041381128111751960425

3419828429821548666

2725109263

13912081465129712912046351

163333

70311442383375666103

94329525490409777231

1309121849173015192222279

94509700778684929237

1320938629826837642

106622109310329291427196

84455783567592952199

36119107133126145136

4220834927426749048

59312710571520770146

1333

1258

2513019418117227849

30621563172290526824177555

1724569029237831254388

6137257351243674487

1187811316104410251802199

1035728198136841094156

64390528432475753107

5483132165135175100

257490938614839

41481111011

93309447408373638200

416769768381112

1429831469124712311973298

31113152212204319423091447

9152336505325

17527743435132

2024636041334054857

41471417134

65610374

14374733374622

58121140104128240116

117418720316821020

1577451151118010601582293

1074899138147621124307

814068927687031018147

62553915

81395556546473767252

3017531928726235554

14769010359198711413305

7124323264713

79278318330320488174

1329495

4211012

70239367332323489163

804698198146811181185

2188559

12483641235813

253

1528891576123511932004210

56322

1015207187855811095334

101279497420349544238

20614962485212419653369458

1215978067457031090259

2923945232335156237

18242

6948710088886901109127

10550110898368021234194

19512751993178716862611352

4029334131333356777

17297840386730

84636111511499921542131

9103725226412

675508677376441012189

1257731370105310411635262

73296378400442630137

14122128253513

3915122217325238386

71194438344331550135

68425528606498876169

64229367340334412112

1815029723427541462

68364603533549845157

67258377401360580171

2197982

854746177146301032202

3410119915915822136

2421

63126186214216318116

1163345485411

9567011229569221529228

198312315211711532

32184820

26416422597225621043381473

20111942252186717492809320

19112122050183717392946373

69306659539497738110

65350652503490881112

51024516406

66245532426370616166

5936271260457385286

1508371496131011891921306

32175654439136

5403229275422

79114234204161345160

23217293244290428334360513

1355319418318611352217

27296957644952

1716119317114828833

9070710199108001349245

4010919021217623875

12102825283218

87385577573449759171

804709148368581244163

16310281349146412622008375

7695177158178211117

56288420415381500154

75390601605536812137

22810461512133213092156336

1076071391120411601735247

1495619088368381194301

63369825575633944141

7131912162628

40302447388359621100

309578756211370

1277461376119411151738256

60192163234191256196

655478616666851141121

623720221810

59167363383314481154

298895805011480

79380485401347616212

91202718379

5624843241035565694

216244427388

3511818713410615891

873799918266881050251

6546410778277451331155

77303509547455832176

106764117910799811550287

93278513481409643166

4415720624116831684

84132220172193268151

2109123184

5069123129122161100

71400576710592965135

14321826214134

513812755

10320239444

50221339328331443131

71119164182184221111

21

1610920720318927729

1737131669155013632062378

20614012344217120403260411

119663131410139731633217

52656951586165

14611521974168116572661303

12062110329679071433255

51161316231209348129

431681038

96442551463398648164

76284312351383480148

1310121318257

5323851042336959854

82316472450360671138

1117225326927644950

114413595630561954209

1139861693132612771933250

1225648446957411189185

1247741298112110671761285

91391642610486925180

94159182714

54330485522441825160

14313

93488669725678922213

1564908308667431197308

5122367658854872293

112430677605568958137

2313711207

46441774675634960119

37291631515505783108

223111191810

1287321351119612381927233

18537233446636

1155728866556151092232

16613731990196518152847331

4610725022020432668

3119627023215532294

3103821171916

6233548360251079395

311514817227

41677138

114329646640628800187

215985555215441

12270910708628671398234

45110164192117185128

53112149157111186161

2281696

119361622633574963261

1716131426929649661

2059641336136911651960372

27161811342214

21111

11747310098558061104196

103408693817636981197

77259997

1277391498137211841996218

14611601938152314552446316

2111617116614720532

9969311489508931441201

1095429617797581363161

192382705715511818260

2326747136841864742

34374708607551961102

6131942048341062879

735627646717351172137

40376465466401665122

393414152530

934297436766361220201

14810071723160413602355265

84435766642682860201

3184012112020

13695126

2510148132

94413796608632988143

66281402417356559136

4133215252215

8756810898258471314185

9171327182921

20313662185196917382838348

815427396835761143136

66112189196221303131

84382729545468759201

46346519519408621148

93443728641496888177

11321033

61117186147153254148

56357502475411835164

73138262245229320155

24133233

7162238266221

3

1005238767957021024230

4327338034829958067

124414

2720141433629345049

7195345334110

64171571614

13971011749449741643308

133

6512319018214523788

3410216017415926586

1003849188168101331164

1548431531134712512135275

3710818519117121872

13177758427633

5818947646337458486

14255841346350

3019130435427738263

5985127145129209109

97483543531541777226

18121321163515

1479041349118711841935280

3715528929223332798

20815962650239821163574380

63346518428412601125

15393830477137

1077091319141511581920210

122688132499110571562249

79308347366326543136

285864796810568

11076129

81208319359328578141

83371580497505756158

4281010106

995329039128451190199

24813822324214719923258524

26221763865318830024890427

109316626529489750260

89495122210249231554184

119222520312226116

21586105

20310701480136612281941419

56161552556477823139

12242329383123

49246441347401563121

4886981009718249

1475139927787921188204

1075239608718021285178

90469560730569874226
0000000

90469560730569874226

22513201917181818642727439
0000000

22513201917181818642727439

0000000
1799031677151413892253368

1799031677151413892253368

0000000
1624082545017542335883599719370155727

6309044123104442238
1624082545017542335883599719370155727

0000000
31162925361492

31162925361492

2112303063203152911010
110752135

208123116232

21169911760

342262695562120

16651712237

9101912122333

471116141712298

53157176191195178195

454347965272186662791
0000000

454347965272186662791

78328497433426764164
0000000

78328497433426764164

574934456134204
0000000

574934456134204

0000000
24342811412001644950393

24342811412001644950393

1542811249183901
120931120320422823026199

1931273527151088

51520912110613615211729

34762643956459481

0000000
5092927202263

5092927202263

66966521249926319
174953306300138254250347510192

593130125171118128192

66114730116155513933

5883849070142287

13492209314671903259116092515

23037252835282123

5113817168132

428307488535494709206

59223636563496676188

12542078627817698297

49269374349265470214
5495698078236819382739

12662659877731045

1234490725771460

43203856455781

1145372798961725

94121168169148206214

127814182621065925669278231174521924
39270495121564133581319335110

6157115513332663275918853076

491304416528461681382

173859578109107163

20065199557613622145492635

517767221852212818161475

117011311410524240323051187

162188263265312344110

2102715043603717431364

4625630156335114055218

29679799010190276

179272528340410607130

1712938676670172

55267336828419176

3211612042502282852317

270123111218192166236

2079255182341561169229

3489384111136151918

85394552333046

138118123137121138126

4676693300298487341371

1543643505636224

228422713698124021494042123

124646610499112118

239181211317230295250

257130141197211253209

12866859914896130

359355643496596787162

560763110823730184

3017879950

577878524132991661535

197348638563593890222

0000000
602862910527623084289

602862910527623084289

25529200400
7751354821353434910911

533814878253307987

217494324870192524

3327281106115110781529583
01120219

172365561534467755186

625331736947116

98309513542542725262

172283460523625935
2003004

139232929301325816

3155282223115

11232133099643665244110083278573828731461730
5160493370207554723485366364

98771879552575255045834255
0000000

0000000
98771879552575255045834255

98771879552575255045834255

0000000
904339853894967457667971553

1317871346116510721590215
904339853894967457667971553

131243498510481612237

131340437532423620213

32414682146193918192836512

1875609628217811139376

59387708729605977160
0000000

0000000
59387708729605977160

59387708729605977160

214715169687838814687387611027128508
3297521086118810371636504

1414244
2399841250133313011779567

1578641105117311531619415

81116144156146156148

14245238194245338230345754978015004
67539455176631525013651200535587

21292222267

3319330134025741991

62204295262232313110

1142

1005589318098001349166

140536711647680983210

74614575

6110293115132120111

200244379416306422407

56105532

18423681412

79182211819

22395753555347

107138178226177209160

5279471966157717092001799

31104475

844839017687471150229

30263579476844

1972121120782236196322011664

63233311290275432151

116261316452340539201

19184156499113

91254354322404543194

12135

6114851026

84283514153109179

125327415449355576335

80259411364354488223

4556588

66298634501476725164

6105871489215428

5202385156233110

852340871674645722300

5762908974124100

2023372022343

158714813211115840

43288451492408595167

276961645910091

336

110422550653542880176

1141109818990120136

8102383

18659131816

1467971399119210881752255

35816803597300228824017644

22853145

121311451011

6461

4121352642543657383

65145229

328124253204137158163

1524948908546641151238

26414922236199218002558553

182101591134

6343029293320

116587110010128711315237

1185538087606401111221

3314123726121932652

2824456646345375694

322110668819140

498146022642275196227311087
6437255754215339603362335591612055

31610351829166415762411568

17711342043188015662672346

41119683295301928174114771

1388771138111510261768318

1244327547897261136235

95459681772536929178

17068196012109601609327

50723093485328330224887903

5766761117101710851320507

154727136011689881686275

15162611189709681410317

97258388394336509263

1285309417667181155272

107168190206185287230

27111711883164716152569550

17267497310168811493302

29210691954166916862386451

104411576523475784213

1557531149113910911508304

1789251436147713142005331

1538001537142413312032311

1669521381118411151812296

71383740593650847185

1385249887898021315224

70212164176185225173

1304638168737461163148

159679116810879701567451

1565258349288261236301

28412852631241221203385624

167750125310859541483308

1226591107105310011482286

22156689611147301160378
0000000

22156689611147301160378

23283332251436
20570699010529341306382

70182115152151213142

1124968428687581079204

0000000
3121295860865452614047432262

7827101432138613761170642
3121295860865452614047432262

931767208315872431957591

417281538476408525203

174124284355393139194

1705995119999299

270383828721551712198

433134

220290347378432515187

153341476417450629148

267598027917236017507111520417172861501
1782396661707417581078463624

1339171521322
22612951472134412012036376

16210701315114310311757204

51186140186149276150

1466412966056748580487862851
11593392006765463399580958812422518

352411761616169

21211762231194516372920337

1457531488146211971797290

128320291407336514206

64267392408386557128

855028268048201096187

46272514478449678103

1558201668163013152169400

18072811129538941491339

1747481499108113101728311

1345098758718341263434

19611832399191117622902398

12460910219429451379267

93150229239187206263

419214818315320774

125325672489544791165

28561710669748611306309

1607821448119111771698291

79181392319308426175

1769361568153513212126352

68128135193140226150

13250510989878741352300

16811291927159414142582306

221115222462138190430982136

83230755617623900247

90250234266238365185

1683277747207231022549

22610522354222721542842371

74182221244226264157

127292337571384585184

24812441910170417112402453

110155319424250400302

61123294195258256186

563424605764758954532

78127180202165215142

85975497710139051324273

25165210969719761426685

112299426386307471201

76345605611457674187

97286552614546751246

1455819487558091089190

22413662451230522493016443

66238358352283481188

107539101910698411388210

143177271239216450124

120459486539452806247

229718020518832454

62105191202162213188

1748751632138514542221415

2155517958087021121281

31010351575156514392197465

246624113311859921541386

76398614662530914258

102317436412421499245

1477351327112010491815266

26413872262201619823152449

1587301486119911001828364

139540734671657981229

2208141253113610541723435

2037311384119311261692300

5799182171131242142

111187281266249318216

43260416410374453114

1629541656146414892168307

1626229789988321282261

962132544273258335109
7011860311152179294340581250

195629107513519521314312

8651210809418511440199

344172971008944

5619933133931452697

475662924811287

18721023745086420242402

990407475517329662790873015
11873318829067647499442946512332674

130421441469478652275

41225306335304426107

415242092011

77376604572575910189

13353910239758081266283

63121147134148198179

62140215174194279166

179183139366159210266

814918237637351007196

46134220255154232160

90597117410608671348232

91292445546445662277

104416711643638969183

64131120131148154116

984688638457941198215

1124829326877951307207

86258441412383474189

824309478717751092151

3166151388108410811548194

115740122011879541603366

89139217233175278176

12

16149511759208041265280

1274299137258631026335

55380660606580777181

44201398366425556150

227570107310679371309287

47146226319230338140

8341110287169051030291

10965711329659011483230

20245026302531

515

52260410497400623166

42687294782010391213677

80348605556542870211

200285569528407708108

855349948098491296199

51128140159215205169

7025444445835459080

91195306367352562179

3471903760454388584329

80338591497507756168

74232342346291579132

106399742589568752185

141701107010449961433307

2

82270521441422636148

493441

1196279388698511231295

2488791392134313091861508

1

1305928348017561210301

12222136312117

100367803588744901196

634328427836831125288

5337371161760979051

16423

6396215231214251134

4791200198184273161

364426

129272403419373500380

402523824163524881790382015920

70181690527536794147

662313232615

58190273261237375131

1427361281114410231680230

1918651468124812641962384

52189161188180169212

48409415440378508113

997281216108712321581264

57100164139129201138

96205333294208292171

47108385362321419112

88402712596501916166

112286501449454666197

584207632773233286145411059
78387622640596869155

49175152186132213120

105379550605463806147

68239469460455604166

12163120122922

81329512491445705147

518311913610315294

5714515719114522385

83323665504510940123

0000000
7661123112115173121

0000000
7661123112115173121

7661123112115173121

0000000
10404671421045856111029694540456851239155

18704423413984517333136205748188500
10404671421045856111029694540456851239155

41725241225267152671413

260877811416975195

103210522453221581

107292018361094

13829277516261720174315663345

4169034668186215

2121991104410531394137013678

574198135941230525715849

343206227471443316296

1519

1846938477836160

3781351251152511181255

5425

1656179581156721593

2565728646837146

80217303220239

41235101672822651351433

2290753350579529311498

3219929110810009101289102194

239424710111536201

1652219135222451167

85153759181089

42017536101377520642803

1155267568096551130217

3225803661368751767878853958120977

45007793261064203477369

601122669514145361

318988114416596297

59220114829725826156

9082951187425445861590

2281387419957458631701

2318259559753186

426162502506508483273

2786433272468284365202113531

11343221682333111652892

1663751999354179

1703330486856156

216521815910021167

3297210441922450196

781516102521261

220176307345305364187

118835565657121

10571592447474613

3544911492190109176

126343219711090975836931988

28025342410427170

104201736801492

5276288837847641262301

20261166105127199342

80241711422348

279157223621833912321900

2465461289568178140271

862471079538781630224632196591

188152751670357154710377

7907100020346424091402976

216717091118104208

230181155212242270206

99691227479895423454788

333168175124246160202

69213134131147107338

391159190234269275259

9641486811026363288

163425058015585133

4327357707463305

145162298736977911135488

751416132110208

28854507917952239

3308683102203175211

114191115541292

2610629386534552226212450

579465887142450529704297199

237320567497546763235

317295405373387513205

207310409362342520180

2754956435947148

32047284851547883071110504

16733547643814685438

54496109246113512997

146116526762512746

8329130210511064717960962

262402825721715984265

495149168182336103348

211751078662236118449

3211119714712974190

43742200121372176935514

4729394193269102507

12032372222762982651446

1034412433261363145761151

34792157166216180179

120172453418391578131

3888511221379191239632

436658211913873373

475161152279316185351

35550153857184148

2735

2414298887437831139211

342148172310170272463

6146631069126099813101048

49612679136191120245

1693118514257284

21349657010156331

5986229199358971239314

52931073870104610061230808

616404602486583773386

3995238496560196

6161979233

3415230473725324

1063329313722107

500676034479153411368364209279

81331311242288

504844114415323051957

412332629609768891273

547347

1073310186293273335657

245363471403477697196

1352123324226131

12125141627962

382147124165260146221

426484171589477370013116

79523011341515452624

1457205953471671311359

1184142344040113

683101030653

2375434605553153

558613311055120510218267200

682252761311

1412523185735182

561423479485506555282

11713493181356489493809

1033712371985361831081069

53898106147186157217

1816526848731254

42

47969779110876216

1344344327821118

109151925391291

924951292714103078418434712829

15114215632689723732220833219987469
51011053

3588457838523706367047548635
14969209132601022677211643088287194

2985198607276401036699

212309255252263312392

167218215205227265367

1647618434870179

17673310199808061377351

5956716923145

124399561659587809251

464666608588461625920

1665476946115691040323

1926327648107611070336

6662134740793

6842882052372072258489

17154123224741

2658861838162513982405525

279473603576556902573

732319164983992420255622143

195445339401346669353

787216322419166

5617502132802183301283

37872025231320951852283356001

164241207139124187786

61128218103111124127

1114362113

141332384494380551243

2024141116842

134242471370337531235

31810121770162514822309555

3702911351801482191134

121153142176157195307

873326252718

1032105997610329541407998

8931835314174

226439717575491902397

14064988710549091312272
0000000

14064988710549091312272

0000000
1999711488154513861954480

1999711488154513861954480
0000000

0000000
1999711488154513861954480

1999711488154513861954480

354610692819592
898892371542215577147202079314243

79043768569261616112692312251
131202293055

31101432111932
6655301641844853510452812528

82125204199192278108
1122285519

378712210412219040

34366087658349

0000000
4134150246138371062

4134150246138371062

0000000
30367168368867

30367168368867

174001103
1557381500743986641393

14011414015753488181

139236460586441553209

0000000
602103166228265210677

602103166228265210677

62361539562432647129
0000000

62361539562432647129

3398141419312079275726391027
3001112

995908408187321266168

1954162757663620

31017831029118519481309237

0000000
852245257481424933

852245257481424933

12367511488128699916129668
3002002

58382795681642109085
0000000

58382795681642109085

11753696936033575229581
1081054425

1644821010976107291

9853124554742724029255

16118155910

0000000
10495423962493248527137751900

1055329788509451477166
10495423962493248527137751900

51194437398437633106
0000000

51194437398437633106

70102732722
0000000

70102732722

2917635734731547371
0000000

2917635734731547371

17311372060206317272901313
2110913117320026349

1257951479147812461986189

2723345041228165275

0010421
1216048719157171296185

81390582532425847111

4021428838328844773

4336558454150777482
1308241376134911731986266

2241101888515372

654186917205811059112

0000000
26495455809954

26495455809954

1284258415
0000000

1284258415

0000000
82384672585568891138

82384672585568891138

20211992246218120573298480
41412830728675111493

279618317616029454

793527707797761188227

55339463498446702106

4830656754946871084
0000000

4830656754946871084

186813511514418063
0000000

186813511514418063
0000000

0000000
186813511514418063

186813511514418063
0000000

0000000
186813511514418063

186813511514418063

8248145939085140
0000000

0000000
8248145939085140

0002000
8248145939085140

12102610131215
0000000

0000000
12102610131215

12102610131215

13165950214944
0000000

0000000
7162734153520

7162734153520

000000
6321661424

6321661424

19181017123
000000

000000
19181017123

19181017123

1010178211831
0000000

1010178211831
0000000

1010178211831

0000000
2812251318527

0000000
2812251318527

2812251318527

1595942524251878189819916214
176287959520125179107283120569119272421836

0000000
8152148022603724486201602930412531

7185163189142180175
8152148022603724486201602930412531

85129181145141165168
0000000

85129181145141165168
23423829394139

17143524334137

1693141162649

20355135293424

52839151413

41237996

82389718582899155019561161
7025241989

0000000
13415625298037

13415625298037

4212212814511420681
0000000

4212212814511420681

0010004
5668163111103149170

25154532307047

3153117797379119

5613507341911669800461
35141682232824

45265591422341

1482562100504248

80264994485456

65377580657251

592076112774068

841793191227294491116

452382125705057

0045379
8096314241236263197

29357672496378

20311259210911355

313010972758055

36143286310259296131
0000000

36143286310259296131

11123830402920
0000000

11123830402920

17651141028112555
0000000

17651141028112555

615519987111154102
0390030

0000000
18165118162523

18165118162523

0000000
331210437669133

331210437669133

0000000
10243532293546

10243532293546

486210669846979
0000000

1002000
486210669846979

13284932131939

34345735715040

869324347723526345648963265
40914966

5039448227308277229
0000000

5039448227308277229

193199921091221120422131890
251871479080156264

83749532369350502757

75928531365388663776

1013589939738689293

0000000
564716211310617277

564716211310617277

71230387443314488186
0000000

12124638233954

59218341405291449132

386214613311813265
4959281657150815151740877

273753501345761

30447056528446

5616422023823424793

49657129504566

413574103325880

473277525710156

2267117113819866

16555633363028

3813921119918829869

401618418913821060

29226542634842

33319959686462

2915921421226426883

388398724557574704560
6512512236

0000000
18146137335937

18146137335937

121128220185174179175
23103828193530

31

17283238314130

14134328222713

19163117332933

26405843251928

4448

19171831402432

11101400
41408278607982

13184731293328

27212546274654

0000000
32465731484950

32465731484950

151132238188199252168
367146711

1595330234525

45193926302831

12283436385826

28244029505922

25212032362536

23254521163017

19335433486342
0000000

19335433486342

48344826384070
0000000

0000000
48344826384070

48344826384070

0000000
144332611531509817237

2053210
9996176120116199121

29263743324032

9434514284130

28184521307430

3194439244329

0000000
45236435411393618116

45236435411393618116

0000100
214130309214202220234

0000000
83326047564846

83326047564846

0000000
12187041384130

12187041384130

11980179126107131158
31145326213535

32164635262631

22173334303648

24223014121030

10111717182414

1035228242136
0000000

0000000
1035228242136

1035228242136

37407347616951
53159399168891609413220199396352

898126219912129152122211165
47298368148681395311627175775560

344295815311250

1517831211108310501673235

54245940353073

215210453388294457253

8054881021028497

92286484520507581167

105901251428812291

421401261149813790

70231598447410624113

139253492455437674184

106154274262217391105

47101134129114171100

180205331314245446105

6646171210455771

5172536493752

4632212

1637001387112610311576239

130140286190138238153

56112167166149184142

101253420428375564156

4617751646743264271

227451021935110273

237396610610474791231

182280470358318531136

6710490103759479

683247126365845

34306751426666

290230502520421743140

255383690606505798330

94112233271216234112

1014749127316901094156

82393681581555837116

58751151137994120

84122107907912679

120416806769692985163

22

1459111029111012496
4005201

754055111513352

665155175579143

101304559474456758166
0414563

26184639303643

5824648639637867596

17362635434124

9873010115
277584125513069461368455

5425263262249877785

44195837304033

13164554273633

2511819915115221434

725010619998128100

26487092507859

14315242193239

20428679625267

26122423204324
0000000

26122423204324

0000000
221580826111038

0000000
221580826111038

221580826111038

0000000
54204539483354

0100200
38132520211741

158543316

5547311

6469556

1293468

1672019271613
0000000

1672019271613

860113302866705141519756346922
0000000

860113302866705141519756346922
0028407

857113298765495057511655576786
643649683646498

42992361016785102
1700000

285919758274652

13333943403950

116981773193436362188947
636112907819372087186517483545

27244162118

1410812116

3324712267817353

7150935321521326051

31224920162218

405160694909593142

272852122181354

33651910113148

118274221373332

60140343383425705

21594644342338

1947211127715411867

34494435263235

19602935274544

473818876747

1205351222420129

319723442424262

38394826213171

3217276431822

27202160759312028

42017647280286309337510

303174342473551

827441891611937

11210792791417110

11384085212

225718448523031132

37524835425333

32351713295843

4499111757311490
0044022

15256138345745

29744633395543

41375739295141
0000000

41375739295141

82423217149161221163
634129520911398140219661284

42377641607241

196465513464108

293612910410915241

33416848707463

11167460325753

154214459727051

3050199899416569

223966422616128

35436059495657

30274733313546

30776847496173

97317118816817345

23237530537037

30305529587052

29267244466635

17465939264435

18305958405670

14457654436537

30155244353758

32167234385732

253710643607438

29598153486652

17385735294551
0000000

17385735294551

162190167136137111187
1368197719641239158714841575

44648057574834

109235557463281

33604761414349

784168560646285

54424749426354

44205131438065

736589533843114

38608768827952

685339415533132648

6454107445576180

29517777486554

6054105815476138

12794204912217290

19248745525139

273752224313337

32934718374439

61557241654447

506690327867102

1961185159656980

3041119767777129
0000000

3041119767777129
0000000

3041119767777129

1071921701516509189923665914716292151
261436183522138

61064115431002100612471165
11287619

146229430331335454245
1128032

4040010
7968132106130135126

35558562709267

40134344604259

66160296217205316117
8472591115

2111818015414623453

13244215213225

24146723293924

14826316
361307920537494641649

39255534445257
0000000

39255534445257

0000000
282035515510417786

282035515510417786

25193436302447
0000000

25193436302447

0000000
29582532372967

29582532372967

239181443278273356376
943535321922

57244628134127

141124302170178245260

32296045505167

102104191126170146252
0000012

20324631382647
0000000

20324631382647

0000000
19202712353261

19202712353261

6352118839787142
0000000

6352118839787142

1065561636014930179723561813447290848
301844241951193529210380

3020107
6766794567846120145313200

6000000
57255392245169391063562

1375140135370619323227

43443998914632074335

279601196766704907
10382553413302613479631

2896742543455762

3919145392575

43253113242776

120322321283164

23338548767603580

134

341455353379163

40251020
200392767685626923624

47197422349337535132
61201430373352557180

144624152248

2

372662536653146
0000000

372662536653146

0000000
284889615383107

284889615383107

0000004
70117184193154230171

4310015216612520399

27173227292768

0000000
17243836285947

17243836285947
0000000

17243836285947

874987587695260
0001002

432629284022156
0000008

2391519231161

2017149171187

0000000
442358293673102

442358293673102

115381821162322313316406131629
0000008

297262774321710
114961807158021993289377131509

57561356138167736

64326364219288716825941

637716331734832467

80124414610879

19562471426051652361353

8894971231171985244648

28231816915225

60223438618215414648550

0000000
421443322729112

421443322729112

0000000
1132118281847

1132118281847
0000000

1132118281847

131121122157143106393
1102187

46834439442690
30571210211356

11151516749

511171316925

572254554337192
0000004

33132328199103

2493127242885

271524615535104
0000000

271524615535104

0000000
602012873929792

0000000
602012873929792

602012873929792

75932054342330702654433628036
13946681176271675

188138473793441320014626
4291091697243421047

587128116861971612594

7241093721891061677679

83958492120195

58292253846101111

14223567014094794478368
55731624261820192179226422735

1516165502389

18101210151974

793133225920184

2621122626969

3179810316

2427686559245694

78324

22334

8728

7226188

118129123152

382160219198243196598

1606737394025508

2718313725

963039384275121

762748161329723

54569534732642168

1381918761261

1553989764753485

17352338

6111235

615971221321611303258

17172316273289

1432753473849377

4671003061231701752192

59361481961

361134354428103

1409580143108161228

512231412939168

25032265522271025

75190403337285465188

250691338710372544

0003001
2971031951611801971694

26131814231882
16069153113154163552

198273232058

593945717175280

3221910184181

247441519951

0000000
13734424526341141

13734424526341141

75770826941216714232321978100460
125613482120358192021

67107272534842678664082
6330866902636421819302110525764

7579675694516117556

2982317153916174

5917782408440108

441519222820104

32724134719152

41564559767115262395

25208142011111

98529165236001485532707

214298381655496507795

47650494912119269

98267363256210324

402222313517115

144826118875151172608

18850176642166010191052351

9018637281087186

532311425815137

4896465653911153152708

18882699213227030726

27152531245013597223664

898102331305505131021

60994391509326293

511946422744178

0000007
57406574773344638434617

590665214124325370

51505914251932205934240

25148333923105
0000000

25148333923105

16731706298431639
52047077741943298565237045

1811585150

17771887811142113203248

18730357994501220

304144658367666653530867

141349321

0000000
351938312832167

351938312832167

20248106355763741
20136511485

1241858102228462

581742202431194

6213410112119
0000000

6213410112119

0000000
1062224733853402309644663967

27424670825773
0000000

27424670825773

1035220533393332301444093894
10335857586108310

129285557576547723395

31134150193171203139

193475600491552930631

2087130115112157104

39109149229159258130

64105175185181278129

50298427408300493149

2451351521532061881299

5496128170117211133

54284601572414619258

53162185165169241217

4921478111665841514845147560928100153
0000000

37032747025205146501123
4921478111665841514845147560928100153

0000000
23334855353331

23334855353331

0000000
300221234185270254327

26173031304160
300221234185270254327

2

102

5142

648131798

5634193

1111357

3817112

115413

6313539

225174176122201171231

7652

257017422817405157719056670067560
92593152397874972553172

0000000
6948147421201061

6948147421201061

0000002
6014274746343347

25444626283286

359828483511259

0000000
77519360434138

77519360434138

20876070120698085410631872
1172452329394244743584331812292

543084222524886

367205834

86032894215201167205325

44776673707860144

7318138424857113

1011882227292947

11020368775773114

29205181081331

6016249393622107

4910553554073125

392932017251555

16843381014

300306531305955164

411172634484171

262253255

16996293201813877

952725637623874

152230771819453573

2833698218

38582819233366

176241541815332184

1484404035351773

363983225122636

498520515829124

571302420321877

28726770775171145

2431572115401358

511604345322089

1252892823414096

25596512

181177333940193896

84120631251338

592625340293475

1807725228203442

512605336323143

4316110596876785

723731814152665

7013352371936101

871292875393487

1219582252521411173953

146887275416254272

1431922937192855

7815537453327107

2645746613

641912330233449

6730243493949198

1391594156332750

421203939332788

1

691295738323385

8423956582546281

722124738587595

243949566

921803455432167

56531596965630173

11830554653254151

23498193720

41925636363359

1021993441362372

651364322182442

629575920294454

688975825860158
4051002

1034192881638

545551535044118

6300102
1161228990979379

44453128204023

66745862765354

361415456124279231688153041526
798380127415023884029719

17326458577415079370320484

36218581508221861913969

722333924114493212347354

5561202515566402450744
1966222212847

391603834295170

16512064155593677

1169178534930125

341113625322966

452263942353579

5213817212613121486

4917750754634154

37113163491340

2131454531473585
272718121872191851531195

20329060565456350

11173919252235

22001304731135940725

112445103808576362
3604006

23741711151378

8636586657063278

31232721171927
738477645579109

26862411

3194521

28382124193655

211565167

7573728

2954659976366297247221549
152419132193456632153

645879515

23328141821057468123

282842632272284

6325148

8703983314527251764

5132127549284846

88186602711112123

91170551119111333

0000000
23934652709970

23934652709970

2603111734392919612625858
4120152522863

2472767012613176414

231582125877818081785381

10143321232619
0000000

10143321232619
0000000

10143321232619

0020130
5391233102127187102

23184726393550
0000000

23184726393550

0000000
3073184768714952

3073184768714952

0000000
2124224

2124224

10153521232614
0000000

0000000
10153521232614

10153521232614

27307745478977
0000012

9173729264436
0000000

9173729264436

18134016214439
0000000

18134016214439

22718351565707234760413785296130896
494115522011194144020411353

7213522283842
0000000

7213522283842

137124213143178224200
06159344

19165424363237

23362427324634

22162220393124

53274121403735

12132525174637

8103217112829

0000000
34255121193434

34255121193434

14223720193028
0000000

14223720193028

0000000
25245439373839

25245439373839

477511367848684
1313276191936

1017325151717

77131315714

7141910764

523132315265

5191013118

66316669748856
1000402

25103535365426

40213134343428

0000000
18212837343626

18212837343626

28305942665554
193170363182226328250

37196723343749

33366133276727

71221

16144920193524

18203219252829

2522244

35225315436345

2424332783717

78139155160128131142
604630952722825944729

19183624385434

23311421213113

37193838303236

13117317129

123119638

27234842515041

16275420213521

84781

23115138403725

9213816185318

65678514

1930249282717

1955333342834

32404326402724

24383645375839

136115101712

25292835564229

30345917414121

32264241474841

35314222364133

21163711323725

114115

29273927262426

32163334337337

14223630232920

713234

3332221293051
127170278221224280282

18134123373436

7131104712

14226422293118

1543311172818

1292234234926

17453543395238

982520231742

1072010121518

22161527111723

43527860465867
0100000

15374836173329

28143024292538

0000000
32264838423243

32264838423243

0000000
23301622321733

23301622321733

11164931435254
0000000

11164931435254

0000000
49154234152533

49154234152533

0000000
126231541516

126231541516

20243634232638
0000000

20243634232638

453210146506757
1022072

22146622222539

22183322283516

36302916175130
0000000

36302916175130

295815612115516564
14121710202222

4162112292417

11301189910611925

0000000
45311117520

45311117520

0000000
14284123586131

14284123586131

0000000
57256560576041

2661922191616

31194638384425

927512971120113121
561328109

33122935582939

12403519304928

42175215242545

1735425176038
0000000

1735425176038

318524333194267328352
97667942618773

241181193

781394

2713281682322

15198810142640

20201022232525

19155012472429

2543301925944

42295127435146

64917637425566

0000000
29224326373930

29224326373930

0000000
10161510222820

10161510222820

0000100
43557644555337

24383829363716

19173815181621

17112317303029
138180309221245323307

21473320182153

17322120143232

1692932211830

711421324540

3316717305

14202917191334

8182737272618

149256193324

21309244487542

92841607674125118
44121323

39283220153743

13185726253432

36345929315240

11294180109144198154
2033000

35174125394839

25332725184225

7133724314821

24183416362343

19133816203726

0000000
1882420173322

1882420173322

0000000
1387032404735

1387032404735

35116132
165242551432503599236

15192111112220

1272741121741

321728111101616

3312533828227944247

9273112232230

18215630204223

25112019232121

18101920241436

0000000
32728549507848

32728549507848

0000000
1813513319304

1813513319304

18333424213837
0000000

18333424213837

9412014597105116106
1227181214206

32253529375141

26345042362532

81518710917

161924781110

20411634212627
0000000

20411634212627

7969271954554626367306314212211668
1875531882655140563177932717

24204624234319

17914482495132817842732755

625522111314720078

45428150576243

50146959538940

321782507310750

3036128539512939

463223110013215152

14625414910484161126

40571239612514885

25307957397642

64328766636437

324515910213014856

7467116457112279

4711126316915923665

408025810716619898

373910149578147

63667766787455

1386311387114123115

488720514012114169

50396839608239

7495379180292310219

787625015413520586

2164133789913846

363517379718958

1833126665513422

22316128424118

70230537469396652121

579979435856078098

391441226212911326

669038815720333974

2216556228427150

243580568311552

38997998450550526204223

3168437216218280352

3990961316939218

418126511313922157

725598826710649

417925310119520674

23119814741241833745262524

40427437426468

52519365576184

586021811614116786

669022815517722660

221122419347267363136

65847049548741

283610454567762

201337846917850

7136110696910034

5392330168149196136

733310847589341

31166538406649

415162542351272365122

30581489611816854

1

3459330117167236154

11380160639599123

594411981659272

568027918815031264

299124410514523374

679431520323526469

26312621184418

589673168776612145

80839256536066

687834618723131281

412498955779745

7610412677357671531117

9464157697513092

124258422368315545116

83537748957251

6358218119168153107

134910982628734

31466240395334

94149443238282349124

1065097467810764

4219569340547260586

507823712514219258

25327742599058

1186717613815614981

48507138505268

73127761464830

1

5110625115816527588

37275332465963

3211324113022220963

47722119916316374

63304747423643

171615711411114642

7110387768810087

43177138484036

16348332594457

371503337204023753002306

21613438266129

5541108628710288

122

47621158410819946

4694280122141275121

5375184137135176107

367722211212021085

68103195115152242112

28135724342418

80112366151250254117

605410086759457

4518541243837122
0000000

4518541243837122

0000000
14222945363149

14222945363149

0000003
47515233456868

22283220323734

25232013133131

0000000
16114518194129

16114518194129

0020210
1255915079115122113

20196214334030

84314730394757

2193935413426

26312722314836
0000000

26312722314836

0000000
22326929447142

22326929447142

0000000
12252212171533

12252212171533

0000000
13112529283831

13112529283831

54221613
965113711012313179

2095359523827

42353028354127

2935221354612

342284508318396494428
44278566527547

20223820345256

28132726233843

26404117241743

25224820414932

59355826393946

24183915325122

21124221171229

20183318282022

1718814205515

37283619313332

2299226

19294447535135

52518970678072
25293237374840

21225132272428

661384

33727373535162
6653571

983425202122

18583445282339

217118284166206299217
10182217143224

6195440395231

1814419193128

41434437586329

24185725413336

37123118136125

2643520222744

1094791599610917581
16164729413326

1592621142920

40355022275020

384193624276315

3049261121245407672012
951711686081246239140911381

202193418238207

4427611823526540

6046217713511950

564473610072653

1832492823

41131722294025

14332545673250

1566313478

18340854640481021

29711514453

5504830243962251

15263276745250841181

8816442781231296

147471348

775781318

12791826010531529135

1152511142231
233245509307402437323

22302322433721

20232225262719

21314935333548

40315231323542

20294437545323

19327843626937

42328624496934

20278257676537

1854822222531

0000000
7112625273220

7112625273220

9302015402830
0000000

9302015402830

5759122427710572
71325711167

176131111319

164295141127

17365519414729

16234735985338
0000000

16234735985338

0000000
35423121412741

35423121412741

157138285216212298217
2563430314934

3243016193213

2332925311425

26252115223524

6123417123220

26115114354418

12134115

14103534183312

661525192519

17191227122830

5116931

59171647

1000002
46571261018811989

20255861385838

25326840506149

77240536431457506124
4002113

31293536472446

2715946737238242047

15523421276128

1382726162618
0000000

1382726162618

0000000
22184824353035

22184824353035

21355035323336
0000000

21355035323336

0000000
19350711268729801334278

19350711268729801334278
0070100

149412748640690950204
0000001

74343605557574842123
128133017328

4532854247650874185

1775051496930

0372601
75691438311610880

27265035485832

48408646625047

0000000
449537123228938474

449537123228938474
0000000

449537123228938474

0000000
1316355463354201403051473537

0000000
1316355463354201403051473537

58197314267203275307
1316355463354201403051473537

0000000
13154022203029

13154022203029

0000000
83110148285726199

83110148285726199

006172566
65178270185210205192

289090848687101

3788174849911285

50822259212617799
0000000

50822259212617799

97183321180198290224
28131110123

244911533568271

28293841414639

19513933365450

244611662559661

0000000
41791371069799122

41791371069799122

0000000
496016974100121112

496016974100121112

5385366263204253118
0000000

5385366263204253118

00201301
66130263157174227172

3849109647410691

2881152938712180

77152232154147167249
03001736

30427255435376

21617451445987

26468648435280

13324937516233
0000000

13324937516233

284210852657072
0000000

284210852657072

0061152
70110304206220274197

294712152707084

4163177153149199111

0000000
1199871110600648839309

1199871110600648839309

30112199105102176114
31771113858638591163830

21346942526286

3012022516718023365

22402151026314496

3010695557710568

294511042565657

27146030443526

28317780547452

4

3676137768611587

19354732463950

22347062506973

23647770495556

0101333
51104192173145171144

35528873748979

165110499687962

0000000
269518091105118102

269518091105118102

0000000
40202522394386545127

40202522394386545127

0000000
247323622917021850

247323622917021850
0000000

247323622917021850

0000000
61133710152224

0000000
61133710152224

0000000
61133710152224

61133710152224
0000000

50103101
61133710152224

992014810

47476101413

6956151421
0000000

0000000
6956151421

0000000
6956151421

0000000
6956151421

6956151421

0000000
618368071096267578090331516

0000000
618368071096267578090331516

618368071096267578090331516
0000000

821141461511
618368071096267578090331516

753830173123
2009392332213918953057634

3091180190148247149

1236911926166115602466325

40152188258170313137

0000000
41027204763411438795961871

41027204763411438795961871

7513241710
9839432617667370887635979968717959

8885422887398568895613939701416812
101222181919

0000000
354173405377349343691

354173405377349343691
0000000

000000
888101111

000000
888101111

888101111

346173397369339332680
2041002

8211217191227
10378126139164115203

6610722

7292191127

82624611

3615117

5104758

1524823413732

21514151211

511

1231357313

15142108819

1719526451846

47113931463047
0310000

2211211131019

176158261014

81111271014

626179734
17979183130112138361

1214111112028

2461312141915

10119255620

2016161431511

30555123727187

153119978

1973715121322

43252515122436

1554568174967
93424983452

2422

15324

51

312134

23

1121

4

1

153

0000000
29498784759332

0000000
29498784759332

0000000
29498784759332

000000
554894

554894

0000000
15295352385713

15295352385713

0000000
9202928292715

26214243

5517314169

17373

1938643

0000000
89671597

89671597
0000000

89671597
0000000

0000000
89671597

26661173

631424

784100721182588180024591201
4327131912

3718644940035344384
0000000

3718644940035344384
0000000

3718644940035344384
0000021

14246745277222

54124317327

1815837031230933754

0000000
488261467381404441434

488261467381404441434
7110200

0003101
2763734393522

615210104

43232213239

172971528

118406641746283
2077010

1214376

100282624594267

33246845

189485

32113936181824
0002001

17733231288

15461161015

813673341465
0000000

813673341465

252162190190200234251
52312947393532

2

2381717182814

449241

42284436366657

1631514181319

54411326383254

331

2252211171728

91014911916

1598834

38473

87156111217

411434107

18123017162119
0000011

1231878109

6912108109

3203040
167291010176

2272352

72113572

41112212

0000000
1098204824

1098204824

14113082
19649510869819251343459

48214159226185255181
0000000

43195142195142216151
0000010

821216148

312716212922

32183114158115172121

5191731433930
0000000

5191731433930

0000000
35162291012

35162291012
1210101

119113510

12611551

0000000
26135237304423

26135237304423
100131101

972313111312

24611

56281712209

1182598586837011026241
1030120

9461412814
0000000

9461412814

0013000
18192721344020

589617115

881579228

5325877

0000000
18232626241622

18107916819

131917883

1816141099
0000000

1816141099

2710117418619720178
1186522

994218222623

5131026211515

127811413614915838

4410460642242375098
11192539345428

205751033433058040

6193518325823

79363127587

5962114819105213212
0000000

5962114819105213212
0000000

33162851040106133
5962114819105213212

115172081710

991618254238

812604817

8105347484014

0000000
17315712110214894175

17315712110214894175
0000000

10000010
84836562814884

0000001
52745752564058

28452729301927

1827211211914

6228125216

0000000
31981025816

152541579

167361017

274117
000000

274117

0002110
87675240664584

0000000
12487221115

12487221115

51172275
75634431433369

12226418

1337310106

1033636

68245312

1126124615

1010897910

83337

0000000
209223440399378546289

0000000
209223440399378546289

209223440399378546289
2632842

0000000
326413390939465

95410169687027

2554414

9223135

537416

5573789

14421

176963

175153295304272448215
16104951606219

121142124

19391710

7135143

33123673

212163154

26381

12155446

1914358118

111126105

822574

469132611

5417164

17310152

62893233

3116856

52435

21131010162

4547103314

16851028

13199151616

786484

671611663

22101614195

231013545

5845373

5412132612

34121133

2132582

3132415

27237151

25112612113

233342138

3712126266

823711111217

0000
9357

9357

443429
000000

443429
000000

000000
443429

000000
443429

443429

7294405247022364968582369290014345
160367760858738921313

0000000
16278803154761450612812204573255

63211541502395592127
16278803154761450612812204573255

29494657529033
0000000

29494657529033

35609193599339
0000000

35609193599339

17313922258199717812963419
7244736245122

3119923930322432062

7765610599458341464185

585139137136991128150

41234368413347541113
0000000

41234368413347541113

0000000
27266251605550

27266251605550

1914538636426957143
955529439307521393173

4734449442341370680

14283881346434

15352562365216

894136916506241062143
0000000

894136916506241062143

63115218200211240119
2040200

16439472869944

17385467849240

28346661394935

0000000
935103828413741

935103828413741

5042456853540185282
41023563972385234545174752

5619531034630543054

9443572466133

10193939487424

3517033431832035564

219021019716025931

5525130535230039156

4734138366932

4928650950449177962

3829065055748673475

106186766114930

1145819211212434

2517027927623134158

4151446304828

3322351140442750889

66291480501439708167
01900601

18541121089113943

48218368393342569123

3255540
1247131243109510321654211

15707769729354

8158510899558961467105

25567266599052

0000000
234715020312919954

234715020312919954

1649831960174715542414358
1463766

3528046745433660761

40251366360351626109

46212533439366560106

1025734850469

3221151544344456967

13302631234517
1508941620146712472165286

22809172597029

4134066661653995868

4039075868755199397

16232920253544

18315041506431

44392649560517852118
0000000

44392649560517852118

0000000
2240811067512552

2240811067512552

4419740533030052853
373821621381253476031517503297125

124678119410859281519177
0000000

83418694619537900114
2866107

314588946410836

64171516810

4436158350445678461

4126050046639161963
0000000

4126050046639161963

570309162405364503078171132
129888807212715

36519303991341131784751702
3820141736038049974

3517429930123639264

4022862449241967294

2921873145551759943

316711912610218443

3425650642337566168

81434702694588907177

14353541519223

4529049745646168682

18276163495934

3131544140140177092
1487981663145713692389292

222

4417643538529853656

1

98339732434950328

2916726622622244268

223985877110028

11183933263620

45265498416411550123
0000000

45265498416411550123

551329054445041466771491037
11117592

117016214314219742
0000000

117016214314219742

14143234183817
0000000

14143234183817

324142115587
61395709519575877142

8155255537040

5035664344350774995

686008238287151213155
0000000

686008238287151213155

4418221819916927186
0000000

4418221819916927186

33419943400324429594430564
87524739710610938130

584607417146191007107

784378187157071032112

1139101963

4422

17443980849431

26837673809168

7923923843095265

60341582554529736106

183489678411429
0000000

13114429323714

5234538527715

304211495679554
219313459235022122719140313374464

22413673567857
1001000

518824264315

16232848303542

3516323523222536660
826128958157481302159

2720538229727752752

221221164

17118122010

1821925227121837530

635583

53436698814673987146
0000000

53436698814673987146

4728546042839764187
1818111241124311061942310

11

5421625327922640890

348617222516836664

4622435631131452768

3107205
25817712945272423733905506

3920729232728742363

4216426427122933788

7374033274225

3631647736239160583

3211617713514520393

999301695158912922295149

0000000
172785757613629

172785757613629

1188901482130311551820257
0000101

988491406123410941733215

20417669608741

4533850348042671170
35823914235367533835704672

664499137487231311178

5011013415712718293

2210012911712117258

17513942556217319863328273

9326325233916
23113842414198218512887482

18617550567925

28129961129818663

22461391027113135

525169407497161151109

10524977529343

3825557041743161988

3626843138733853376

18255163665627

0002000
7953110458467361160201

554609247366521010158

24711211088415043

514234114157
1738571768167413172328306

5317435037124742373

15311051227911849

100638129011409771772177

101618114710649551549198
0000000

101618114710649551549198

604407386566031026123
0000000

604407386566031026123

48107158199160223147
15363660487732

81247985

15285451335034

4152747335933

5152627252236

111171277

0211101
1188831722147913692157282

4955710979058771375139

15345339596536

54290571534432717106

3316225623521534968
0000000

3316225623521534968

1814720922021238558
23114562523227522973689467

4216122122618735679

419216316316227170

13010561930166617362677260

2569061340171314521979262
1002200

0000000
4129647953443166672

4129647953443166672

214610861117710191313190
28334194568514

5925440444841553968

127323416635548689108

17699733158621484413169211933652
1022243922636

612152016326
1196705911587107419498151862496

0000000
3318823528920230449

3318823528920230449

0000000
3828072455155573585

3828072455155573585

0000000
805258576836461063164

805258576836461063164

39218853596320428744507763
36188402284304508103

11406287709426

2515543528627146048

145640696310336

17621301129014639

23398266559141

4125135033328045888

2718340732227441962

1346351363125411371734163

269311112911316751

121018615312317742

26821281099415064

0204250
90404562552455741200

25881331027914666

65314429446374590134

00013327
38350572543472663104

3031752947743962557

8334353303640

34286426408374648112
519341550264899427871411125

13325867629537

289816216915819173

3275107988714346

3417524424720832781

2717120630121534497

5235849848546970799

3724847637934555652

51439735665583942105

805437377816541094140

716158628617361314155

19871331067318450

4128838233231459678

563265242514064364959441150
1822236335133356855

15303859497134
0000000

15303859497134

124472769210763
1045168718267421142283

19471221139111150

2330447346839964763

144068596311048

27731151017914350

7457278

2416216171

0000000
6151118193217

6151118193217

88496683742572986221
3213037193824

14549471639153

26366866427834

2920425429026642666

1618123727818235344

1805018017336911153271
368911512710016689

26326671637637

2422512261616

7529652643844477279

271618162510

11424248318426

6331119111414

1528721484133512431992269
02264122

299110490839567

1117561327118611061792174

12235153509326

0000000
2914257034437255144

2914257034437255144
0000000

2914257034437255144
0000000

2914257034437255144
20012510

2614057034236554028

12465

111

121473123
0000000

1133262
000000

000000
1133262

000000
1133262

000000
1133262

1133262

000000
214161

214161

314211013
0000000

0000000
314211013

314211013
0000000

314211013
0000000

0000000
314211013

314211013

6020101731119965
0002000

481995651049549
0000010

0000010
584320313713

5721129167
0000000

000000
2718358

2718358

339487
000000

339487

000000
122822206

122822206
000000

122822206

0000030
43115245735736

0000000
2062223343021

000000
563839

563839

100979182
1561620262712

3211224

11612856

1372

8581433
000000

8581433
000000

8581433

00000
76176

76176
00000

76176

000000
8241318216

8241318216
0120300

42510113

41085103

1000001
121667416

433449

713336

0000000
4456862050147315952000412

1020070
4456862050147315952000412

317066691019142
0000000

0000000
26425445696826

72271657
0000000

5151352

221235

291411102210
0000000

2852538

1995192

5109693
000000

5109693

791012131
000000

726581

7475

464714143
0000000

464714143

881648115
0000000

1513571

73151344

0000000
5281224322316

0000000
341161316

341161316

000000
113246

113246

1623893
000000

1623893

2782991
0000000

2782991

0000000
19133415202311

4220813201
0000000

4220813201
0000000

163713

32145671

0000000
15111477310

0000000
15111477310

15111477310

0000000
274132192163221183216

274132192163221183216
14174672715

5514148149
0000000

5514148149

26122418331813
7517122184

313482

16763827

52402840312947
10342211

1311

9861065

10513428

16177815623

44916498

21111

0000000
1234157137

43314105

8114332

9424766
100214037613454

834411

41051283

346733

3126515114

2339913712

229943815

0000000
11571545

11571545

22163311412226
4752852

1121314

37148111

122

14215322137

0000000
32133821282240

196119151423

32838210

105199567

000000
745493

000000
745493

000000
745493

745493

0000000
49410157710891116154283

0021020
49410157710891116154283

0000000
40390153610721103148872

38390152810651099148765

287417

0000200
9203916135211

532524221

39585185

18962125

0000000
645717913213314557

645717913213314557
0040011

00000
25726

25726

0300000
109412836419

1712

612393

35322427414

0000000
1452625241814

11288141011

3318171083

0000200
383810179717933

330915266

144262524161

4122672

2589393

512718131717

13141816844

00
63

63
00

63

01121114
407248478411469524621

38762823
0000000

38762823

0000000
63814594

63814594

3003109
28465533547324

3133413122

99104114

531336

821412333503

0000000
292116143110

292116143110
0000000

292116143110
0000000

292116143110
0000000

292116143110

1001001
368181386340393392556

0000000
1547782154131129380

1547782154131129380
0031001

10428371109049241
1002104

154557181030

67141833541842

211014181721165

30120116
504942434180138

131037102655

18331111212641

1662723102726

0000000
481711060808431

481711060808431
0000000

481711060808431
471711059798427

14

11

0000000
16587194125182179144

22142404
16587194125182179144

14671142112137141119
125148111214

52071215127

31086111014

114175910

1151316769

25104731512

173101111159

33251717298

47115445

1031342188

668211212

321131436

71710765

0000000
47287383310

47287383310

0000000
1371043511

1371043511

0000000
1711222018819

0000000
1711222018819

1711222018819
0000000

0000000
1711222018819

0000000
1711222018819

1711222018819

864331
000000

000000
864331

000000
864331

864331
000000

000000
864331

864331
